# Young maize plants impact the bacterial community in Australian cotton‐sown vertisol more than agricultural practices

**DOI:** 10.1111/1758-2229.13322

**Published:** 2025-04-30

**Authors:** Luc Dendooven, Daniel Ramírez‐Villanueva, Vanessa Romero‐Yahuitl, Karla E. Zarco‐González, Nilantha Hulugalle, Viliami Heimoana, Nele Verhulst, Bram Govaerts, Yendi E. Navarro‐Noya

**Affiliations:** ^1^ Laboratory of Soil Ecology Cinvestav Ciudad de México Mexico; ^2^ New South Wales Department of Primary Industries Australian Cotton Research Institute Narrabri Australia; ^3^ Fenner School of Environment and Society, College of Science Australian National University Canberra Australian Capital Territory Australia; ^4^ International Maize and Wheat Improvement Center (CIMMYT), El Batán Texcoco Edo. de México Mexico; ^5^ School of Integrative Plant Science Cornell University Ithaca New York USA; ^6^ Laboratorio de Interacciones Bióticas, Centro de Investigación en Ciencias Biológicas Universidad Autónoma de Tlaxcala Tlaxcala Mexico

## Abstract

Changes in soil characteristics due to varying farming practices can modify the structure of bacterial communities. However, it remains uncertain whether bacterial groups that break down organic material are similarly impacted. We examined changes in the bacterial community by pyrosequencing the 16S rRNA gene when young maize plants, their neutral detergent fibre fraction, or urea were applied to an Australian Vertisol. This soil was managed with either conventional tillage with continuous cotton, minimum tillage with continuous cotton, or a wheat‐cotton rotation. The soil organic carbon content was 1.4 times higher in the wheat‐cotton rotation than in the conventional tillage with continuous cotton treatment. Approximately 41.6% of the organic carbon was added with maize plants, and 13.1% of the neutral detergent fibre fraction was mineralized after 28 days. The application of young maize plants and the neutral detergent fibre fraction significantly altered the bacterial community and the presumed metabolic functional structure, but urea did not. Many bacterial groups, such as *Streptomyces*, *Nocardioides*, and *Kribbella*, and presumed metabolic functions were enriched by the application of organic material, but less so by urea. We found that a limited number of bacterial groups and presumed metabolic functions were affected in an irrigated Vertisol by the different cotton farming systems, but many were strongly affected by the application of maize plants or its neutral detergent fibre.

## Graphical abstract

In an irrigated Vertisol, different cotton farming systems affected only a few bacterial groups and potential metabolic functions. However, the application of maize plants or their neutral detergent fibre fraction had a significant impact on many of them.

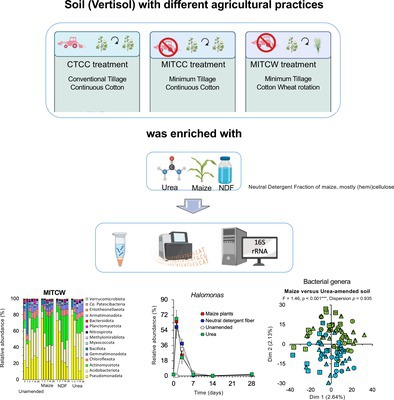

## INTRODUCTION

Microorganisms play a key role organic in matter decomposition and nutrient cycling in soil (Adomako et al., 2022). They are strongly affected by soil properties and soil management, especially in agricultural ecosystems (e.g., Simmons & Coleman, 2008; Trivedi et al., 2016). For instance, 454 pyrosequencing of 16S rRNA genes showed that the bacterial community structure was affected by tillage and residue management practices in a long‐term field experiment in Mexico, (Navarro‐Noya et al., 2013). Application of young maize plants to soil with conventional tilled beds and residue removal or soil with permanent beds and residue retention showed that tillage‐crop residue management defined the bacterial groups involved in the degradation of the organic material (Chávez‐Romero et al., 2016). Additionally, application of inorganic N to the same soil enriched a sequence of bacterial genera characterized as rhizospheric and/or endophytic independent of a yearly application of 0 or 300 kg urea‐N ha^−1^, crop residue management or tillage (Hernández‐Guzmán et al., 2022).

The effects of tillage intensity, different types of cotton varieties, and crop rotations on cotton yields and soil physical and chemical characteristics of irrigated Vertisols have been studied intensively at the Australian Cotton Research Institute, near Narrabri in Northern New South Wales, Australia (Constable et al., 1992; Hulugalle et al., 2005, 2010, 2012, 2013, 2020; Hulugalle & Entwistle, 1997; Tennakoon & Hulugalle, 2006). Typically, when a cotton‐wheat rotation was sown with minimum tillage (‘permanent beds’) improvement in surface and sub‐surface porosity, water holding capacity (WHC), and organic carbon and nitrate‐N stocks, and decreases in subsoil sodicity occurred when compared with soils where cotton was grown in monoculture with conventional tillage. The porosity improvements were due to enhanced wetting/drying cycles caused by the wheat rotation crop and reduction of compaction and smearing by minimizing tillage (McGarry, 1989; Constable et al., 1992; Antille et al., 2016). The improved porosity, in turn, resulted in better drainage and leaching (Hulugalle et al., 2010), and thus, fewer waterlogging events and lower subsoil sodicity (Hulugalle et al., 2005), both of which resulted in deeper water and nutrient extraction by cotton sown after wheat (Hulugalle & Entwistle, 1997). Hulugalle and Entwistle (1997) and Hulugalle et al. (2005) further noted that soil organic matter was greater under minimum tillage and was dominated by the particulate organic matter fraction which was present at far greater concentrations than under conventional tillage, and suggested that this was largely due to less mixing with soil particles resulting in lower rates of microbial decomposition. A similar but parallel process is thought to take place with soil N where volatilization and microbial immobilization were greater under intensive tillage (Constable et al., 1992). Rochester et al. (1993) further suggested that a greater proportion of N taken up by cotton (68%) was from indigenous microbial sources, although this study did not assess different tillage methods. A more recent on‐farm study that assessed transgenic cotton varieties arrived at a similar conclusion (Scheer et al., 2023). In summary, although a large body of research exists with respect to the soil physical and chemical impacts of tillage methods and crop rotations in cotton farming systems sown in Australian Vertisols (Hulugalle & Scott, 2008), there is limited research on the impact of these agricultural practices and soil processes on bacterial community structure and the bacterial groups that are involved in the degradation of organic material, and their interaction with inorganic N. A single study by Coleman et al. (2010) reported that microbial diversity was generally higher in a minimum‐tilled cotton‐wheat‐vetch (*Vicia benghalensis* L.) than in a cotton‐wheat rotation. This was such that Proteobacteria‐*Betaproteobacteria*, *Proteobacteria*‐unclassified bacteria, *Proteobacteria*‐*Alphaproteobacteria*, and Gemmatimonadetes were greater in the vetch rotation. All other studies have focussed on microbial biomass and activity and soil respiration. Polain, Knox, Wilson, Guppy, et al. (2020); Polain, Knox, Wilson, and Pereg (2020) found that relative to cotton‐maize (*Zea mays* L.) microbial biomass and activity under cotton monoculture was greater on the surface but lower in the subsoil, and attributed this to differences in root distribution. They noted, however, that these differences were transient. Nachimuthu et al. (2022) using the cotton strip assay reported that microbial activity in the field during a cotton season was in the order minimum‐tilled cotton‐wheat > minimum‐tilled cotton monoculture > conventionally‐tilled cotton monoculture.

Therefore, the soil was sampled from three treatments at the long‐term field experiment of the Australian Cotton Research Institute (ACRI) (Constable et al., 1992; Hulugalle et al., 2005). The first treatment included soil cultivated with continuous cotton (*Gossypium hirsutum* L.) and conventionally tilled (considered the CTCC treatment). The second treatment included soil cultivated with continuous cotton but with minimum tillage (considered the MITCC treatment) while the third treatment included soil cultivated with cotton wheat (*Triticum aestivum* L.) in rotation and minimum tillage (considered the MITCW treatment). Each soil was amended with maize plants, its neutral detergent fibre (NDF) fraction (mostly (hemi)cellulose), or urea and incubated for 28 days while emissions of CO_2_, soil mineral N content, and the bacterial community were monitored. An unamended soil served as control. The authors hypothesized that the application of organic material would have a larger effect on the bacterial community and its functionality than the different agricultural practices applied to the soil. In the proposal to investigate this hypothesis, the objectives of this study were to determine how cultivation practices (minimum tillage versus normal tillage and monoculture versus wheat cotton rotation) affected the bacterial community structure and its putative metabolic functions in the Australian Vertisol, and (ii) how the bacterial community structure and their functionality was affected by the application of organic material (NDF or maize plants) or urea in the same soil.

## EXPERIMENTAL PROCEDURES

### Experimental site

The experimental site is located at the ACRI, near Narrabri (149°47′ E, 30°13′S) in New South Wales's main cotton production area. The site has a subtropical semi‐arid climate, BSh (Kottek et al., 2006) with January as the warmest month (mean daily maximum temp 35°C) and a mean annual rainfall of 593 mm. According to the Soil Survey Staff (2003), the soil at the experimental site is classified as a fine, thermic, smectitic, typic haplustert with a particle size distribution of 640 g clay kg^−1^, 110 g silt kg^−1^, and a 250 g sand kg^−1^ (Hulugalle et al., 2020) in the 0–1 m soil layer.

### Experimental treatments and sampling

The three treatments selected for this study were initiated at the start of the field experiment in 1985. A detailed description is given in Constable et al. (1992) and a summary in Table S1. They were as follows: (1) conventional tilled continuous cotton (*Gossypium hirsutum* L.), with cotton planted in October every year, incorporating the cotton plants after harvest by disc‐ploughing to 0.2 m, chisel ploughing to 0.3 m and the construction of 1‐m beds to a height of 0.15 m (considered the CTCC treatment); (2) minimum tilled continuous cotton in permanent raised 1‐m beds, the cotton plants were slashed after harvest, root cut and incorporated, and followed by reformation of beds with a disc‐hiller (considered the MITCC treatment); (3) The minimum tilled cotton‐winter wheat (*Triticum aestivum* L.) rotation (summer cotton‐winter wheat‐summer and winter fallow‐summer cotton) on 1‐m permanent raised beds, where until 1999 wheat stubble was incorporated before planting conventional cotton (considered the MITCW treatment). Since 2000 the wheat stubble was retained as standing stubble and Round‐up Ready cotton sown until the 2005–06 season, and ‘Bollgard‐Roundup Ready Flex’ cotton thereafter. The beds (rows) were spaced at 1‐m intervals with vehicular traffic restricted to the furrows (Hulugalle et al., 2005, 2020). The abovementioned treatments were arranged in a randomized complete block design with 4 replications. Individual plots were 190 m long and 12–20 rows wide (Hulugalle et al., 2020). Fertilizer and irrigation management practices for the site are reported in Hulugalle et al. (2005) and follow the recommended irrigation and crop management practices for Australian cotton production systems (Australian Cotton Industry Development and Delivery Team, 2013; Serafin et al., 2011).

A composite soil sample, based on 20 sampling points, was collected with a spade from the 0–10 cm layer of each plot (*n* = 3) of the three treatments (*n* = 3) on the 29 October 2012 (Figure S1). After 30 years, the soil organic C content was higher in the minimum tilled soil with crop rotation and residue retention (MITCW treatment) than in the conventional tilled with cotton monoculture and residue removal (CTCC) (Hulugalle et al., 2020). The samples from each plot and treatment were pooled separately and transported to the Laboratory of Soil Ecology at Cinvestav (Mexico) for further investigations and analyses. As such, nine soil samples were obtained and different treatments were applied to each of the soil samples to avoid pseudo‐replication (Heffner et al., 1996).

### Cultivation of maize plants used in the aerobic incubation

The cultivation of the maize plants has been described previously by Ramirez‐Villanueva et al. (2015). Briefly, an acrylic chamber of 105 L (surface 35 cm × 50 cm and 60 cm high) was used for the cultivation of the maize plants. Maize seeds were surface sterilized with 1.5% (v/v) sodium hypochlorite for 12 min and washed thoroughly with sterile distilled water. Seeds were germinated on 0.8% agar‐water plates to induce etiolation and incubated in the dark at 28°C for 48 h. The maize seedlings with roots of approximately 2 cm were placed on sterilized and C‐free vermiculite in the growth chamber and moistened with a nutritive Steiner (1961). After 25 days, the maize plants were harvested, air‐dried, and characterized.

The maize plants were fractionated according to the Van Soest method (Van Soest, 1963; van Soest & Wine, 1967) as described in Ruíz‐Valdiviezo et al. (2010). Hot extraction with a neutral detergent solution removed the ‘soluble’ part of the maize residue, leaving a NDF fraction containing most of the cell wall constituents, that is, (hemi)cellulose plus some lignin. The characteristics of the maize and its NDF fraction are given in Table S2.

### Aerobic incubation of soil amended with maize residue, NDF, or urea, or left unamended

One kg soil of each sample was adjusted to field capacity (40 g, 100 g^−1^) with distilled water and pre‐incubated for 1 week in a cylindrical 70 L drum containing a 1 L container with distilled water to avoid desiccation and one with 1 L 1 M NaOH to capture CO_2_ emitted. After 1 week, each soil sample was analysed for pH, electrolytic conductivity (EC), WHC, particle size distribution, carbon, and total nitrogen as described below.

Twenty‐eight 25 g sub‐samples from each soil sample (*n* = 9, three treatments, and three replicated plots) were added separately to 120 mL glass flasks, and four treatments were applied. Seven sub‐samples were amended with 100 mg dried young maize plants (considered the maize treatment), seven with 100 mg NDF (obtained from 25‐day‐old maize plants that had been fractionated according to the Van Soest method; Van Soest, 1963) to obtain the NDF fraction (van Soest & Wine, 1967), seven with 200 mg urea‐N kg^−1^ dry soil (considered the urea treatment), and seven were left unamended and served as control treatment. The amount of young maize plants or their NDF fraction applied to soil was equivalent to 2 mg C kg^−1^. All data reported are on a soil dry weight basis.

The aerobic incubation described was based on the method developed by Jenkinson and Powlson (1976) to measure the microbial biomass C in soil. Each flask was placed in a 1 L glass jar containing a flask with 20 mL 0.5 M NaOH to capture the emitted CO_2_ and an additional flask with distilled water to avoid desiccation of soil during incubation. After 0, 1, 3, 7, 14, and 28 days, the jars were opened, and the flask with NaOH was taken out and analysed for CO_2_. The soil was removed from the flasks and 6 g soil was used to extract DNA as described below, while the rest was used to extract mineral N (NH_4_^+^, NO_2_^−^, NO_3_^−^) with 100 mL 0.5 M K_2_SO_4_. The K_2_SO_4_ extract was analysed for mineral N on a San Plus System‐SKALAR automatic analyser (Skalar, Breda, the Netherlands) (Mulvaney, 1996).

### Soil characterization

The pH was determined in a 1:2.5 soil/H_2_O suspension using a 716 DMS Titrino pH metre (Metrohm Ltd. CH.‐901, Herisau, Switzerland) fitted with a glass electrode (Thomas, 1996). Total C was measured by oxidation with potassium dichromate (K_2_Cr_2_O_7_) and titration of excess dichromate with ammonium ferrosulfate [(NH_4_)_2_FeSO_4_] (Kalembasa & Jenkinson, 1973). Total N was determined by the Kjeldhal method using concentrated H_2_SO_4_, K_2_SO_4_, and HgO to digest the sample (Bremner, 1996). The hydrometer method was used to determine the soil particle size distribution (Gee & Bauder, 1986). The CO_2_ in the 1 M NaOH was determined by titration with 0.1 M HCl (Jenkinson & Powlson, 1976). The WHC was measured on water‐saturated soil samples added to a funnel and left overnight. The soil was drained freely and the WHC was defined by differences in weight between the drained wetted soil and the dry soil.

### DNA extraction, PCR amplification of bacterial 16S rRNA genes, and analysis of pyrosequencing data

Fulvic and humic acids were removed from the 6 g soil sample with 0.15 M sodium pyrophosphate and 0.15 M phosphate buffer pH 8 (Ceja‐Navarro et al., 2010). Three different techniques were used to extract the metagenomic soil DNA. Two g of soil was used for each technique. The first method, using a thermal shock to disrupt the bacterial cells, was based on the technique developed by Ceja‐Navarro et al. (2010). The second method used a surfactant solution and mechanical disruption of the bacterial cells (Hoffman & Winston, 1987), while the third method was based on enzymatic lysis of cells (Sambrook & Russell, 2001). The DNA obtained from each technique was pooled in a single DNA sample. As such, 18 g (2 g × 3 techniques × 3 plots) from each treatment (*n* = 4) and soil (*n* = 3) was extracted for DNA on each sampling day (*n* = 6).

The V1–V3 region of 16S rRNA bacterial genes were amplified with 10‐pb barcode primers [8‐F (5′‐AGA GTT TGA TCI TGG CTC A‐3′) and 556‐R (5′‐TGC CAG IAG CIG CGG TAA‐3′)] and containing the A and B 454 FLX adapters. The PCR reactions and the DNA purification and quantification were done as described by Navarro‐Noya et al. (2013). The DNA sequencing was done by Macrogen Inc. (Sequencing Service, Seoul, Korea) using a Roche 454 GS‐FLX Titanium pyrosequencer (Roche, Manheim, Germany).

The QIIME software version 2‐2022.8 was used to analyse the sequences (Bolyen et al., 2019). The q2‐demux plugin was used for demultiplexing the sequencing runs and denoising was done with DADA2 (Callahan et al., 2016). A taxonomic assignment was made using amplicon sequence variants (ASVs) with the q2‐feature‐classifier and classify‐sklearn native Bayes against SILVA database version 138.99 (Quast et al., 2013).

### Bacterial functionality

The putative metabolic functions were determined with PICRUSt version 1.1.2, using the KEGG (Kyoto Encyclopedia of Genes and Genomes) database for annotations (Langille et al., 2013).

### Statistical analysis

All statistical analyses were done in R v4.2.2 (R Core Team, 2022) within the RStudio environment (Version 2023.09.0 + 463). An ANOVA test (aov function) was used to determine the effect of agricultural practices on soil characteristics. The effect of agricultural practice and treatment (application of maize plants, urea, and NDF) on the emission of CO_2_ and mineral N after 28 days was determined with an ANOVA analysis.

Alpha diversity of soil bacterial community was determined based on the Hill numbers at different *q* orders (at *q* = 0, 1, and 2) (Chao et al., 2010). The Hill number at *q* = 0 gives the ASVs richness, *q* = 1 is the Shannon entropy and denotes frequently occurring ASVs and *q* = 2 is the inverse Simpson and characterizes dominant ASVs (Chao et al., 2014) and they were calculated with the HillR package v. 0.5.1, Li, 2021. A non‐parametric analysis (t1way test of the WRS2 package, v. 1.1‐0, Mair & Wilcox, 2020) was used to determine the effect of agricultural practices (CTCC, MITCC, and MITCW), treatment (maize plants, NDF, urea, and unamended soil), and time (day 0, 1, 3, 7, 14, and 28) on the Hill numbers. The changes in the bacterial community due to the application of maize plants, the NDF fraction or urea versus that in the unamended soil, that is, phylogenetic beta diversity, in the CTCC, MITCC, and MTCW treatments on day 0, 1, 3, 7, 14, and 28 were determined with the betapart R package (Baselga & Orme, 2012).

Ordination (principal component analysis, PCA) and multivariate comparison (perMANOVA) were done with converted sequence data using the centred log‐ratio transformation test returned by the aldex.clr argument ALDEx2 (v: 1.21.1) (Gloor et al., 2020). The FactoMineR (v. 2.3) package (Husson et al., 2020) was used for the PCA and the vegan (v. 2.5‐6) package to determine the homogeneity of group dispersions (dispersion within soils or treatments) (Anderson, 2017; Oksanen et al., 2019).

The effect size, which is defined as the difference between groups divided by the maximum dispersion within group A or B, was calculated after a centred log‐ratio transformation with the aldex.ttest argument (ALDEx2 (version, 1.18)), Gloor et al. (2020). A negative value indicates that the relative abundance of the microbial group was higher in the first considered treatment than in the second one. The effect size was calculated by comparing the bacterial groups and putative metabolic functions in the unamended CTCC soil with the unamended MITCC and MITCW soils incubated for 0, 1, 3, 7, 14, and 28 days. Additionally, the effect size was calculated comparing the bacterial groups and putative metabolic functions in the unamended CTCC, MITCC, and MITCW soils with the same soils amended with maize, the NDF fraction or urea after 1, 3, 7, 14, and 28 days. These calculations allowed us to determine if maize plants, the NDF fraction, or urea had a similar effect on the bacterial groups or putative metabolic functions in each soil (CTCC, MITCC, and MITCW) and if the effect was consistent over time (1, 3, 7, 14, and 28 days). Only large effect sizes (≤ −0.8 or ≥ 0.8) for the bacterial groups or very large (≤ −1.4 or ≥ 1.4) for the putative metabolic functions (Kim, 2015) were reported. Only very large (≤ −1.4 or ≥ 1.4) effect sizes were reported for the putative metabolic functions as most of them were affected strongly by the application of maize plants or the NDF fraction.

## RESULTS

### Soil characteristics and C and N mineralization

The soil organic C and WHC were significantly lower in the CTCC than in the MITCW treatment (*p <* 0.05) (Table 1). The other soil characteristics did not significantly differ among the agronomic management practices.

**TABLE 1:** Characteristics of soil (0–0.10 m) cultivated with cotton (*Gossypium hirsutum* L.) monoculture (summer cotton‐winter, fallow‐summer cotton) conventional tillage (CTCC), minimum tillage of continuous cotton (MITCC), and minimum tillage cotton‐wheat (*Triticum aestivum* L.) rotation (summer cotton‐winter wheat‐summer and winter fallow‐summer cotton) (MITCW).

Agricultural		EC	Total N	Organic C	WHC	Sand (<2000–50 μm)	Clay (<2 μm)	Silt (< 50–2 μm)	USDA textural
Practice	pH	(dS m^−1^)	(g kg^−1^ soil)						Classification
CTCC	7.4^a^ A^b^	0.14 A	0.86 A	6.79 B	428 B	260 A	580 A	160 A	Clay
MITCC	7.2 A	0.13 A	0.98 A	8.30 AB	510 AB	360 A	470 A	170 A	Clay
MITCW	7.3 A	0.32 A	1.03 A	9.34 A	529 A	340 A	510 A	150 A	Clay
*F*‐value	4.58	3.90	1.96	7.05	5.53	1.43	1.78	0.10	
*p*‐value	0.062	0.82	0.221	0.027	0.044	0.310	0.247	0.903	

Abbreviations: EC, electrolytic conductivity; WHC: water holding capacity.

^a^ Mean of three replicate plots.

^b^ Values with the same capital letter are not different significantly between the agricultural practices, that is, within the columns (*p* < 0.05).

**FIGURE 1:**
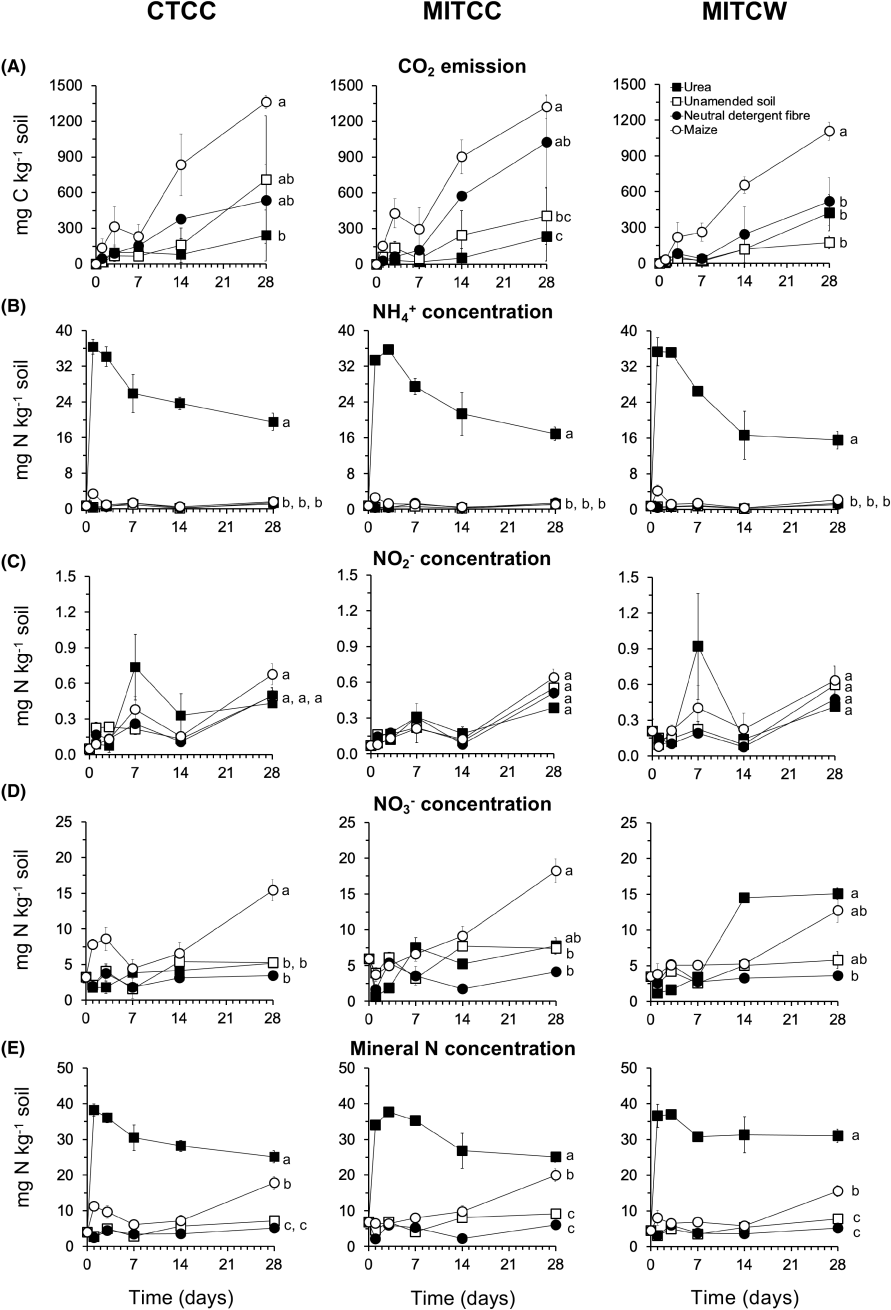
(A) Emission of CO_2_ (mg C kg^−1^ dry soil), and (B) the concentration of ammonium (NH_4_^+^), (C) nitrite (NO_2_^−^), (D) nitrate (NO_3_^−^) and (E) mineral N (sum of NH_4_^+^, NO_2_^−^, NO_3_^−^) (mg N kg^−1^ dry soil) in soil cultivated with cotton (*Gossypium hirsutum* L.) monoculture (summer cotton‐winter, fallow‐summer cotton) conventional tillage (CTCC), minimum tillage of continuous cotton (MITCC), and minimum tillage cotton‐wheat (*Triticum aestivum* L.) rotation (summer cotton‐winter wheat‐summer and winter fallow‐summer cotton) (MITCW) left unamended (□) or amended with young maize plants (*Zea mays* L.) (□), its neutral detergent fibre (NDF) fraction (●) or urea (■) incubated aerobically at 22 ± 2°C for 28 days. Values with the same letter are not significantly different at day 28 (*p* < 0.05).

The application of maize plants increased the emission of CO_2_ in the CTCC, MITCC, and MITCW soil after 28 days, but the effect of the application of NDF or urea varied between the soils (Figure 1A). On average, 41.6% of the organic C added with maize| plants and 13.1% with the NDF fraction were mineralized after 28 days.

The concentration of NH_4_^+^ remained low and was similar in the CTCC, MITCC, and MITCW unamended soils and soils amended with NDF or maize plants (Figure 1B). It increased sharply, however, in all the urea‐amended soils to approximately 36 mg NH_4_^+^‐N kg^−1^ soil on day 1. The concentration of NH_4_^+^ decreased slowly after day 3 but was still ≥16 mg NH_4_^+^‐N kg^−1^ soil after 28 days. The concentration of NO_2_^−^ was similar in all soils and increased over time, but remained <1 mg NO_2_^−^‐N kg^−1^ soil after 28 days (Figure 1C). The application of maize plants increased the concentration of NO_3_^−^ in the CTCC, MITCC, and MITCW soils compared to the unamended soils (Figure 1D). The mineral N concentration (sum of NH_4_^+^, NO_2_^−^ and NO_3_^−^) increased sharply in the CTCC, MITCC, and MITCW soils amended with urea at day 1 and showed some small decreases afterwards. It showed an increase in the maize plants amended soils compared to the unamended soils after day 14, but not when the NDF fraction was applied to the soil (Figure 1E).

### Alpha and beta diversity

The Hill numbers showed the same dynamics in all soils independent of agricultural practice or treatment applied to soil (Figure S2a). They were mostly constant over time, but Hill numbers at *q* = 0 were lower on day 1 than on the other days, while those at *q* = 1 and *q* = 2 were lower on day 1 or 3 than on the other days. The agricultural practices and treatments applied had no significant effect on the Hill numbers, but time did (Table S3). The bacterial diversity (Hill number *q* = 0) was significantly lower after 1 day compared to the other days (mean of all soils and treatments), while the frequent and dominant ASVs were significantly lower on days 1 and 3 compared to the other days (*p* < 0.05). The Jaccard pair‐wise dissimilarity was similar in soil independent of agriculture practices or treatment applied and showed little variation over time (Figure S2b). The beta diversity analysis indicated that most of the changes in ASVs were due to 1‐to‐1 replacement (turnover) although some loss of species also occurred, that is, species were not detected.

### Bacterial community structure and putative metabolic functions in the unamended soil

**FIGURE 2:**
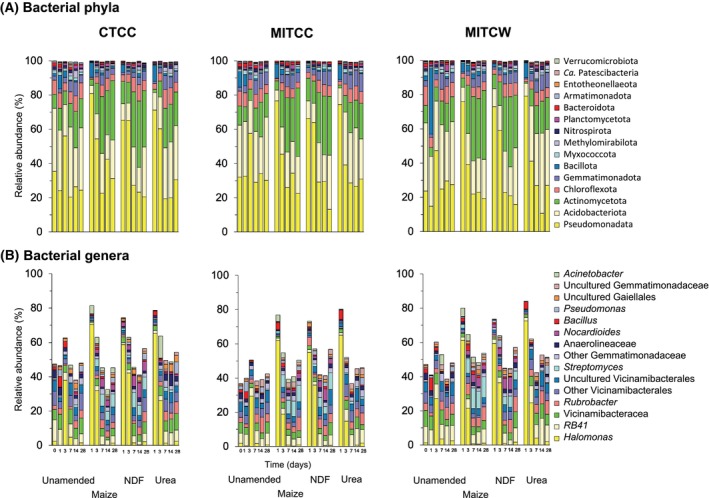
Bar plots with the relative abundances (%) of the 15 most abundant bacterial phyla and genera found in soil with different agricultural practices amended with young maize plants (maize, *Zea mays* L.), its neutral detergent fibre (NDF) fraction or urea, or left unamended and incubated aerobically at 22 ± 2°C for 28 days. The explanation of the abbreviations of the agricultural practices can be found in the legend in Figure 1.

**FIGURE 3:**
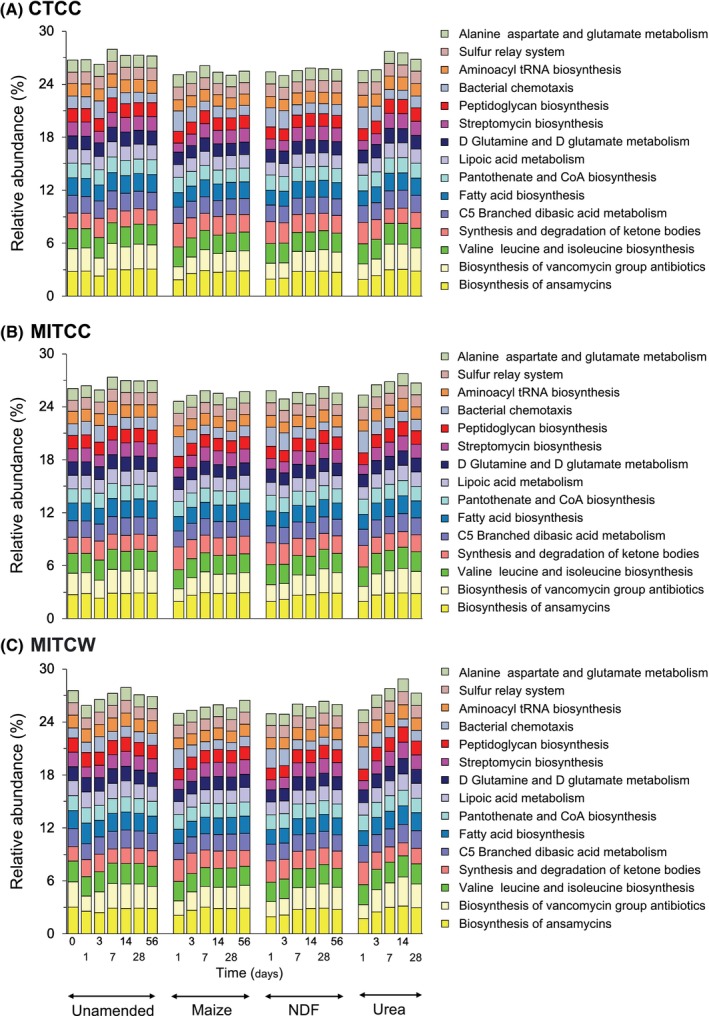
Bar plots with the relative abundances (%) of the 15 most abundant putative metabolic functions found in soil with different agricultural practices amended with young maize plants (maize, *Zea mays* L.), its neutral detergent fibre (NDF) fraction or urea or left unamended and incubated aerobically at 22 ± 2°C for 28 days. The explanation of the abbreviations of the agricultural practices can be found in the legend in Figure 1.

Of the 32 detected bacterial phyla, Pseudomonadota (formerly Proteobacteria) was the most abundant (relative abundance 31.7%), followed by Acidobacteriota (31.2%) and Actinomycetota (formerly Actinobacteria) (12.4%) (Figure 2A). RB41 (Acidobacteriota) was the dominant bacterial genus in the unamended soils with a relative abundance of 7.5%, followed by *Halomonas at* 6.4% and members of Vicinamibacteraceae (Acidobacteriota) at 6.3% (Figure 2B). Biosynthesis of vancomycin group antibiotics (2.7%) and ansamycins (2.3%) were the most abundant putative metabolic functions (Figure 3).

The PCA did not separate the different agricultural practices considering bacterial phyla, genera or ASVs, or the putative metabolic functions (Figure S3). The perMANOVA test indicated a significant effect of agricultural practices on the bacterial community structure considering all phyla, but not when considering all bacterial groups assigned up to the taxonomic level of genus, ASVs, or the putative metabolic functions in the unamended soil. However, the application of the different agricultural practices had a large effect on the relative abundance of some bacterial groups when comparing the different unamended soils, that is, the effect size was large (≤ −0.8 or ≥ 0.8) (Table S4). For instance, the relative abundance of some bacterial groups was different at the onset of the experiment, for example, *11–24* (Acidobacteriota) and *UTCFX1* (Chloroflexota), and that difference was maintained for some bacterial groups, for example, *RB41* (Acidobacteriota), for the entire incubation (Figure S4). For others, the differences in relative abundance between soils with different agricultural practices were small at the onset of the experiment but became large after 3 or 7 days, for example, other Vicinamibacteraceae and Vicinamibacteriales (Acidobacteriota) and *Rubrobacter*. The application of the different agricultural practices had a large effect on the relative abundance of many putative metabolic functions in the unamended soils and most were in the first 2 weeks (Figure S5 and Table S5.).

### Bacterial community structure and putative metabolic functions in the maize plant amended soil

**FIGURE 4:**
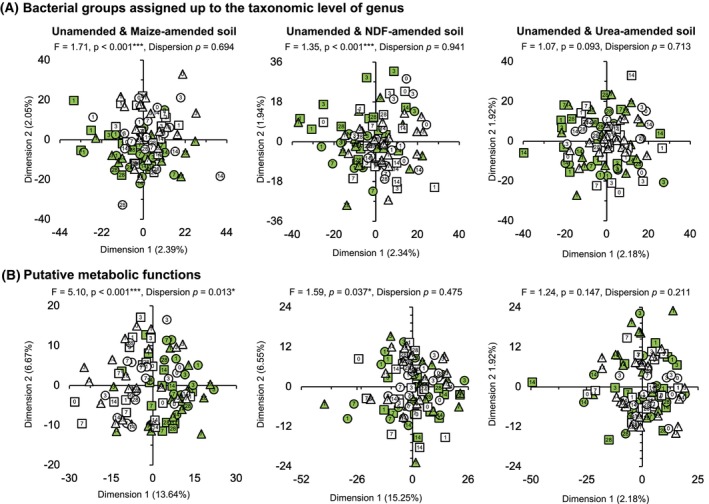
Principal component analysis (PCA) with (A) all the bacterial groups assigned up to the taxonomic level of genus and b) the putative metabolic functions in the unamended CTCC (□), MITCC (○) and MITCW (△) soils versus the CTCC (■), MITCC (●) and MITCW (▲) soils amended with young maize plants, its neutral detergent fibre (NDF) fraction or urea. The values in the symbols are the number of days the soil was incubated aerobically. The explanation of the abbreviations of the agricultural practices can be found in the legend in Figure 1.

**FIGURE 5:**
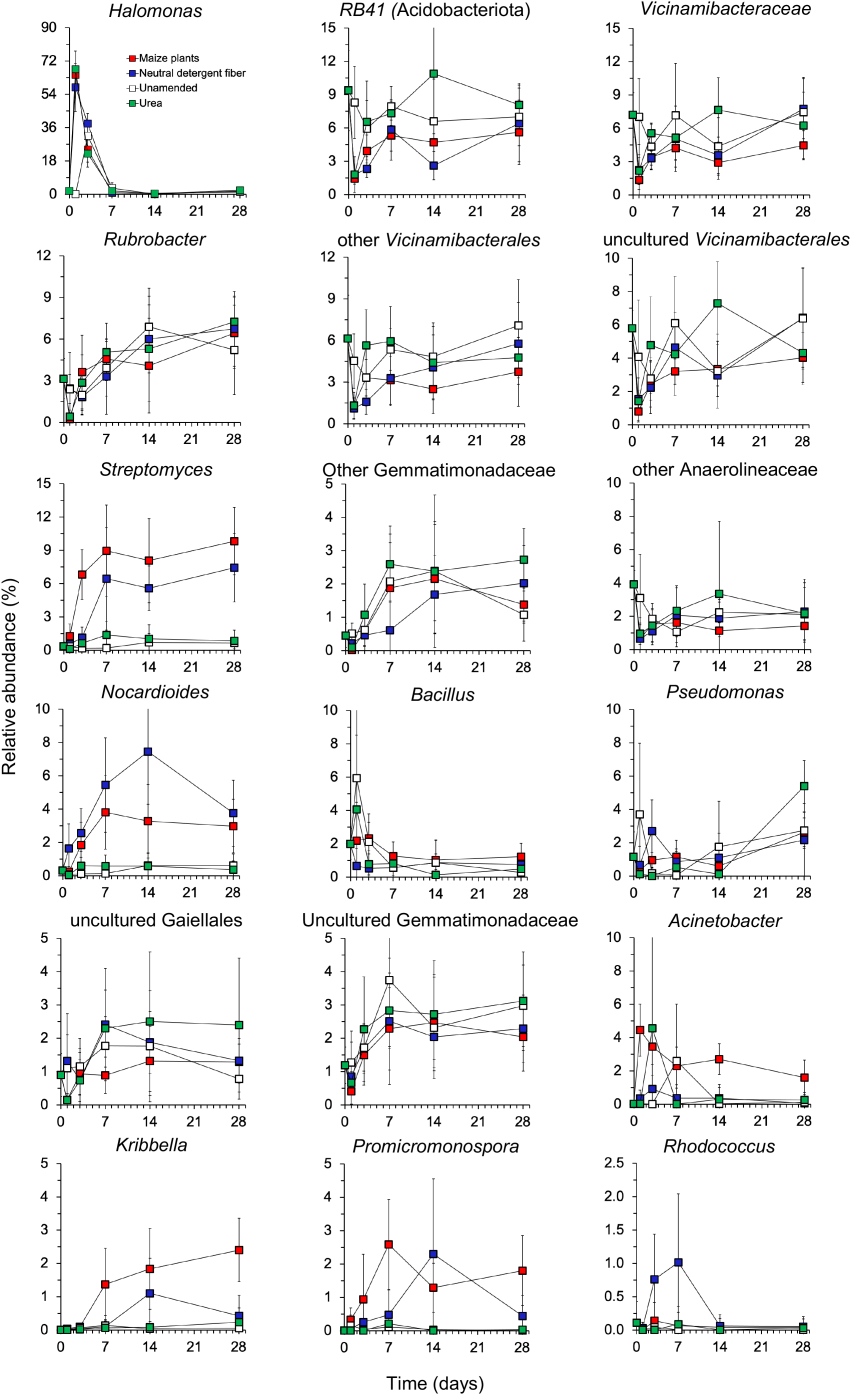
Changes in the relative abundance (%) of the most abundant bacterial groups assigned up to the taxonomic level of the genus in soil (mean of the three soils with different agricultural practices) left unamended (□) or amended with maize (*Zea mays* L.) (■), its neutral detergent fibre (NDF) fraction (■) or urea (■) incubated aerobically at 22 ± 2°C for 28 days.

The bacterial community structure in the maize plant amended soils was significantly different from that in the unamended soils (*p* < 0.001) (Figure 4A). No significant difference in dispersion (variances) was detected between the maize plants amended soil and the unamended soil (*p* = 0.694). The relative abundance of some bacterial groups showed large changes over time when maize plants were applied to the soil compared to the unamended soil (Figures 5 and S6). For instance, the relative abundance of Acidobacteriota was lower in the maize‐amended soil than in the unamended soil and that of Actinomycetota was larger (Figure S6). A large increase in the relative abundance of *Streptomyces*, *Nocardioides*, and *Kribbella* was also detected when maize plants were applied to the soil compared to the unamended soil (Figure 5). Consequently, the effect of the application of young maize plants on the relative abundance of bacterial groups assigned up the taxonomic level of the genus was often very large (size effect ≤ −1.4 or ≥ 1.4, Figure S7a) and significant (Table S6.).

Application of maize plants had a significant effect on the putative metabolic functions compared to the unamended soil (*p* < 0.001), but the dispersion (variance) was also significantly different between the NDF‐amended and unamended soil (*p* = 0.013, Figure 4B). The relative abundance of most putative metabolic functions showed large changes (effect size ≤ −0.8 or ≥ 0.8) when maize plants were applied to the soils compared to the unamended soils after 1, 3, 7, 14, and 28 days (Figure S8a). The relative abundance of most of the putative functions decreased when young maize plants were applied to soil compared to the unamended soil and only a limited number of them showed a constant increase over time, for example, beta‐lactam resistance, biosynthesis of type II polyketide backbone and siderophore group nonribosomal peptides (Table S7.). The changes in the relative abundances of the putative metabolic functions in the maize‐amended soil over time were mostly small, although some fluctuations occurred in the first week (Figure S9a).

### Bacterial community structure in the NDF‐amended soil

Application of NDF changed the bacterial community structure significantly compared to the unamended soil (*p* < 0.001) (Figure 4A). The dispersion (variances) was not significantly different between the NDF‐amended and unamended soil (*p* = 0.768). The relative abundance of some bacterial groups showed large significant changes when NDF was applied to soil compared to the unamended soil (*p* < 0.05, Figure S7b, Table S6.). The relative abundance of some bacterial groups increased sharply when NDF was applied to the soil compared to the unamended soil and they were mostly the same bacterial groups as when maize plants were added, for example, Actinomycetota, *Streptomyces*, *Nocardioides*, and *Kribbella* (Figures 5 and S6).

Application of NDF had a significant effect on the putative metabolic functions compared to the unamended soil (*p* = 0.037) and the dispersion was not significantly different between the NDF‐amended and unamended soil (*p* = 0.475, Figure 4B). The relative abundance of most putative metabolic functions showed large changes (effect size ≤ −0.8 or ≥ 0.8) when the NDF fraction was applied to the soils compared to the unamended soils after 1, 3, 7, 14, and 28 days (Figure S8b). The relative abundance of most of the putative functions decreased when the NDF fraction was applied to soil compared to the unamended soil and only a limited number of them showed a constant increase over time, for example, beta‐lactam resistance, biosynthesis of type II polyketide backbone and siderophore group nonribosomal peptides (Table S7.). The changes in the relative abundances of the putative metabolic functions in the NDF‐amended soil over time were mostly small, although some fluctuations occurred in the first week (Figure S9b).

### Bacterial community structure in the urea‐amended soil

The bacterial community structure was similar in the urea‐amended and the unamended soil (Figure 4A). The application of urea had a limited effect on bacterial groups compared to the unamended soil and only eight bacterial groups assigned up to the taxonomic level of genus were significantly affected by it (*p* < 0.05, Figure S7c, Table S6.).

Application of urea had no significant effect on the putative metabolic function structure compared to the unamended soil (Figure 4B). The relative abundance of most putative metabolic functions showed only small changes when urea was applied to the soils compared to the unamended soils after 3, 7, 14, and 28 days, but not on day 1 when most of them showed large changes (Figure S9c). The changes in the relative abundances of the putative metabolic functions in the urea‐amended soil over time were mostly small, although some fluctuations occurred in the first week (Figure S9).

### Comparison of the bacterial communities and putative metabolic functions in the maize, NDF, and urea‐amended soil

The bacterial communities in the maize plants, NDF, and urea‐amended soils were significantly different (*p* < 0.05), and the dispersion analysis indicated the dispersion was not significantly different between them (Figure S10a). The putative metabolic functions in the maize plants, NDF, and urea‐amended soils were significantly different between them, but also the dispersion except when comparing the NDF‐amended soil with the urea‐amended soil (*p* < 0.05) (Figure S10b).

Different patterns emerged when comparing the effect of maize or NDF on bacterial groups compared to the unamended soil. First, the application of maize or NDF did not affect the relative abundance of the bacterial group, for example, *Rubrobacter*. Second, the bacterial group was more enriched by the application of maize plants than when NDF was applied, for example,*Acinetobacter*, *Streptomyces*, and *Kribbella*. Third, the bacterial group was more enriched by the application of NDF than when maize plants were applied, for example, *Rhodococcus* and *Nocardioides*. Fourth, both similarly enriched the bacterial group, for example, *Promicromonospora*, and fifth, the relative abundance of the bacterial group was reduced similarly by the application of both maize plant and NDF, for example, *RB41* (Acidobacteriota).

### The effect of agricultural practices on the bacterial groups and the putative metabolic groups

The effects of agricultural practices on the putative metabolic functions were different from those on bacterial groups (Table S8.). First, the variation in putative functions among replicated plots was smaller than those of bacterial groups. Second, the effect of agricultural practices on the relative abundances of the putative metabolic functions occurred mostly in the first 2 weeks while large changes were still detected in the relative abundance of bacterial groups after 4 weeks. Third, between 2% and 5% of the 665 detected bacterial groups assigned up to the taxonomic level of the genus were strongly affected by agricultural practices (large effect size ≤ −0.8 or ≥ 0.8), but between 1 and 68% of the 180 detected putative metabolic functions. Fourth, the changes over time in the relative abundances of the putative metabolic functions were smaller than those of bacterial groups.

## DISCUSSION

### Soil characteristics, C and N mineralization

Soil organic matter content is an important indicator of soil quality (Gerke, 2022; Šimanský et al., 2019). Soil organic matter improves soil physicochemical characteristics, serves as an energy source for heterotrophic microbial activity, and upon mineralization provides nutrients for plants (Wander et al., 2019). Conservation agricultural practices, which include minimum tillage, crop residue retention, and crop diversification as core elements (FAO, 2001, 2023), can significantly increase the organic matter content compared to agricultural soils under conventional agricultural practices with intensive tillage and crop residue removal (Macray & Montgomery, 2023; Page et al., 2020). In this study, the organic C content in the minimum tillage systems with crop rotation and residue retention was 1.4 times higher compared with the more conventional system. The incorporation of crop residue brings it in direct contact with soil microorganisms and facilitates its degradation. Additionally, tillage breaks up soil aggregates liberating physically protected organic material that becomes available for microorganisms further reducing the soil organic C content (Kan et al., 2022). When crop residues are left on the soil surface, their contact with soil organisms is reduced and their degradation is delayed so soil organic C content increases, such as in the MITCW treatment, compared to treatments where they are incorporated, for example, CTCC treatment.

The application of organic material increases the C substrate for soil microorganisms. Its chemical composition and C‐to‐N ratio define the amount mineralized (Lazicki et al., 2020). Organic material can be resistant to mineralization when the content of recalcitrant components, such as lignin, is high or its N content is too low for the microorganisms that degrade it (Vahdat et al., 2011). The maize plants applied to the soil in this study were young so the amount of organic material resistant to degradation was low, that is, lignin content was 2.5%, and the C‐to‐N ratio was low 12.4 so a lack of mineral N was not impeding their mineralization. As such, nearly half of the C of the maize plants was mineralized within 28 days. The mineralization of the NDF fraction was lower (13%) than that of the young maize plants (42%) after 28 days. The C‐to‐N ratio of NDF was higher (21.3) and the easily decomposable organic material, for example, short‐chained carbohydrates and proteins, was removed. As such, the NDF fraction contained organic material more resistant to degradation, that is, (hemi)cellulose (77.6%) and lignin (6.3%), than the young maize plants. Consequently, its C mineralization was lower compared to that of the young maize plants after 28 days.

Although the crops in the field experiment were regularly fertilized, the soil was N‐depleted. As 42% of the 2000 mg maize organic C was mineralized (840 mg CO_2_‐C) after 28 days approximately 67.6 mg of the organic N in the maize plants (C/N ratio of 12.4) should be mineralized. However, only 16.4 mg mineral‐N was recovered after 28 days so it can be assumed that approximately 51.2 mg N was immobilized by microorganisms. A similar process occurred in urea‐amended soil. Of the 93 mg N applied with urea, on average only 22 mg mineral N was recovered, and the rest appeared to be immobilized by the microorganisms after 28 days if we assume that no mineral N was lost through denitrification or NH_3_ volatilization. The soil was incubated aerobically so losses of NO_3_^−^ through denitrification should be low (Li, Tang, et al., 2022) and the urea was mixed immediately into the soil reducing NH_3_ volatilization (Li, Wang, et al., 2022). Losses of mineral N through other biotic, for example, N_2_O emission during nitrification (He et al., 2020), or abiotic processes, for example, NH_4_^+^ fixation on the soil matrix (Zhang et al., 2007), cannot be excluded but are normally small.

Application of inorganic fertilizer usually has no effect on C mineralization in soil (e.g., Guo et al., 2019; Li et al., 2018), but not always (Hernández‐Guzmán et al., 2022). For instance, Guo et al. (2019) studied the role of bacteria in C mineralization in yellow paddies and found that chemical fertilizer application had no significant effect on CO_2_ emissions, potential mineralized carbon, and turnover rate constant, but organic‐fertilizer treatments did. Li et al. (2018) reported that chemical fertilizer application alone did not alter the labile C fractions, soil microbial communities and SOC mineralization rate compared to unfertilized soil, but straw in a wheat‐maize double cropping system in Northern China did. While studying the bacterial community in a Mexican Vertisol, the application of NH_4_^+^ stimulated the C mineralization and one‐third of the 300 mg NH_4_^+^–N was immobilized (Hernández‐Guzmán et al., 2022). We speculated that this was due to the high C:N ratio of crop residues left in the field. For instance, Kamkar et al. (2014) reported a C:N ratio of 32.3 for cotton, 49.6 for corn (maize), and 60.5 for wheat in a study on the effect of crop residues on soil nitrogen dynamics and wheat yield, while the USDA Natural Resources Conservation Service reported a C:N ratio of approximately 80 for wheat and 57 for maize (soils.usda.gov/sqi). In this study, immobilization of mineral N occurred, but the application of mineral N did not stimulate the C mineralization. The C:N ratio of cotton is normally lower than that of maize or wheat so N immobilization should be lower when cotton residue is left in the field (this study) than when wheat or maize residue is (Hernández‐Guzmán et al., 2022).

### The bacterial community structure in the unamended soil

It has often been reported that conservation agriculture increases soil bacterial richness and diversity, while conventional agricultural practices, for example, ploughing and crop monoculture, negatively affect them (Khmelevtsova et al., 2022; Pratibha et al., 2023). For instance, Wang et al. (2016) found a 3.8‐fold increase in the Simpson index, that is, a measure of diversity that includes the number of species and the relative abundance of each species, when comparing the bacterial community in a soil under conservation agriculture (no tillage) with that under 5‐year tillage. In this study, however, the different agricultural practices did not affect the bacterial diversity (Hill number at *q* = 0). The intensity and combination of the agricultural practices applied will determine how much the bacterial diversity is affected by them, but soil characteristics, for example, pH, might alter the effects of the agricultural practices applied (Shu et al., 2022).

Agricultural practices applied to soil cannot only change the bacterial community structure but can also have a large effect on specific bacterial groups. Kumar et al. (2023) reported that the conservation agriculture‐based production systems in the rice‐wheat‐greengram cropping system in the eastern Indo‐Gangetic Plains of India were dominated by Pseudomonadota, while the conventional tillage‐based scenarios were dominated by Acidobacteria and Chloroflexota. Wang et al. (2016) reported that the relative abundance of *Bacillus* and *Rhizobiales* increased in soil under conservation agriculture (no tillage) compared with that under 5‐year tillage. In this study, the agricultural practices applied had no significant effect on the bacterial community structure although some bacterial groups were affected strongly, for example, some groups belonging to Acidobacteriota (e.g., *11–24*, *RB41*, other Vicinamibactereaea), Chloroflexota (e.g., *UTCFX1*), and Actinomycetota (e.g., *Rubrobacter*).

Although the structure of the putative metabolic functions was not different between the unamended soils in this study, many putative metabolic functions were strongly affected by agricultural practices applied. Hariharan et al. (2017) reported that no‐tillage was functionally enriched for most nutrient cycles compared to the plough‐tillage system in a more than 50‐year‐old experiment in Ohio (USA). In this study, comparable results were found as some putative metabolic functions were strongly enriched in the minimum tilled soil with crop residue left on the soil surface and not incorporated (MITCW) compared to the conventional tilled soil (CTCC).

The effects of agricultural practices on the putative metabolic functions were different and much smaller than those on the bacterial groups. This would suggest that the relative abundance of bacterial groups is controlled more by random processes in the unamended soil than the relative abundance of putative metabolic functions. It must be stressed, however, that the metabolic functions reported here are ‘putative metabolic functions’ predicted from taxonomic data, which can sometimes underestimate gene frequencies (Toole et al., 2021) or perform weakly with environmental microbiomes (Sun et al., 2020). While this analysis provides a broad understanding of putative metabolic pathways and functions, additional investigations into microbial gene expression will be necessary to validate and further explore the role of bacteria and other microorganisms, for example, fungi, in the degradation of organic material.

### Young maize plant amended soil

The application of organic material, such as maize plants and their NDF fraction, has a profound effect on the soil bacterial community structure (Yue et al., 2023). The C substrate is mineralized mostly by heterotrophic bacteria, but also by fungi (Hernández‐Guzmán et al., 2022; Kim et al., 2021). The relative abundance of bacteria that degrade the organic material will increase while that of those that do not immediately participate in the mineralization will decrease. The prime organic material degraders are considered copiotrophs (Fierer et al., 2007; Koch, 2001) or R‐strategists (Pianka, 1970), while the relative abundance of those that do not participate in the initial degradation decreases and they are considered oligotrophs or K‐strategists (Wu et al., 2021). As such, the first is enriched in nutrient‐rich environments while the latter is in nutrient‐poor ones. How microorganisms respond to the application of the organic material will depend on the composition of the organic material applied, for example, C‐to‐N ratio and lignin content, soil characteristics, such as pH and salt content, climatical conditions, but also on agricultural practices (Cui et al., 2023). For instance, Arcand et al. (2016) reported that changes in the decomposer community composition were greater in soils originating from organic farming than from conventional management.

The application of organic material often affects alpha diversity (Sabir et al., 2021), but how will depend on the type of organic material applied and soil characteristics, for example, pH (Shu et al., 2022). For instance, Yue et al. (2023) reported that wheat straw and pig manure consistently decreased bacterial alpha diversity (Chao1 and Shannon index), while Cui et al. (2023) in a global meta‐analysis reported that organic amendments increased the bacterial diversity indices (Shannon and Chao1). In this study, the application of young maize plants did not affect the bacterial richness (Hill number at *q* = 0).

Acidobacteriota (Acidobacteria, Oren & Garrity, 2021) are mostly oligotrophic and enriched when the available organic material becomes more recalcitrant (Shen et al., 2023), while Actinomycetota has often been described as copiotrophic (Liu et al., 2023), but not always (Lin & Lin, 2022). In this study, the application of young maize plants increased the relative abundance of Actinomycetota, while that of Acidobacteriota decreased. Other bacterial phyla, such as Chloroflexota and Gemmatimonadota, also showed oligotrophic behaviour as reported by Lin and Lin (2022) and Li et al. (2023), but the decrease in relative abundance was less accentuated than that of Acidobacteriota. Pseudomonadota, and Bacteroidetes were found to have only copiotrophic strategies in a meta‐analysis study with a significant increase in response to organic amendments (Cui et al., 2023). In this study, Bacteroidota showed no copiotrophic behaviour and Pseudomonadota only on day 1.

The bacterial genera that were enriched by the application of young maize plants in this study are well‐known copiotrophs, that is, *Streptomyces* (e.g., Su et al., 2020) and *Nocardioides* (Guo et al., 2020). Guo et al. (2020) found that *Streptomyces* was the predominant utilizer of ^13^C derived from rice root residues within a 28‐day incubation, but also *Nocardioides*. Chiba et al. (2021) reported that *Nocardioides* were enriched during the early decomposition phases of the maize leaf litter‐derived C as in this study, but not *Streptomyces*. Although they reported that *Streptomyces* is a known plant‐degrading genus (Hernández‐Coronado et al., 1998), they stated the relative decrease in the relative abundance of *Streptomycetaceae* could be attributed to increasing bacterial competition for nutrient acquisition during litter decomposition. The relative abundance of *Kribbella*, nitrogen‐fixing bacteria (*K. flavida* https://www.genome.jp/pathway/kfl00910+M00530), also increased substantially in the young maize plant amended soil. *Kribbella* was enriched in the soil after afforestation with *Larix decidua* M., *Pinus sylvestris* L., *Quercus robur* L., and *Picea abies* L. (Borowik et al., 2022) and its relative abundance was larger traditional farming systems compared to organic farming (Khmelevtsova et al., 2022). It is difficult to be sure why this genus was enriched in the young maize plant amended soil, but its capacity to fix N_2_ might have favoured it when organic material was applied to the N‐depleted soil.

### NDF amended soil

(Hemi)cellulose is one of the most distributed organic molecules on earth and is an essential part of plant cell walls (Huang et al., 2021). Consequently, a wide range of microorganisms can degrade it, but when applied to soil only a limited number of bacteria participate in its initial degradation. In this study, the bacteria enriched by the application of the NDF fraction, mostly (hemi)cellulose and some lignin, were the same that were enriched when young maize plants were applied to the soil, for example, *Streptomyces*, *Nocardioides*, and *Kribbella*.

Members of *Rhodococcus* were enriched on days 3 and 7 by the application of NDF, but not by the application of maize. Kim et al. (2018) summarized the characteristics of *Rhodococcus* as ‘*a phylogenetically and catabolically diverse group with a versatile ability to degrade different natural and synthetic organic compounds as a result of a wide range of catabolic genes, which are believed to be obtained through frequent recombination events mediated by large catabolic plasmids*’. For instance, Dornau et al. (2020) reported that *Rhodococcus opacus* efficiently fermented the organic fraction of municipal solid waste fibre hydrolysate, that is, 72% of the maximum theoretical fermentation yield, that contained approximately 50% lignocellulose‐rich material, better than any other bacteria tested.

Although the amount of lignin was low in the young maize plants (2.5%) and NDF (6.3%) some of the bacteria most enriched by their application, for example, *Acinetobacter*, *Nocardioides*, and *Streptomyces*, were also found to be involved in the degradation of lignin, that is, they were capable of cleaving β‐O‐4 alkyl aryl ether which is the most abundant linkage within lignin (Oya et al., 2022). That would indicate that these groups were favoured by N‐organic poor material (NDF) and were ‘outcompeted’ by other groups when organic N richer material (maize plants) was applied to the soil.

### Urea‐amended soil

Application of inorganic fertilizer usually does not affect C mineralization and the bacterial community structure (e.g., Guo et al., 2019; Li et al., 2018), but not always (Hernández‐Guzmán et al., 2022). For instance, Li et al. (2018) reported that chemical fertilizer application alone did not alter the soil microbial communities, but straw in a wheat‐maize double cropping system in Northern China did. In a previous experiment with Vertisol soil from Mexico cultivated with maize and wheat in rotation, the application of 300 mg NH_4_^+^–N kg^−1^ soil increased the C mineralization and enriched many bacterial groups (Hernández‐Guzmán et al., 2022). The application of 300 mg NH_4_^+^–N kg^−1^ to the N‐depleted soil allowed the bacterial community to mineralize more organic material. It enriched members of *Pseudomonas*, *Flavisolibacter*, *Enterobacter*, and *Pseudoxanthomonas* in the first week and *Rheinheimera*, *Acinetobacter*, and *Achromobacter* between days 7 and 28. In this study, the application of urea had a limited effect on the relative abundance of bacterial groups and the putative metabolic functions. It can be hypothesized that although the soil was N depleted it was not so severe as in the Vertisol from Mexico.

## CONCLUSIONS

After 30 years, the soil organic C content was higher in the minimum tilled soil with crop rotation and residue retention (MITCW treatment) than in the conventional tilled with cotton monoculture and residue removal (CTCC). No other soil characteristic apart from the WHC was affected by agricultural practices. Application of NDF did not increase the soil mineral N content, but it did when young maize plants or urea were added to the soil. Bacterial richness was not affected by agricultural practices or application of young maize plants, its NDF fraction or urea. Although the bacterial community and the putative metabolic functional structure were not affected significantly by agricultural practices, many bacterial groups and specific putative metabolic functions were affected strongly. The application of young maize plants and the NDF fraction did change the bacterial community and putative metabolic functional structure, but not urea. Relative to the unamended soil, application of maize and NDF‐enriched bacterial groups, such as Actinomycetota, *Kribbella*, *Nocardioides*, and *Streptomyces* and a wide range of putative metabolic functions. In summary, the application of maize plants or NDF to an irrigated Vertisol with a history of differing cotton farming systems (tillage systems, crop rotations) changed bacterial functionality and its community structure, but not urea.

## AUTHOR CONTRIBUTIONS

**Luc Dendooven:** Conceptualization; writing – original draft; writing – review and editing; supervision; formal analysis; project administration. **Daniel Ramírez‐Villanueva:** Investigation; writing – review and editing; methodology. **Vanessa Romero‐Yahuitl:** Methodology; data curation. **Karla E. Zarco‐González:** Writing – review and editing; data curation; formal analysis. **Nilantha Hulugalle:** Methodology; writing – review and editing; resources. **Viliami Heimoana:** Methodology; writing – review and editing; resources. **Nele Verhulst:** Conceptualization; writing – review and editing; resources; project administration. **Bram Govaerts:** Conceptualization; writing – review and editing; resources. **Yendi E. Navarro‐Noya:** Conceptualization; methodology; data curation; supervision; resources; writing – original draft; writing – review and editing.

## CONFLICT OF INTEREST STATEMENT

The authors declare no conflicts of interest.

## Supporting information


**Figure S1.** Experimental design.

**Figure S2.** (a) The alpha diversity with Hill numbers at *q* = 0, *q* = 1, and *q* = 2 in soil cultivated with cotton (*Gossypium hirsutum* L.) monoculture (summer cotton‐winter, fallow‐summer cotton) conventional tillage (CTCC), minimum tillage of continuous cotton (MITCC), and minimum tillage cotton‐wheat (*Triticum aestivum* L.) rotation (summer cotton‐winter wheat‐summer and winter fallow‐summer cotton) (MITCW) left unamended (□) or amended with maize (*Zea mays* L.) (○), its neutral detergent fibre (NDF) (●) or urea (■) incubated aerobically at 22 ± 2°°C for 28 days, and (b) the beta diversity with beta.jtu: dist dissimilarity matrix accounting for spatial turnover, measured as the turnover‐fraction of Jaccard pair‐wise dissimilarity, i.e., indicates 1‐for‐1 species substitutions, beta.jne: dist object, dissimilarity matrix accounting for nestedness‐resultant dissimilarity, measured as the nestedness‐fraction of Jaccard pair‐wise dissimilarity, i.e., indicates species gain or loss without substitution, beta.jac: dist object, dissimilarity matrix accounting for beta diversity, measured as Jaccard pair‐wise dissimilarity (a monotonic transformation of beta diversity), i.e., the full Jaccard index with values closer to 1 indicate greater dissimilarity (Baselga & Orme, 2012) for the maize (mai), neutral detergent fibre (NDF) and urea‐amended soil (ure) versus the unamended soil.

**Figure S3.** Principal component analysis (PCA) with (a) the different bacterial phyla, (b) all bacterial groups assigned up to the taxonomic level of genus, (c) the amplicon sequence variants (ASVs) and (d) the putative metabolic functions in the unamended CTCC (□), MITCC (●) and MITWC (▲) soil incubated aerobically at 22 ± 2°C for 28 days. The values in the symbols are the number of days the soil was incubated aerobically and the explanation of the abbreviations of the agricultural practices can be found in the legend in Figure S2.

**Figure S4.** Changes in the relative abundance (%) of some selected bacterial groups assigned up to the taxonomic level of the genus in the unamended CTCC (□), MITCC (■), and MITCC (■) soil incubated aerobically at 22 ± 2°C for 28 days. The explanation of the abbreviations of the agricultural practices can be found in the legend in Figure S2.

**Figure S5.** Changes in the relative abundance (%) of some selected putative metabolic functions in the unamended CTCC (□), MITCC (■), and MITCC (■) soil incubated aerobically at 22 ± 2°C for 28 days. The explanation of the abbreviations of the agricultural practices can be found in the legend in Figure S2.

**Figure S6.** Changes in the relative abundance (%) of the most abundant bacterial phyla in soil (mean of the three soils with different agricultural practices) left unamended (□) or amended with maize (*Zea mays* L.) (■), its neutral detergent fibre (NDF) fraction (■) or urea (■) incubated aerobically at 22 ± 2°C for 28 days.

**Figure S7.** (a) Volcano plot comparing the relative abundance of all groups assigned up to the taxonomic level of the genus in unamended CTCC, MITCC, and MITCW soils compared to the same soils (a) amended with young maize plants (*Zea mays* L.), (b) their neutral detergent fibre fraction or (c) urea incubated aerobically at 22 ± 2°C for 28 days. The expected *p‐value* of the Kruskal–Wallis test is given on the *y*‐axis and the effect size is on the *x*‐axis (Gloor et al., 2017). The effect size, which is defined as the difference between groups divided by the maximum dispersion within group A or B, was calculated with the ALDEx2 package using the aldex.ttest argument. A positive value indicates that the relative abundance of the microbial group was higher in the unamended soil compared to soil amended with young maize plants, their neutral detergent fibre (NDF) fraction or urea while a negative value indicates the opposite. Vertical lines indicate large (≤ −0.8, ≥ 0.8) and very large effect sizes (≤ −1.4, ≥ 1.4) (Kim, 2015). The explanation of the abbreviations of the agricultural practices can be found in the legend in Figure S2.

**Figure S8.** (a) Volcano plot comparing the relative abundance of putative metabolic functions in the unamended CTCC, MITCC, and MITCW soils compared to the same soils (a) amended with young maize plants (*Zea mays* L.), (b) their neutral detergent fibre fraction or (c) urea incubated aerobically at 22 ± 2°C for 28 days. The explanation of the abbreviations of the agricultural practices can be found in the legend in Figure S2 and how the effect size was calculated in Figure S7.

**Figure S9.** Changes in the relative abundance (%) of the most abundant putative metabolic functions in soil (mean of three soils with the different agricultural practices) left unamended (□) or amended with young maize plants (*Zea mays* L.) (■), their neutral detergent fibre (NDF) fraction (■) or urea (■) incubated aerobically at 22 ± 2°C for 28 days.

**Figure S10.** Principal component analysis (PCA) with (a) all the bacterial groups assigned up to the taxonomic level of genus and (b) the putative metabolic functions in the maize plant amended CTCC (■), MITCC (●), and MITCW (▲) soils, the NDF‐amended CTCC (□), MITCC (○), and MITCW (△) soils, and the urea‐amended CTCC (■), MITCC (●), and MITCW (▲) soils. The values in the symbols are the number of days the soil was incubated aerobically. The explanation of the abbreviations of the agricultural practices used in the figure can be found in the legend of Figure S2.

**Table S1.** Summary of irrigated treatments from the Australian Cotton Research Institute (ACRI) included in this study started in 1985.

**Table S2.** Characteristics of organic material used in the aerobic incubation experiment (Ramirez‐Villanueva et al., 2015).

**Table S3.** Effect of agricultural practice (soil cultivated with cotton (*Gossypium hirsutum* L.) monoculture (summer cotton‐winter, fallow‐summer cotton) conventional tillage (CTCC), minimum tillage of continuous cotton (MITCC), and minimum tillage cotton‐wheat (*Triticum aestivum* L.) rotation (summer cotton‐winter wheat‐summer and winter fallow‐summer cotton) (MITCW)) in the unamended soil or soil amended with young maize plants, their neutral detergent fibre (NDF) fraction or urea, the effect of treatment (unamended soil or soil amended with young maize plants, their NDF fraction or urea) in the CTCC, MITCC, or MITCW soil, and the effect of time (day 0, 1, 3, 7, 14, and 28 days) on the Hill numbers at *q* = 0, 1 and 2.

**Table S4.** Effect of agricultural practices on the relative abundance of bacterial groups assigned up to the taxonomic level of genus. Only bacterial groups with a large effect size ≤ −0.8 and ≥0.8 are given (Kim, 2015). The effect size, which is defined as the difference between groups divided by the maximum dispersion within group X or Y, was calculated with the aldex.ttest argument (ALDEx2 (version, 1.18), Gloor et al. (2020)).

**Table S5.** Putative metabolic functions with an effect size (≤ −1.4 and ≥1.4) when comparing the relative abundance of putative metabolic functions in soil cultivated with cotton (*Gossypium hirsutum* L.) monoculture (summer cotton‐winter, fallow‐summer cotton) conventional tillage (CTCC), minimum tillage of continuous cotton (MITCC), and minimum tillage cotton‐wheat (*Triticum aestivum* L.) rotation (summer cotton‐winter wheat‐summer and winter fallow‐summer cotton) (MITCW) after 1, 3, 7, 14, or 28 days aerobic incubation.

**Table S6.** Effect of application of young maize plants, the neutral detergent fibre (NDF) fraction or urea on the relative abundance of bacterial groups assigned up to the taxonomic level of genus compared their relative abundance in the unamended soils. Only bacterial groups that were significantly affected by treatment are given (*p* < 0.05). The effect size, which is defined as the difference between groups divided by the maximum dispersion within group X or Y, was calculated with the aldex.ttest argument (ALDEx2 (version, 1.18), Gloor et al. (2020)).

**Table S7.** The number of putative metabolic functions with an effect size (≤ −0.8 and ≥0.8) when comparing the relative abundance of putative metabolic functions separately in soil cultivated with cotton (*Gossypium hirsutum* L.) monoculture (summer cotton‐winter, fallow‐summer cotton) conventional tillage (CTCC), minimum tillage of continuous cotton (MITCC), and minimum tillage cotton‐wheat (*Triticum aestivum* L.) rotation (summer cotton‐winter wheat‐summer and winter fallow‐summer cotton) (MITCW) with the same soils amended with maize or its neutral detergent fibre (NDF) fraction after 1, 3, 7, 14, or 28 days of an aerobic incubation.

**Table S8.** The number of putative metabolic functions (Function) and bacterial groups assigned up to the taxonomic level of genus (Genera) with an effect size (≤ −0.8 and ≥0.8) when comparing the relative abundance of putative metabolic functions and bacterial genera in soil cultivated with cotton (*Gossypium hirsutum* L.) monoculture (summer cotton‐winter, fallow‐summer cotton) conventional tillage (CTCC), minimum tillage of continuous cotton (MITCC), and minimum tillage cotton‐wheat (*Triticum aestivum* L.) rotation (summer cotton‐winter wheat‐summer and winter fallow‐summer cotton) (MITCW) at the onset of the experiment and after 1, 3, 7, 14 or 28 days of aerobic incubation.

## Data Availability

The 16S rRNA gene sequence datasets and the sample metadata are available in the NCBI Sequence Read Archive (SRA) under the BioProject PRJNA1057489.
