# Supplementary material for: Young maize plants impact the bacterial community in Australian cotton‐sown vertisol more than agricultural practices
Source: Environ Microbiol Rep. 2025 Apr 30;17(3):e13322. doi: 10.1111/1758-2229.13322 (PMC12041893; doi:10.1111/1758-2229.13322)
Supplement: Supplementary file 1 — Figure S1. Experimental design. [file EMI4-17-e13322-s002.pdf]

Application of maize plants, its neutral detergent fibre (NDF) fraction and urea to an arable soil with different agricultural management practices

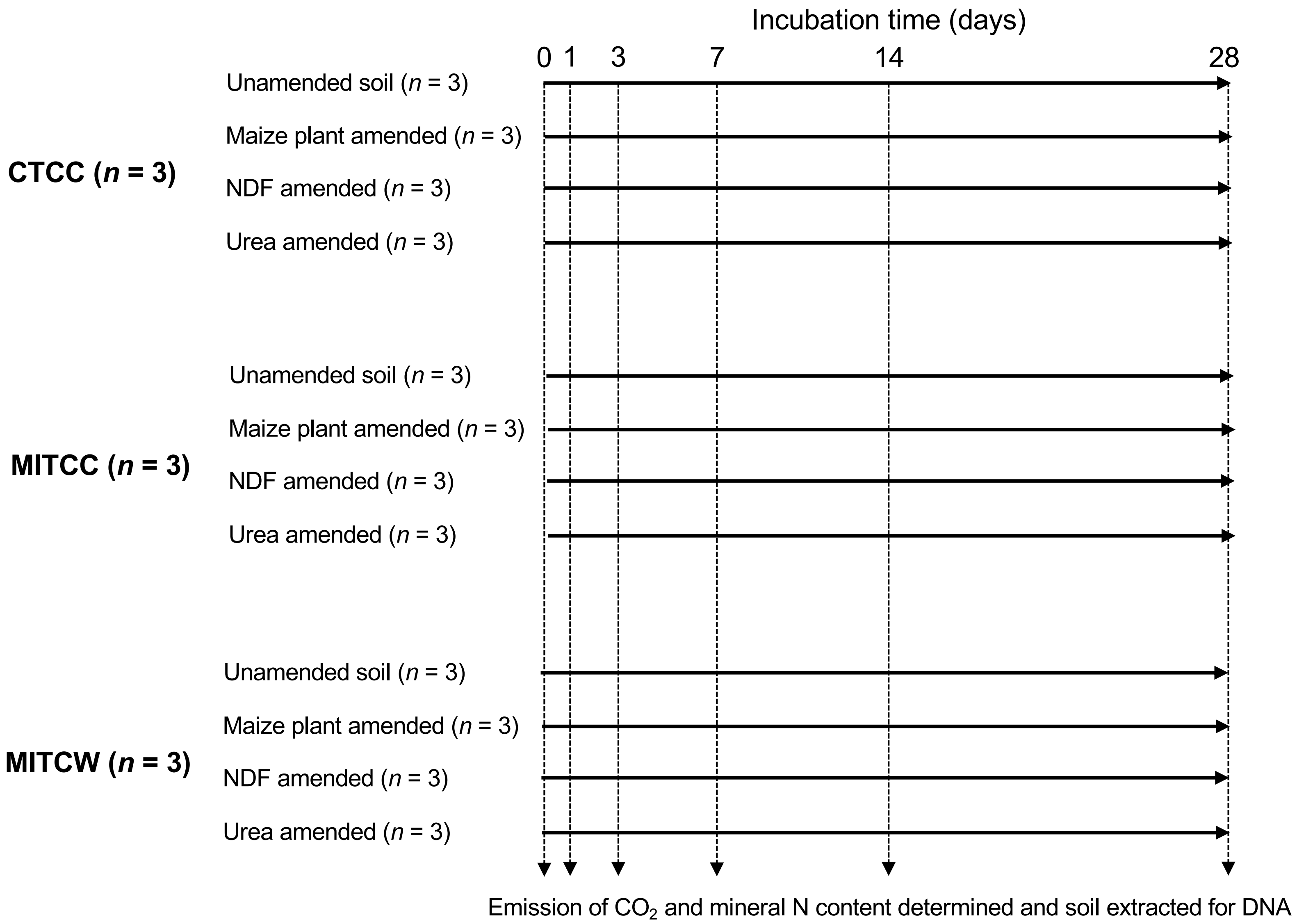

CTCC: Conventional tilled cotton (*Gossypium hirsutum* L.) monoculture crop residue incorporated
MITCC: Permanent raised beds, continuous cotton, crop residue incorporated
MITCW: Permanent raised beds, cotton - wheat rotation (*Triticum aestivum* L.), wheat stubble retained
