# Supplementary figures and images for: Young maize plants impact the bacterial community in Australian cotton‐sown vertisol more than agricultural practices
Source: Environ Microbiol Rep. 2025 Apr 30;17(3):e13322. doi: 10.1111/1758-2229.13322 (PMC12041893; doi:10.1111/1758-2229.13322)

## a) Alpha diversity analysis

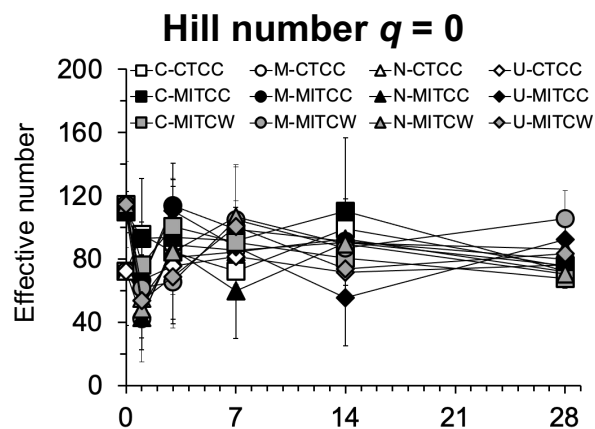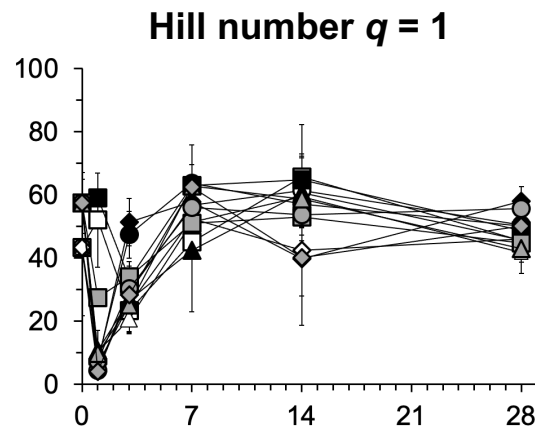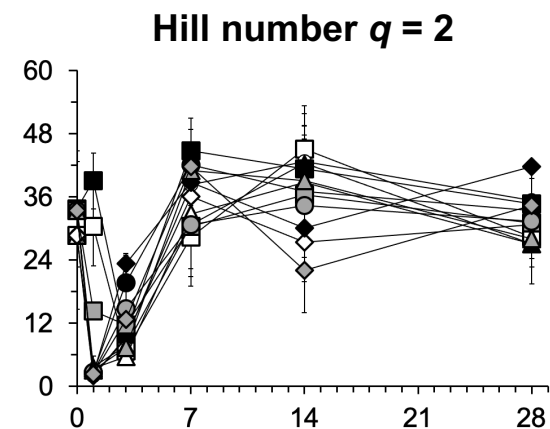

## b) Beta diversity analysis

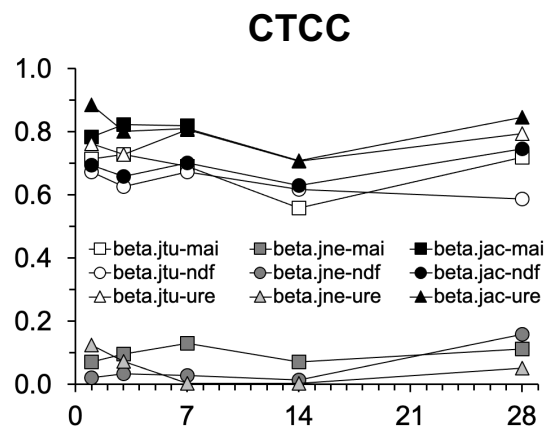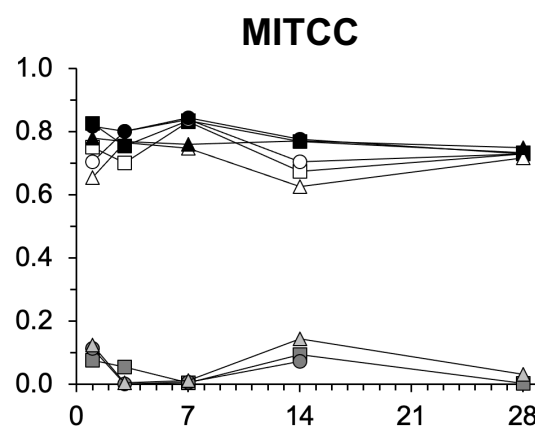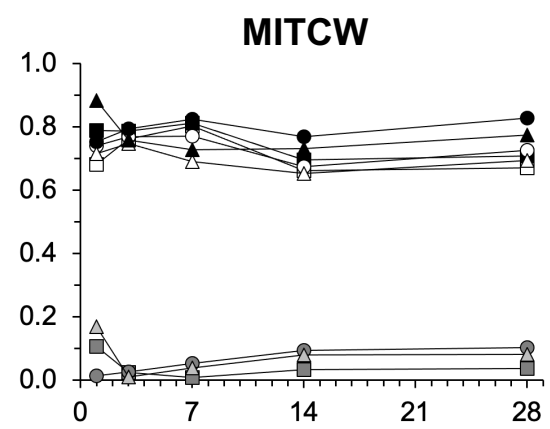

Time (days)

Supplement: Supplementary file 2 — Figure S2. (a) The alpha diversity with Hill numbers at q = 0, q = 1, and q = 2 in soil cultivated with cotton (Gossypium hirsutum L.) monoculture (summer cotton‐winter, fallow‐summer cotton) conventional tillage (CTCC), minimum tillage of continuous cotton (MITCC), and minimum tillage cotton‐wheat (Triticum aestivum L.) rotation (summer cotton‐winter wheat‐summer and winter fallow‐summer cotton) (MITCW) left unamended (□) or amended with maize (Zea mays L.) (○), its neutral detergent fibre (NDF) (●) or urea (■) incubated aerobically at 22 ± 2°°C for 28 days, and (b) the beta diversity with beta.jtu: dist dissimilarity matrix accounting for spatial turnover, measured as the turnover‐fraction of Jaccard pair‐wise dissimilarity, i.e., indicates 1‐for‐1 species substitutions, beta.jne: dist object, dissimilarity matrix accounting for nestedness‐resultant dissimilarity, measured as the nestedness‐fraction of Jaccard pair‐wise dissimilarity, i.e., indicates species gain or loss without substitution, beta.jac: dist object, dissimilarity matrix accounting for beta diversity, measured as Jaccard pair‐wise dissimilarity (a monotonic transformation of beta diversity), i.e., the full Jaccard index with values closer to 1 indicate greater dissimilarity (Baselga & Orme, 2012) for the maize (mai), neutral detergent fibre (NDF) and urea‐amended soil (ure) versus the unamended soil. [file EMI4-17-e13322-s003.pdf]

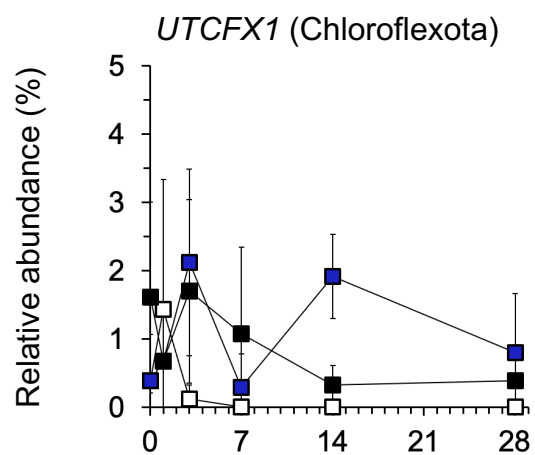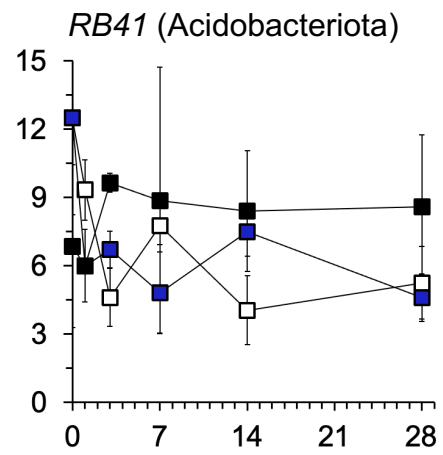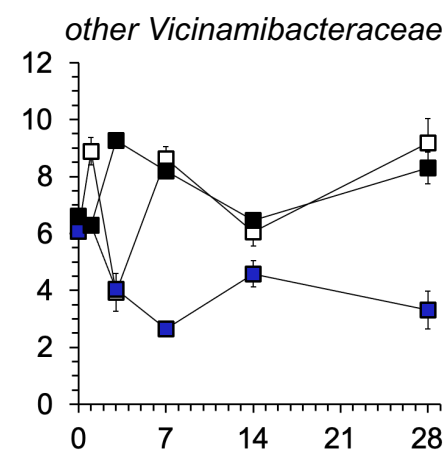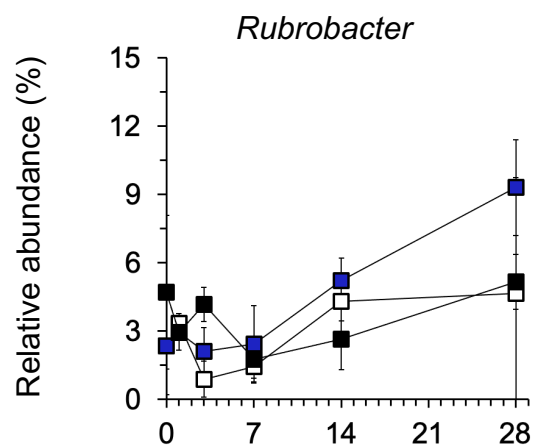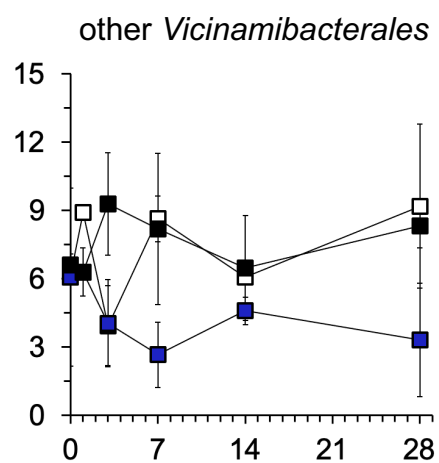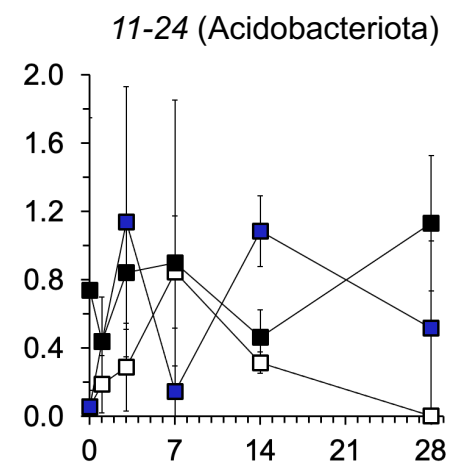

Time (days)

Supplement: Supplementary file 4 — Figure S4. Changes in the relative abundance (%) of some selected bacterial groups assigned up to the taxonomic level of the genus in the unamended CTCC (□), MITCC (■), and MITCC (■) soil incubated aerobically at 22 ± 2°C for 28 days. The explanation of the abbreviations of the agricultural practices can be found in the legend in Figure S2. [file EMI4-17-e13322-s015.pdf]

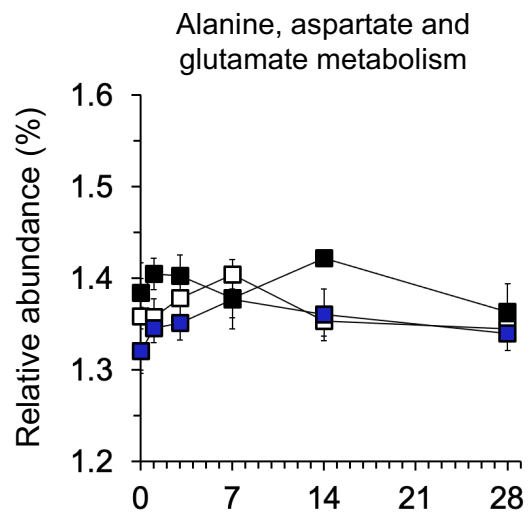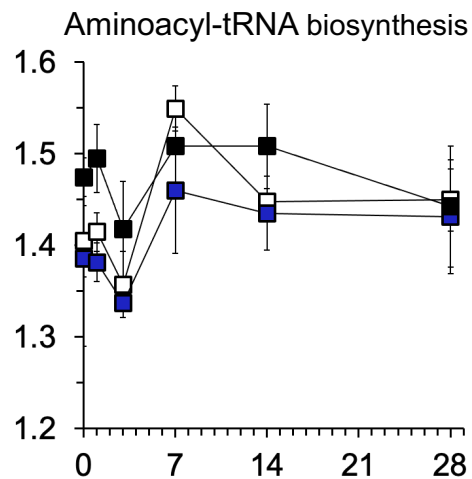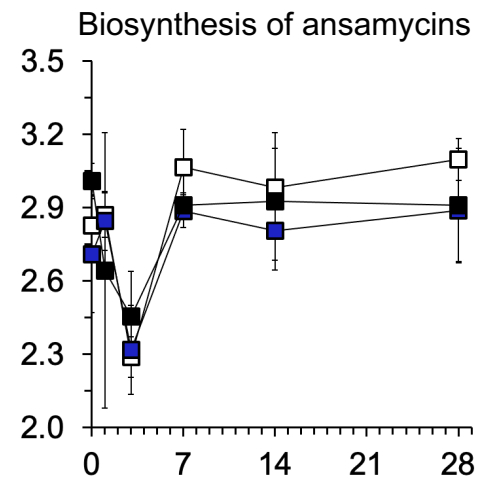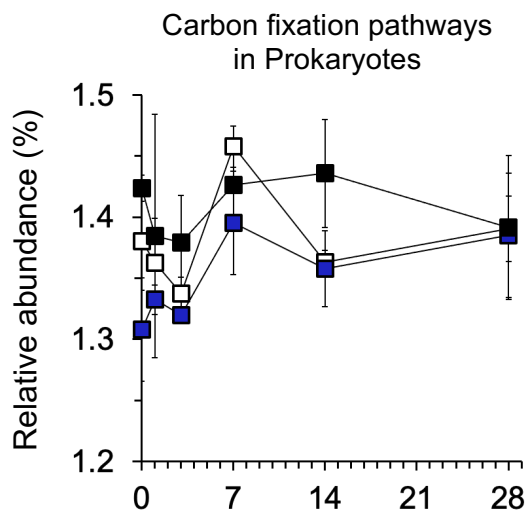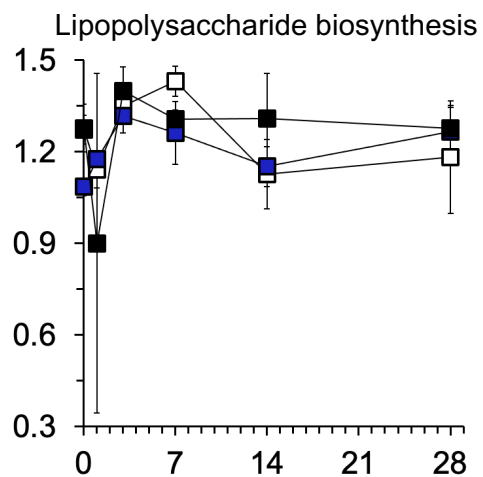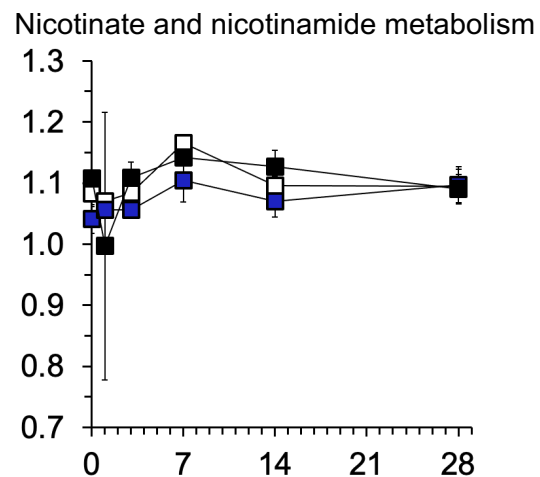

Time (days)

Supplement: Supplementary file 5 — Figure S5. Changes in the relative abundance (%) of some selected putative metabolic functions in the unamended CTCC (□), MITCC (■), and MITCC (■) soil incubated aerobically at 22 ± 2°C for 28 days. The explanation of the abbreviations of the agricultural practices can be found in the legend in Figure S2. [file EMI4-17-e13322-s005.pdf]

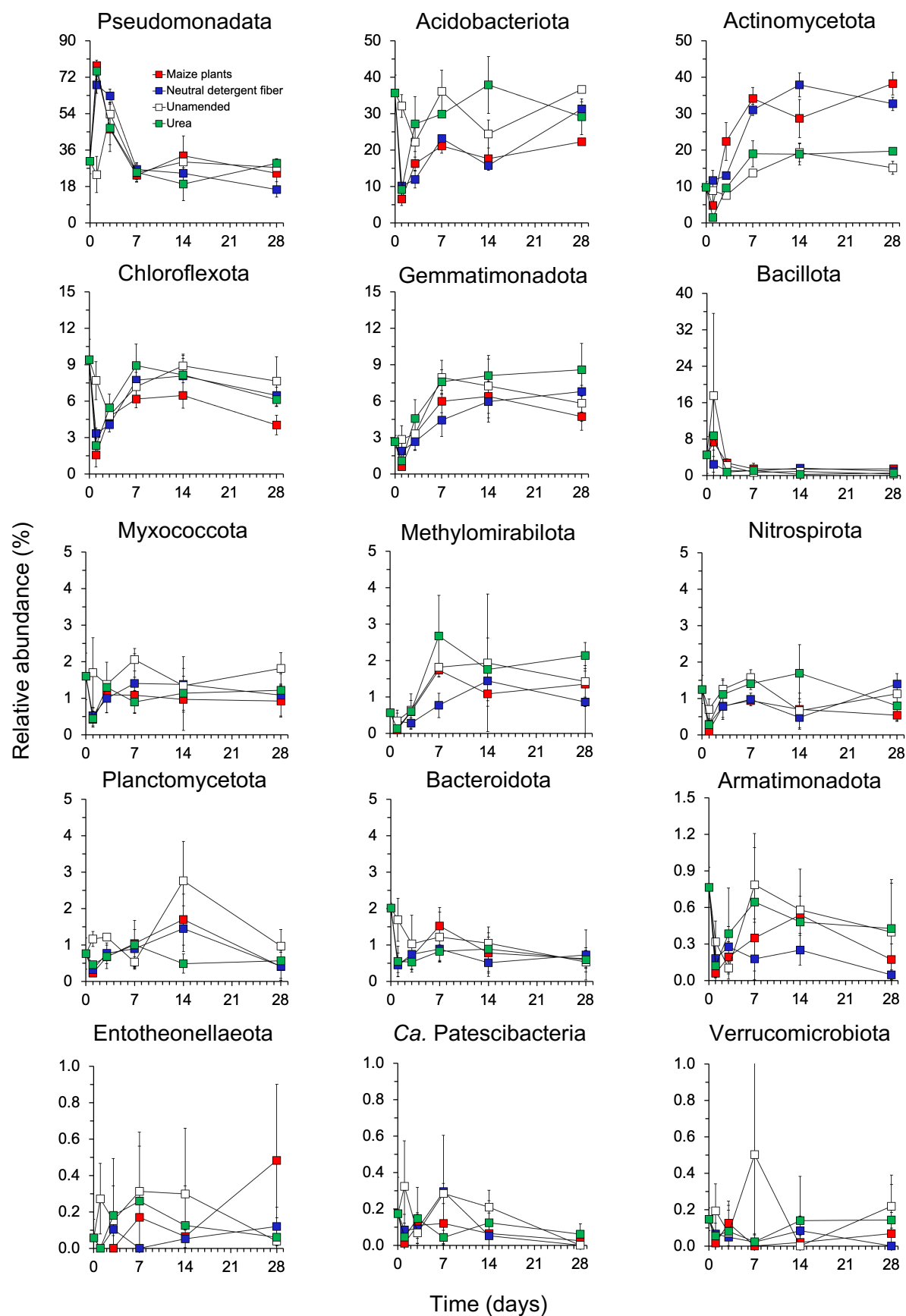

Supplement: Supplementary file 6 — Figure S6. Changes in the relative abundance (%) of the most abundant bacterial phyla in soil (mean of the three soils with different agricultural practices) left unamended (□) or amended with maize (Zea mays L.) (■), its neutral detergent fibre (NDF) fraction (■) or urea (■) incubated aerobically at 22 ± 2°C for 28 days. [file EMI4-17-e13322-s008.pdf]

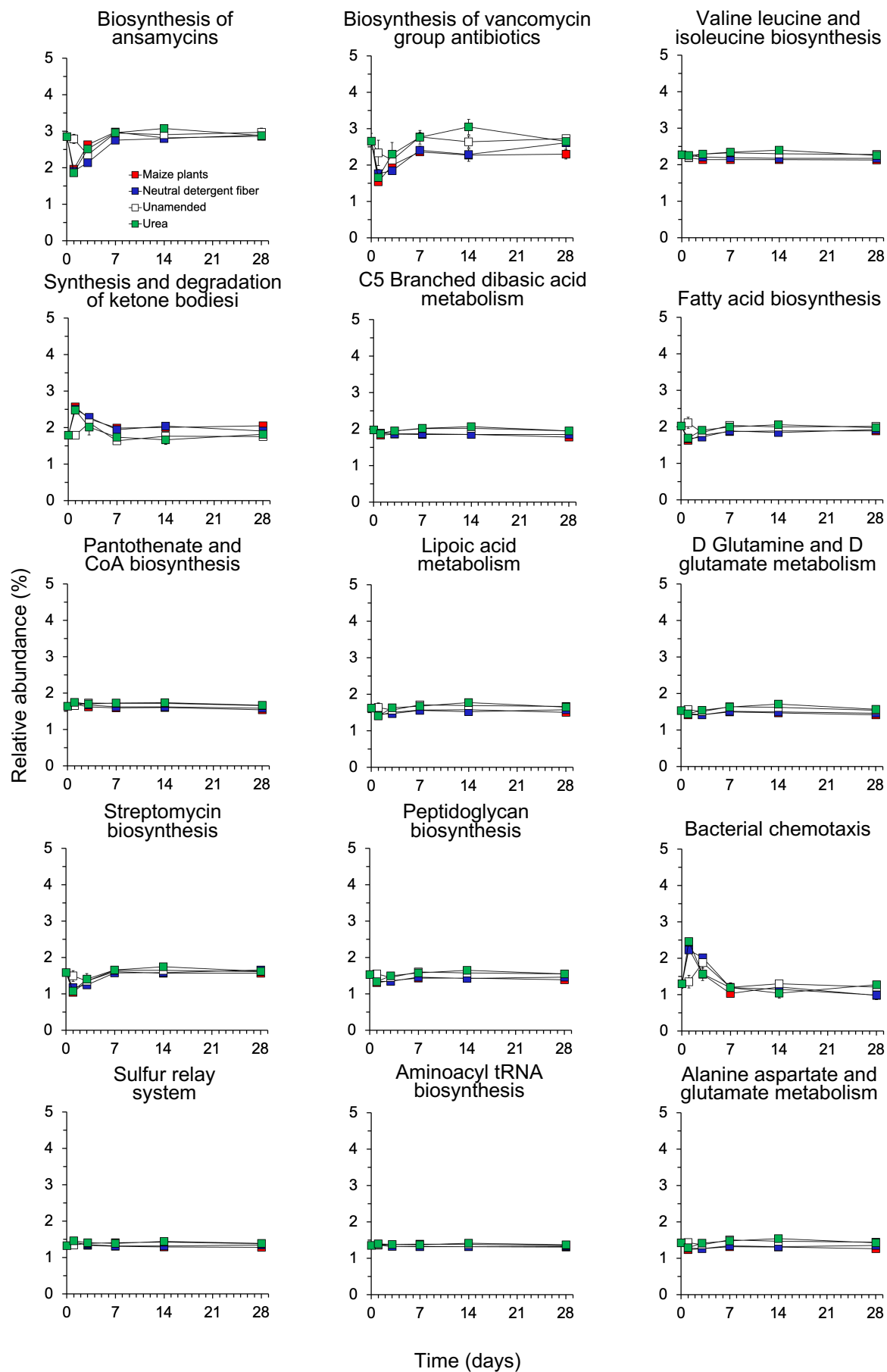

Supplement: Supplementary file 9 — Figure S9. Changes in the relative abundance (%) of the most abundant putative metabolic functions in soil (mean of three soils with the different agricultural practices) left unamended (□) or amended with young maize plants (Zea mays L.) (■), their neutral detergent fibre (NDF) fraction (■) or urea (■) incubated aerobically at 22 ± 2°C for 28 days. [file EMI4-17-e13322-s001.pdf]

## a) Bacterial genera

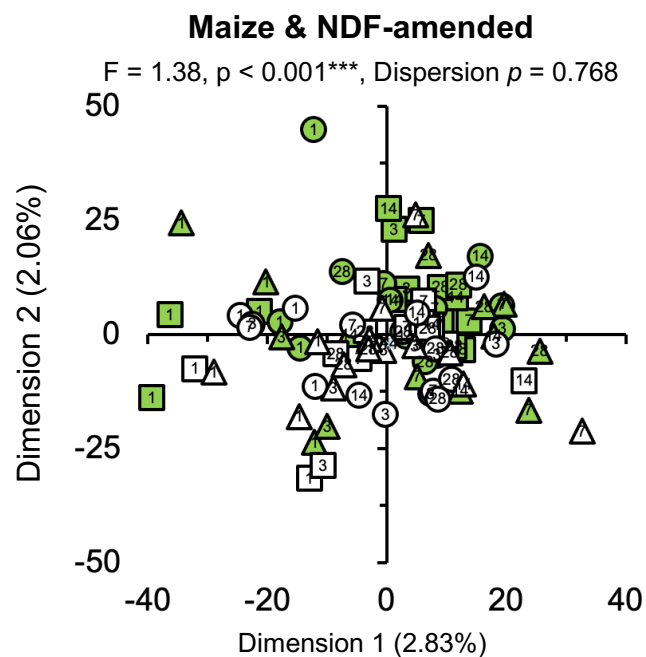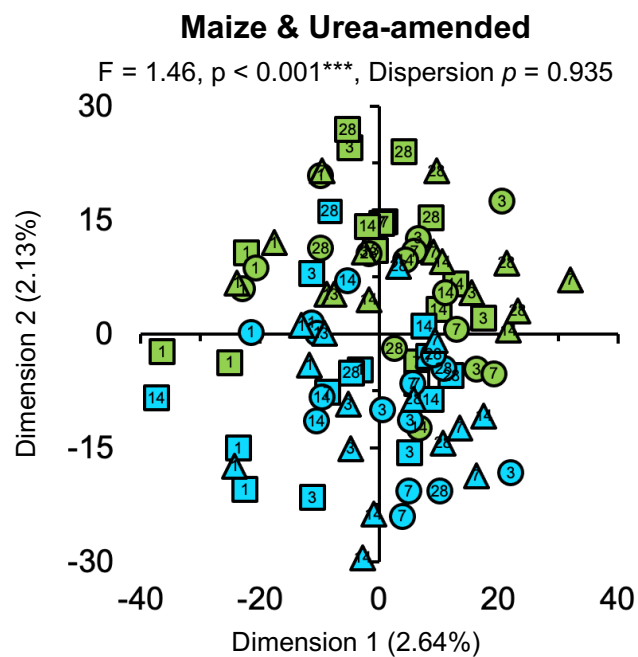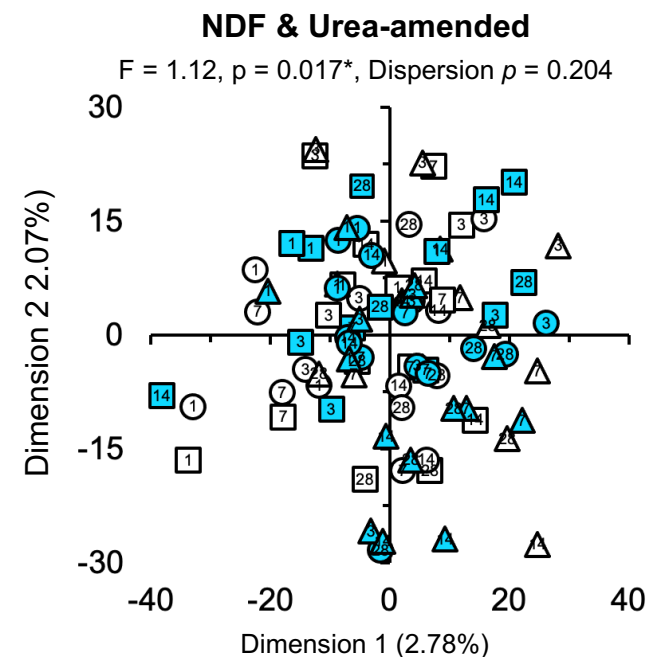

## b) Putative metabolic functions

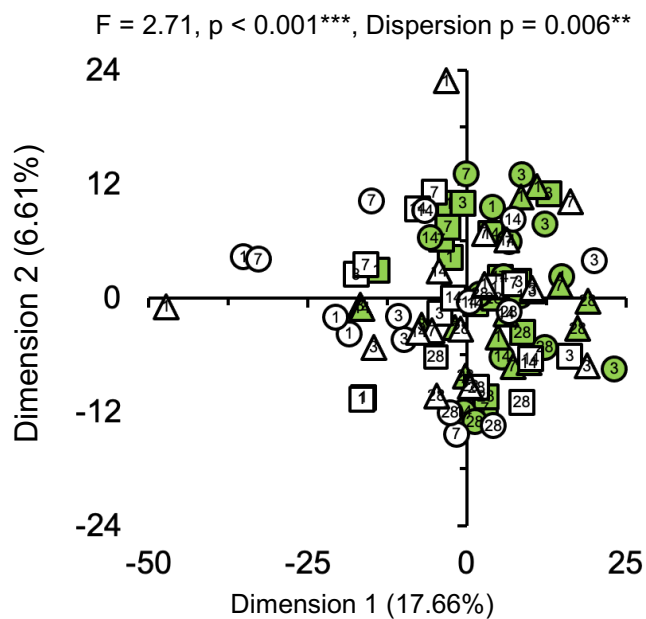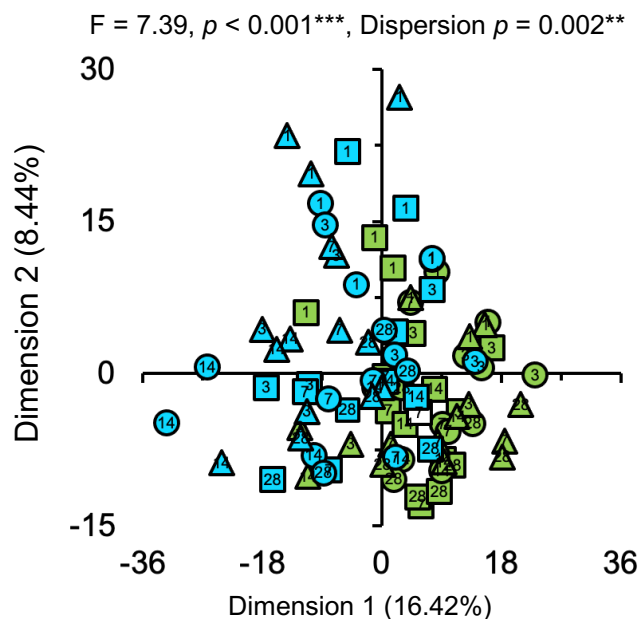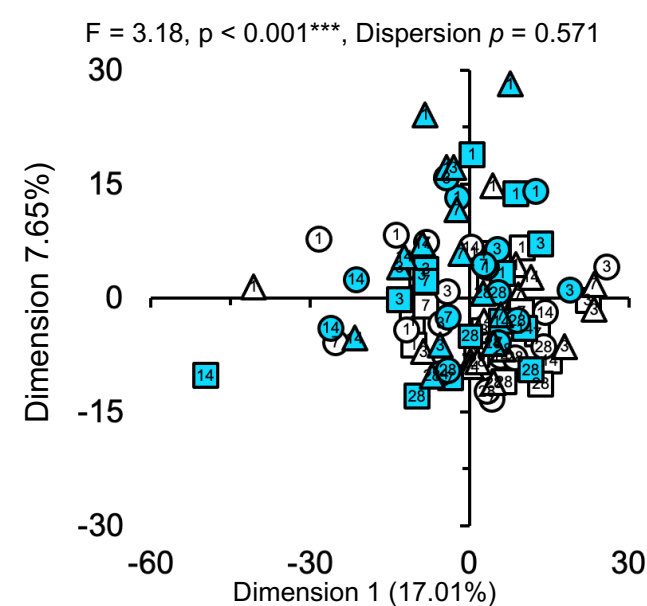

Supplement: Supplementary file 10 — Figure S10. Principal component analysis (PCA) with (a) all the bacterial groups assigned up to the taxonomic level of genus and (b) the putative metabolic functions in the maize plant amended CTCC (■), MITCC (●), and MITCW (▲) soils, the NDF‐amended CTCC (□), MITCC (○), and MITCW (△) soils, and the urea‐amended CTCC (■), MITCC (●), and MITCW (▲) soils. The values in the symbols are the number of days the soil was incubated aerobically. The explanation of the abbreviations of the agricultural practices used in the figure can be found in the legend of Figure S2. [file EMI4-17-e13322-s017.pdf]
