# Supplementary material for: Young maize plants impact the bacterial community in Australian cotton‐sown vertisol more than agricultural practices
Source: Environ Microbiol Rep. 2025 Apr 30;17(3):e13322. doi: 10.1111/1758-2229.13322 (PMC12041893; doi:10.1111/1758-2229.13322)
Supplement: Supplementary file 3 — Figure S3. Principal component analysis (PCA) with (a) the different bacterial phyla, (b) all bacterial groups assigned up to the taxonomic level of genus, (c) the amplicon sequence variants (ASVs) and (d) the putative metabolic functions in the unamended CTCC (□), MITCC (●) and MITWC (▲) soil incubated aerobically at 22 ± 2°C for 28 days. The values in the symbols are the number of days the soil was incubated aerobically and the explanation of the abbreviations of the agricultural practices can be found in the legend in Figure S2. [file EMI4-17-e13322-s010.pdf]

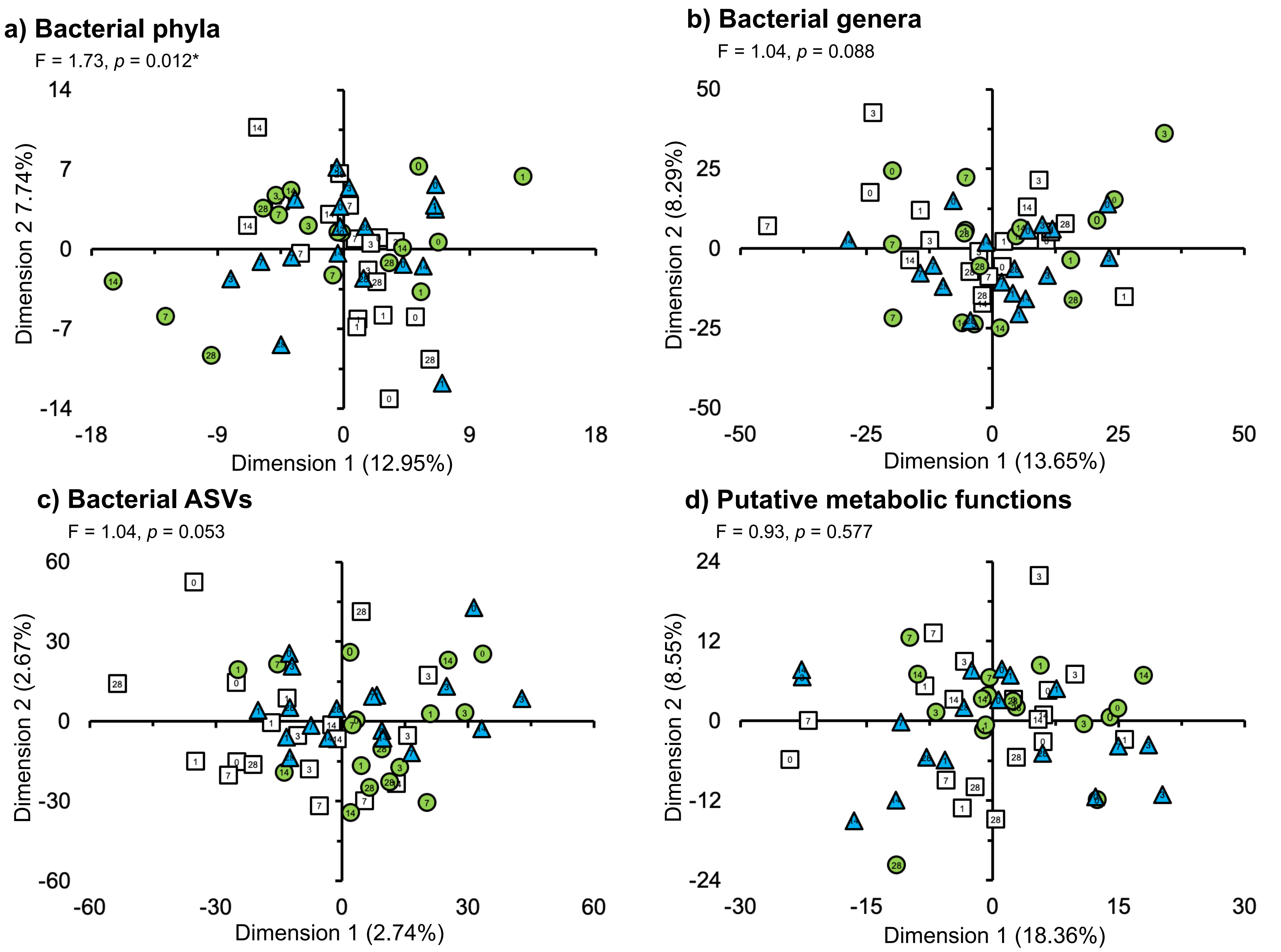
a) Bacterial phyla
F = 1.73, p = 0.012*
Dimension 1 (12.95%)
Dimension 2 7.74%)
b) Bacterial genera
F = 1.04, p = 0.088
Dimension 1 (13.65%)
Dimension 2 (8.29%)
c) Bacterial ASVs
F = 1.04, p = 0.053
Dimension 1 (2.74%)
Dimension 2 (2.67%)
d) Putative metabolic functions
F = 0.93, p = 0.577
Dimension 1 (18.36%)
Dimension 2 (8.55%)
