# Supplementary material for: Young maize plants impact the bacterial community in Australian cotton‐sown vertisol more than agricultural practices
Source: Environ Microbiol Rep. 2025 Apr 30;17(3):e13322. doi: 10.1111/1758-2229.13322 (PMC12041893; doi:10.1111/1758-2229.13322)

Bacterial groups assigned up to the taxonomic level of genus

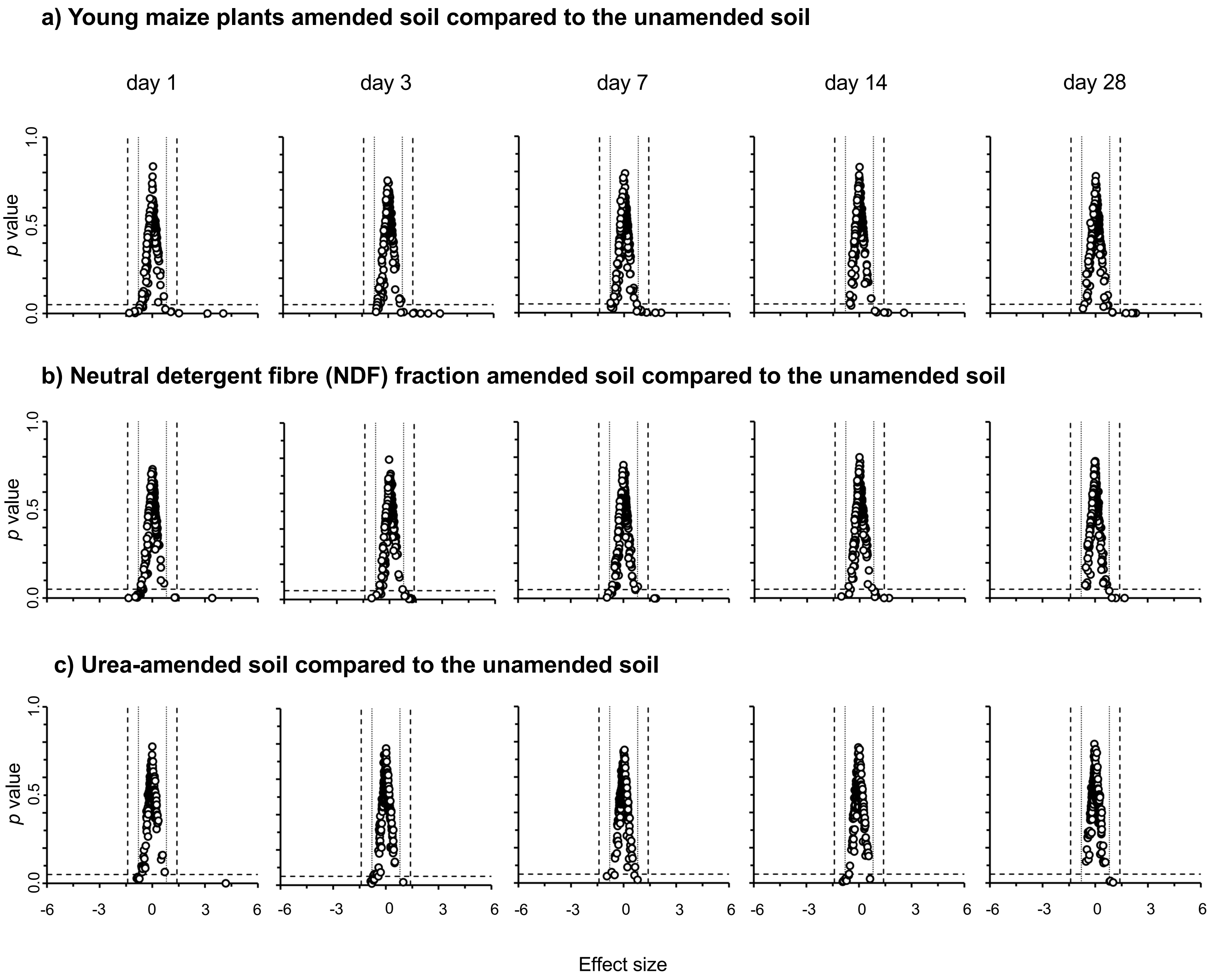

Supplement: Supplementary file 7 — Figure S7. (a) Volcano plot comparing the relative abundance of all groups assigned up to the taxonomic level of the genus in unamended CTCC, MITCC, and MITCW soils compared to the same soils (a) amended with young maize plants (Zea mays L.), (b) their neutral detergent fibre fraction or (c) urea incubated aerobically at 22 ± 2°C for 28 days. The expected p‐value of the Kruskal–Wallis test is given on the y‐axis and the effect size is on the x‐axis (Gloor et al., 2017). The effect size, which is defined as the difference between groups divided by the maximum dispersion within group A or B, was calculated with the ALDEx2 package using the aldex.ttest argument. A positive value indicates that the relative abundance of the microbial group was higher in the unamended soil compared to soil amended with young maize plants, their neutral detergent fibre (NDF) fraction or urea while a negative value indicates the opposite. Vertical lines indicate large (≤ −0.8, ≥ 0.8) and very large effect sizes (≤ −1.4, ≥ 1.4) (Kim, 2015). The explanation of the abbreviations of the agricultural practices can be found in the legend in Figure S2. [file EMI4-17-e13322-s013.pdf]
