# Supplementary material for: Young maize plants impact the bacterial community in Australian cotton‐sown vertisol more than agricultural practices
Source: Environ Microbiol Rep. 2025 Apr 30;17(3):e13322. doi: 10.1111/1758-2229.13322 (PMC12041893; doi:10.1111/1758-2229.13322)
Supplement: Supplementary file 8 — Figure S8. (a) Volcano plot comparing the relative abundance of putative metabolic functions in the unamended CTCC, MITCC, and MITCW soils compared to the same soils (a) amended with young maize plants (Zea mays L.), (b) their neutral detergent fibre fraction or (c) urea incubated aerobically at 22 ± 2°C for 28 days. The explanation of the abbreviations of the agricultural practices can be found in the legend in Figure S2 and how the effect size was calculated in Figure S7. [file EMI4-17-e13322-s009.pdf]

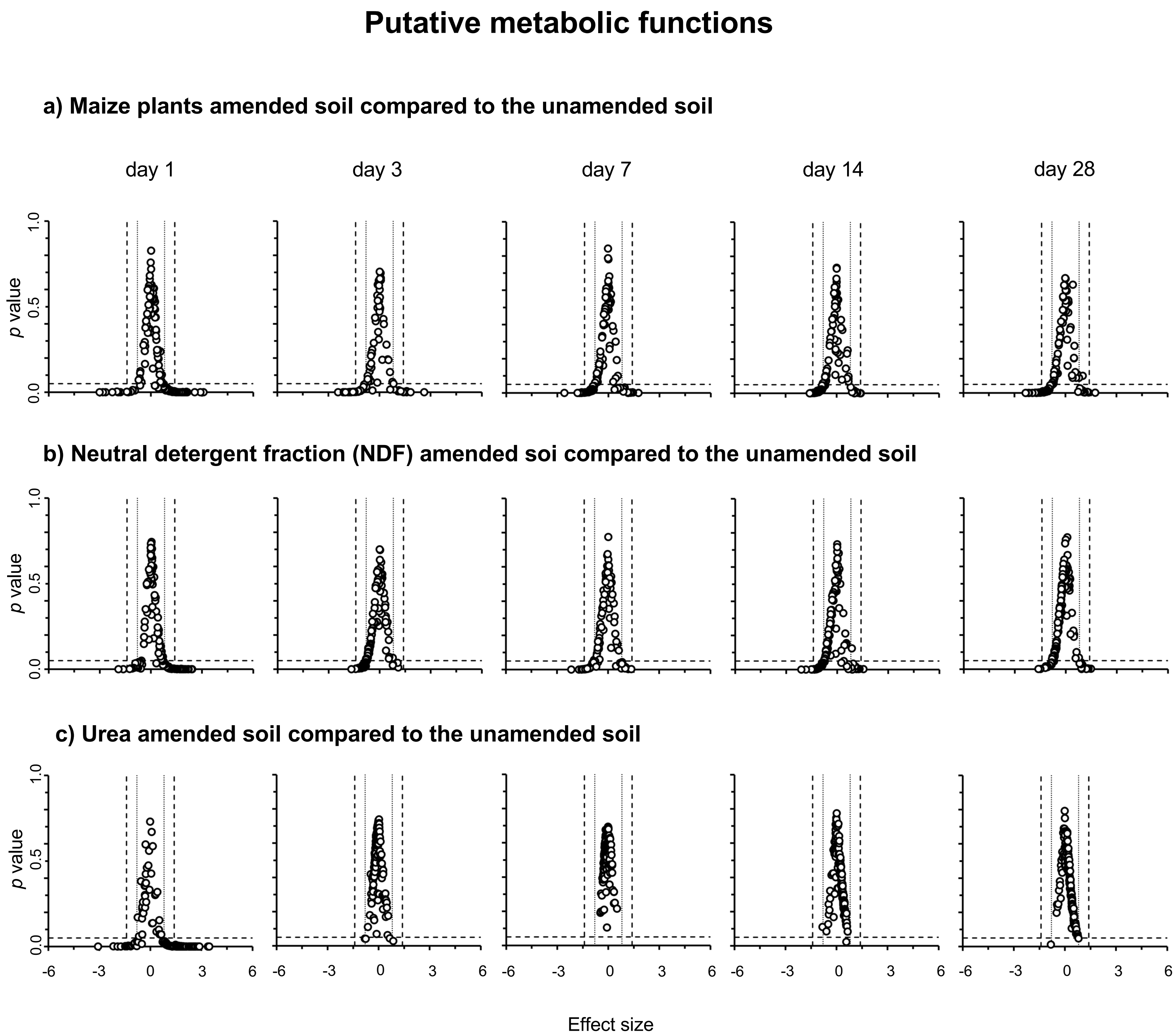

Putative metabolic functions
a) Maize plants amended soil compared to the unamended soil
b) Neutral detergent fraction (NDF) amended soi compared to the unamended soil
c) Urea amended soil compared to the unamended soil
day 1
day 3
day 7
day 14
day 28
p value
0.0
0.5
1.0
-6
-3
0
3
6
Effect size
