# Supplementary material for: Young maize plants impact the bacterial community in Australian cotton‐sown vertisol more than agricultural practices
Source: Environ Microbiol Rep. 2025 Apr 30;17(3):e13322. doi: 10.1111/1758-2229.13322 (PMC12041893; doi:10.1111/1758-2229.13322)
Supplement: Supplementary file 11 — Table S1. Summary of irrigated treatments from the Australian Cotton Research Institute (ACRI) included in this study started in 1985. [file EMI4-17-e13322-s014.docx]

| Table S1. Summary of irrigated treatments from the Australian Cotton Research Institute (ACRI) included in this study started in 1985. | | | |
| --- | --- | --- | --- |
|  | CTCC | MITCC | MITCW |
| Crop rotation | Cotton (*Gossypium hirsutum* L.)  (summer cotton-winter fallow-summer cotton) | Cotton (*Gossypium hirsutum* L.)  (summer cotton-winter fallow-summer cotton) | Cotton-Wheat (*Triticum aestivum* L.) rotation  (summer cotton-winter wheat-summer and winter fallow-summer cotton) |
| Residue | Incorporation of cotton stalks | Incorporation of cotton stalks | Until 1999 wheat stubble was incorporated into the soil before planting of conventional cotton. From then on Roundup-Ready cotton was planted retaining the wheat stubble |
| Tillage | Conventional tillage.  Slashing of cotton plants after harvest by disc-ploughing, chisel ploughing to 0.3 m followed by 1-m b construction to a height of 0.15 m | Permanent raised beds.  Slashing of cotton plants after harvest, followed by root cutting, and bed renovation with a disc-hiller | Permanent raised beds.  Direct seeding with no-tillage |
