# Supplementary material for: Young maize plants impact the bacterial community in Australian cotton‐sown vertisol more than agricultural practices
Source: Environ Microbiol Rep. 2025 Apr 30;17(3):e13322. doi: 10.1111/1758-2229.13322 (PMC12041893; doi:10.1111/1758-2229.13322)
Supplement: Supplementary file 12 — Table S2. Characteristics of organic material used in the aerobic incubation experiment (Ramirez‐Villanueva et al., 2015). [file EMI4-17-e13322-s018.doc]

Table S2. Characteristics of organic material used in the aerobic incubation experiment (Ramirez-Villanueva et al., 2015).

|  | | | | | | | | | |
| --- | --- | --- | --- | --- | --- | --- | --- | --- | --- |
|  | Soluble fraction | Lignin content | (Hemi)  cellulose | Polyphenols | | Ash | | Total N | Total C |
|  |  (g C kg^-1^ total C)  | | | | | | (g C kg^-1^ plant) | | |
|  | | | | | | | | | |
| Maize | 599 | 25 | 311 | 2 | 62 | | | 34 | 422 |
| NDF ^a^ | 0 | 63 | 776 | 6 | 155 | | | 19 | 405 |
|  | | | | | | | | | |

^a^ NDF: the neutral detergent fibre.
