# Supplementary material for: Young maize plants impact the bacterial community in Australian cotton‐sown vertisol more than agricultural practices
Source: Environ Microbiol Rep. 2025 Apr 30;17(3):e13322. doi: 10.1111/1758-2229.13322 (PMC12041893; doi:10.1111/1758-2229.13322)
Supplement: Supplementary file 13 — Table S3. Effect of agricultural practice (soil cultivated with cotton (Gossypium hirsutum L.) monoculture (summer cotton‐winter, fallow‐summer cotton) conventional tillage (CTCC), minimum tillage of continuous cotton (MITCC), and minimum tillage cotton‐wheat (Triticum aestivum L.) rotation (summer cotton‐winter wheat‐summer and winter fallow‐summer cotton) (MITCW)) in the unamended soil or soil amended with young maize plants, their neutral detergent fibre (NDF) fraction or urea, the effect of treatment (unamended soil or soil amended with young maize plants, their NDF fraction or urea) in the CTCC, MITCC, or MITCW soil, and the effect of time (day 0, 1, 3, 7, 14, and 28 days) on the Hill numbers at q = 0, 1 and 2. [file EMI4-17-e13322-s004.docx]

Table S3. Effect of agricultural practice (soil cultivated with cotton (*Gossypium hirsutum* L.) monoculture (summer cotton-winter, fallow-summer cotton) conventional tillage (CTCC), minimum tillage of continuous cotton (MITCC), and minimum tillage cotton-wheat (*Triticum aestivum* L.) rotation (summer cotton-winter wheat-summer and winter fallow-summer cotton) (MITCW)) in the unamended soil or soil amended with young maize plants, their neutral detergent fibre (NDF) fraction or urea, the effect of treatment (unamended soil or soil amended with young maize plants, their NDF fraction or urea) in the CTCC, MITCC or MITCW soil, and the effect of time (day 0, 1, 3, 7, 14 and 28 days) on the Hill numbers at *q* = 0, 1 and 2.

| ⎯⎯⎯⎯⎯⎯⎯⎯⎯⎯⎯⎯⎯⎯⎯⎯⎯⎯⎯⎯⎯⎯⎯⎯⎯⎯⎯⎯⎯⎯⎯⎯⎯⎯⎯⎯⎯⎯⎯⎯⎯⎯⎯⎯⎯⎯⎯⎯⎯⎯⎯ | | | | | | | | | | |
| --- | --- | --- | --- | --- | --- | --- | --- | --- | --- | --- |
|  | Hill numbers | | | | | | | | |  |
|  | ⎯⎯⎯⎯⎯⎯⎯⎯⎯⎯⎯⎯⎯⎯⎯⎯⎯⎯⎯⎯⎯⎯⎯⎯⎯⎯⎯⎯⎯⎯⎯⎯⎯⎯ | | | | | | | | |  |
|  | *q* = 0 | | *q* = 1 | | *q* = 2 | | | | |  |
|  | ⎯⎯⎯⎯⎯⎯⎯⎯⎯⎯ | | ⎯⎯⎯⎯⎯⎯⎯⎯⎯⎯ | | ⎯⎯⎯⎯⎯⎯⎯⎯⎯⎯⎯ | | | | |  |
| Effect ^a^ | F value | *p* value | F value | *p* value | F value | | *p* value | | |  |
| ⎯⎯⎯⎯⎯⎯⎯⎯⎯⎯⎯⎯⎯⎯⎯⎯⎯⎯⎯⎯⎯⎯⎯⎯⎯⎯⎯⎯⎯⎯⎯⎯⎯⎯⎯⎯⎯⎯⎯⎯⎯⎯⎯⎯⎯⎯⎯⎯⎯⎯⎯ | | | | | | | | | | |
| Agricultural practice |  |  |  |  |  |  | | |  |  |
| Unamended | 1.68 | 0.207 | 3.56 | **0.044** ^b^ | 3.86 | **0.035** | | |  |  |
| Young maize plants amended | 0.75 | 0.485 | 0.73 | 0.494 | 0.38 | 0.691 | | |  |  |
| Neutral detergent fraction amended | 0.18 | 0.826 | 0.08 | 0.925 | 0.05 | 0.948 | | |  |  |
| Urea amended | 0.41 | 0.670 | 1.18 | 0.333 | 0.97 | 0.399 | | |  |  |
|  |  |  |  |  |  |  | | |  |  |
| Treatment |  |  |  |  |  |  | | |  |  |
| CTCC | 0.18 | 0.912 | 0.69 | 0.571 | 0.71 | 0.559 | | |  |  |
| MITCC | 1.64 | 0.209 | 1.72 | 0.195 | 2.22 | 0.115 | | |  |  |
| MITCW | 0.52 | 0.674 | 0.65 | 0.592 | 0.42 | 0.742 | | |  |  |
|  |  |  |  |  |  |  | | |  |  |
| Time | 9.02 | **< 0.001** | 41.32 | **< 0.001** | 91.60 | **< 0.001** | | |  |  |
| ⎯⎯⎯⎯⎯⎯⎯⎯⎯⎯⎯⎯⎯⎯⎯⎯⎯⎯⎯⎯⎯⎯⎯⎯⎯⎯⎯⎯⎯⎯⎯⎯⎯⎯⎯⎯⎯⎯⎯⎯⎯⎯⎯⎯⎯⎯⎯⎯⎯ | | | | | | | |  |  |  |
| ^a^ The effect of agricultural practise, treatment, i.e. application of maize plants, NDF and urea, and time on the Hill numbers was determined with a t1way non-parametric analysis in the WRS2 package in R (Mair and Wilcox, 2017), ^b^ values in bold indicate a significant effect at *p* < 0.05. | | | | | | | |  |  |  |
| ⎯⎯⎯⎯⎯⎯⎯⎯⎯⎯⎯⎯⎯⎯⎯⎯⎯⎯⎯⎯⎯⎯⎯⎯⎯⎯⎯⎯⎯⎯⎯⎯⎯⎯⎯⎯⎯⎯⎯⎯⎯⎯⎯⎯⎯⎯⎯⎯⎯ | | | | | | | |  |  |  |
