# Supplementary material for: Young maize plants impact the bacterial community in Australian cotton‐sown vertisol more than agricultural practices
Source: Environ Microbiol Rep. 2025 Apr 30;17(3):e13322. doi: 10.1111/1758-2229.13322 (PMC12041893; doi:10.1111/1758-2229.13322)
Supplement: Supplementary file 14 — Table S4. Effect of agricultural practices on the relative abundance of bacterial groups assigned up to the taxonomic level of genus. Only bacterial groups with a large effect size ≤ −0.8 and ≥0.8 are given (Kim, 2015). The effect size, which is defined as the difference between groups divided by the maximum dispersion within group X or Y, was calculated with the aldex.ttest argument (ALDEx2 (version, 1.18), Gloor et al. (2020)). [file EMI4-17-e13322-s011.docx]

**Table S4.** Effect of agricultural practices on the relative abundance of bacterial groups assigned up to the taxonomic level of genus. Only bacterial groups with a large effect size ≤ -0.8 and ≥ 0.8 are given (Kim, 2015). The effect size, which is defined as the difference between groups divided by the maximum dispersion within group X or Y, was calculated with the aldex.ttest argument (ALDEx2 (version, 1.18), Gloor et al. (2020)).

| ⎯⎯⎯⎯⎯⎯⎯⎯⎯⎯⎯⎯⎯⎯⎯⎯⎯⎯⎯⎯⎯⎯⎯⎯⎯⎯⎯⎯⎯⎯⎯⎯⎯⎯⎯⎯⎯⎯⎯⎯⎯⎯⎯ | | |
| --- | --- | --- |
|  | Time ^a^ | Effect |
| **Tillage effect (CTCC vs MITCC)** | (days) | size ^b^ |
| ⎯⎯⎯⎯⎯⎯⎯⎯⎯⎯⎯⎯⎯⎯⎯⎯⎯⎯⎯⎯⎯⎯⎯⎯⎯⎯⎯⎯⎯⎯⎯⎯⎯⎯⎯⎯⎯⎯⎯⎯⎯⎯⎯ | | |
| **Acidobacteriota** |  |  |
| Acidobacteriota | 14 | 0.9 |
| Acidobacteriota | 28 | -1.0 |
| Acidobacteriae, Bryobacterales, Bryobacteraceae, *Bryobacter* | 0 | 1.3 |
| Acidobacteriae, Bryobacterales, Bryobacteraceae, *Bryobacter* | 3 | 0.8 |
| Blastocatellia, 11-24, 11-24, 11-24 | 0 | 1.0 |
| Blastocatellia, 11-24, 11-24, 11-24 | 1 | 1.0 |
| Blastocatellia, 11-24, 11-24, 11-24 | 28 | 0.8 |
| Blastocatellia, Blastocatellales, Blastocatellaceae | 1 | 1.0 |
| Blastocatellia, Blastocatellales, Blastocatellaceae | 28 | 1.0 |
| Blastocatellia, Blastocatellales, Blastocatellaceae, *Aridibacter* | 3 | 0.8 |
| Blastocatellia, Blastocatellales, Blastocatellaceae, *Blastocatella* | 7 | -0.9 |
| Blastocatellia, Blastocatellales, Blastocatellaceae, uncultured | 0 | 0.9 |
| Blastocatellia, Blastocatellales, Blastocatellaceae, uncultured | 14 | -2.0 |
| Blastocatellia, Blastocatellales, Blastocatellaceae, uncultured | 28 | 1.0 |
| Blastocatellia, Elev-16S-573, Elev-16S-573, *Elev-16S-573* | 1 | -1.0 |
| Blastocatellia, Pyrinomonadales, Pyrinomonadaceae, *RB41* | 0 | -1.4 |
| Blastocatellia, Pyrinomonadales, Pyrinomonadaceae, *RB41* | 1 | -1.4 |
| Holophagae, Subgroup_7, Subgroup_7, *Subgroup_7* | 28 | 0.8 |
| Subgroup_5, Subgroup_5, Subgroup_5, *Subgroup_5* | 3 | -0.8 |
| Vicinamibacteria, Vicinamibacterales | 0 | -1.0 |
| Vicinamibacteria, Vicinamibacterales | 3 | -0.8 |
| Vicinamibacteria, Vicinamibacterales | 7 | -1.8 |
| Vicinamibacteria, Vicinamibacterales, Vicinamibacteraceae, *Vicinamibacter* | 28 | 1.0 |
| Vicinamibacteria, Vicinamibacterales, Vicinamibacteraceae, Vicinamibacteraceae | 1 | -1.5 |
| Vicinamibacteria, Vicinamibacterales, Vicinamibacteraceae, Vicinamibacteraceae | 3 | -0.8 |
| Vicinamibacteria, Vicinamibacterales, Vicinamibacteraceae, Vicinamibacteraceae | 14 | -0.9 |
| **Actinomycetota** |  |  |
| Actinobacteria | 14 | -0.8 |
| Actinobacteria, Corynebacteriales, Nocardiaceae, *Rhodococcus* | 3 | 0.8 |
| Actinobacteria, Frankiales, Geodermatophilaceae, *Blastococcus* | 7 | 1.7 |
| Actinobacteria, Frankiales, Geodermatophilaceae, *Blastococcus* | 14 | -0.9 |
| Actinobacteria, Micrococcales, Intrasporangiaceae, *Tetrasphaera* | 28 | 0.9 |
| Actinobacteria, Micrococcales, Micrococcaceae, *Pseudarthrobacter* | 0 | 2.0 |
| Actinobacteria, Micrococcales, Promicromonosporaceae, *Promicromonospora* | 1 | -0.9 |
| Actinobacteria, Micromonosporales, Micromonosporaceae | 1 | 0.9 |
| Actinobacteria, Micromonosporales, Micromonosporaceae, *Dactylosporangium* | 3 | 0.8 |
| Actinobacteria, Micromonosporales, Micromonosporaceae, *Dactylosporangium* | 7 | -0.9 |
| Actinobacteria, Propionibacteriales, Nocardioidaceae | 14 | -1.1 |
| Actinobacteria, Propionibacteriales, Nocardioidaceae | 28 | 1.2 |
| Actinobacteria, Propionibacteriales, Nocardioidaceae, *Kribbella* | 7 | -0.8 |
| Actinobacteria, Propionibacteriales, Nocardioidaceae, *Nocardioides* | 14 | -1.7 |
| Actinobacteria, Propionibacteriales, Propionibacteriaceae, *Microlunatus* | 0 | 0.9 |
| Actinobacteria, Propionibacteriales, Propionibacteriaceae, *Microlunatus* | 28 | 0.9 |
| Actinobacteria, Streptomycetales, Streptomycetaceae | 28 | -0.9 |
| Actinobacteria, Streptomycetales, Streptomycetaceae, *Streptomyces* | 14 | -1.1 |
| MB-A2-108, MB-A2-108, MB-A2-108, MB-A2-108 | 3 | -1.2 |
| Rubrobacteria, Rubrobacterales, Rubrobacteriaceae, *Rubrobacter* | 0 | 0.9 |
| Rubrobacteria, Rubrobacterales, Rubrobacteriaceae, *Rubrobacter* | 1 | -1.1 |
| Rubrobacteria, Rubrobacterales, Rubrobacteriaceae, *Rubrobacter* | 7 | 2.1 |
| Rubrobacteria, Rubrobacterales, Rubrobacteriaceae, *Rubrobacter* | 14 | 2.0 |
| ⎯⎯⎯⎯⎯⎯⎯⎯⎯⎯⎯⎯⎯⎯⎯⎯⎯⎯⎯⎯⎯⎯⎯⎯⎯⎯⎯⎯⎯⎯⎯⎯⎯⎯⎯⎯⎯⎯⎯⎯⎯⎯⎯ | | |

Table S4. Continued.

| ⎯⎯⎯⎯⎯⎯⎯⎯⎯⎯⎯⎯⎯⎯⎯⎯⎯⎯⎯⎯⎯⎯⎯⎯⎯⎯⎯⎯⎯⎯⎯⎯⎯⎯⎯⎯⎯⎯⎯⎯⎯⎯⎯ | | |
| --- | --- | --- |
| Thermoleophilia, Gaiellales | 1 | -0.8 |
| Thermoleophilia, Gaiellales, Gaiellaceae, *Gaiella* | 7 | -1.0 |
| Thermoleophilia, Gaiellales, Gaiellaceae, *Gaiella* | 28 | -0.9 |
| Thermoleophilia, Gaiellales, uncultured, uncultured | 3 | -1.5 |
| Thermoleophilia, Gaiellales, uncultured, uncultured | 14 | -1.2 |
| **Armatimonadota** |  |  |
| Chthonomonadetes, Chthonomonadales, Chthonomonadales, Chthonomonadales | 3 | -0.8 |
| Chthonomonadetes, Chthonomonadales, Chthonomonadales, Chthonomonadales | 28 | -1.4 |
| Fimbriimonadia, Fimbriimonadales, Fimbriimonadaceae, Fimbriimonadaceae | 7 | -0.8 |
| uncultured, uncultured, uncultured, uncultured | 0 | -0.9 |
| **Bacteroidota** |  |  |
| Bacteroidia, Chitinophagales, Chitinophagaceae, *Flavisolibacter* | 7 | 0.8 |
| Bacteroidia, Chitinophagales, Chitinophagaceae, *Flavitalea* | 1 | 0.9 |
| Bacteroidia, Chitinophagales, Chitinophagaceae, uncultured | 14 | 0.8 |
| **Chloroflexota** |  |  |
| Anaerolineae, Anaerolineales, Anaerolineaceae | 14 | 0.9 |
| Anaerolineae, Anaerolineales, Anaerolineaceae, uncultured | 28 | -1.0 |
| Anaerolineae, Anaerolineales, Anaerolineaceae, *UTCFX1* | 7 | 2.0 |
| Anaerolineae, Anaerolineales, Anaerolineaceae, *UTCFX1* | 14 | 0.9 |
| Anaerolineae, Anaerolineales, Anaerolineaceae, *UTCFX1* | 28 | 0.9 |
| Anaerolineae, Caldilineales, Caldilineaceae, uncultured | 0 | -1.0 |
| Anaerolineae, SBR1031, A4b, *A4b* | 14 | 1.0 |
| Gitt-GS-136, Gitt-GS-136, Gitt-GS-136, *Gitt-GS-136* | 1 | -1.7 |
| Gitt-GS-136, Gitt-GS-136, Gitt-GS-136, *Gitt-GS-136* | 7 | 1.6 |
| Gitt-GS-136, Gitt-GS-136, Gitt-GS-136, *Gitt-GS-136* | 14 | -1.2 |
| Gitt-GS-136, Gitt-GS-136, Gitt-GS-136, *Gitt-GS-136* | 28 | -2.0 |
| KD4-96, KD4-96, KD4-96, KD4-96 | 14 | -0.8 |
| KD4-96, KD4-96, KD4-96, KD4-96 | 28 | -0.8 |
| Ktedonobacteria, C0119, C0119, *C0119* | 0 | 1.1 |
| Ktedonobacteria, C0119, C0119, *C0119* | 14 | -1.7 |
| OLB14, OLB14, OLB14, *OLB14* | 1 | -0.8 |
| P2-11E, P2-11E, P2-11E, *P2-11E* | 7 | -0.8 |
| **Bacillota** |  |  |
| Bacilli, Bacillales, Bacillaceae, Bacillus | 3 | -1.5 |
| Bacilli, Paenibacillales, Paenibacillaceae, Paenibacillus | 1 | 0.8 |
| **Gemmatimonadota** |  |  |
| BD2-11_terrestrial_group, BD2-11_terrestrial_group, BD2-11_terrestrial_group, BD2-11_terrestrial_group | 7 | -0.9 |
| Gemmatimonadetes, Gemmatimonadales, Gemmatimonadaceae | 7 | 1.4 |
| Gemmatimonadetes, Gemmatimonadales, Gemmatimonadaceae | 14 | 1.3 |
| Gemmatimonadetes, Gemmatimonadales, Gemmatimonadaceae | 28 | 0.9 |
| Gemmatimonadetes, Gemmatimonadales, Gemmatimonadaceae, *Roseisolibacter* | 1 | 1.5 |
| Gemmatimonadetes, Gemmatimonadales, Gemmatimonadaceae, *Roseisolibacter* | 14 | 1.1 |
| Gemmatimonadetes, Gemmatimonadales, Gemmatimonadaceae, uncultured | 14 | -0.8 |
| Longimicrobia, Longimicrobiales, Longimicrobiaceae, Longimicrobiaceae | 14 | 0.9 |
| Longimicrobia, Longimicrobiales, Longimicrobiaceae, Longimicrobiaceae | 28 | 0.8 |
| S0134_terrestrial_group, S0134_terrestrial_group, S0134_terrestrial_group, S0134_terrestrial_group | 0 | -0.9 |
| S0134_terrestrial_group, S0134_terrestrial_group, S0134_terrestrial_group, S0134_terrestrial_group | 14 | -1.1 |
| **Latescibacterota** |  |  |
| Latescibacterota, Latescibacterota, Latescibacterota, *Latescibacterota* | 1 | -0.9 |
| **Methylomirabilota** |  |  |
| Methylomirabilia, Rokubacteriales, Rokubacteriales, Rokubacteriales | 1 | -1.6 |
| Methylomirabilia, Rokubacteriales, Rokubacteriales, Rokubacteriales | 14 | -1.6 |
| Methylomirabilia, Rokubacteriales, Rokubacteriales, Rokubacteriales | 28 | -0.8 |
| ⎯⎯⎯⎯⎯⎯⎯⎯⎯⎯⎯⎯⎯⎯⎯⎯⎯⎯⎯⎯⎯⎯⎯⎯⎯⎯⎯⎯⎯⎯⎯⎯⎯⎯⎯⎯⎯⎯⎯⎯⎯⎯⎯ | | |

Table S4. Continued.

| ⎯⎯⎯⎯⎯⎯⎯⎯⎯⎯⎯⎯⎯⎯⎯⎯⎯⎯⎯⎯⎯⎯⎯⎯⎯⎯⎯⎯⎯⎯⎯⎯⎯⎯⎯⎯⎯⎯⎯⎯⎯⎯⎯ | | |
| --- | --- | --- |
| **Myxococcota** |  |  |
| bacteriap25, bacteriap25, bacteriap25, *bacteriap25* | 7 | -1.1 |
| Myxococcia, Myxococcales, Myxococcaceae, uncultured | 7 | -0.9 |
| Polyangia, Haliangiales, Haliangiaceae, *Haliangium* | 1 | 1.8 |
| Polyangia, Polyangiales, Polyangiaceae, *Pajaroellobacter* | 14 | -0.8 |
| Polyangia, Polyangiales, Polyangiaceae, *Sorangium* | 1 | -0.8 |
| **Nitrospirota** |  |  |
| Nitrospiria, Nitrospirales, Nitrospiraceae, *Nitrospira* | 0 | -1.1 |
| Nitrospiria, Nitrospirales, Nitrospiraceae, *Nitrospira* | 3 | -0.9 |
| Nitrospiria, Nitrospirales, Nitrospiraceae, *Nitrospira* | 14 | -0.9 |
| **Planctomycetota** |  |  |
| Phycisphaerae, Tepidisphaerales, WD2101_soil_group, *WD2101_soil_group* | 14 | 0.8 |
| **Pseudomonadata, Alphaproteobacteria** |  |  |
| Azospirillales, Azospirillaceae, *Skermanella* | 0 | 0.8 |
| Azospirillales, uncultured, uncultured | 0 | 1.2 |
| Azospirillales, uncultured, uncultured | 3 | -1.1 |
| Caulobacterales, Caulobacteraceae | 1 | 0.8 |
| Caulobacterales, Caulobacteraceae | 14 | 0.9 |
| Caulobacterales, Caulobacteraceae, *Brevundimonas* | 14 | 0.9 |
| Elsterales, uncultured, uncultured | 3 | 1.0 |
| Rhizobiales, Beijerinckiaceae, Neo-b11 | 28 | 1.0 |
| Rhizobiales, Devosiaceae, Devosia | 7 | 1.4 |
| Rhizobiales, Rhizobiaceae | 14 | -0.8 |
| Rhizobiales, Rhizobiales_Incertae_Sedis, *Bauldia* | 14 | 0.8 |
| Rhizobiales, Rhizobiales_Incertae_Sedis, *Nordella* | 14 | 1.5 |
| Rhizobiales, Xanthobacteraceae | 1 | -1.0 |
| Rhizobiales, Xanthobacteraceae, *Pseudolabrys* | 0 | -1.1 |
| Rhizobiales, Xanthobacteraceae, uncultured | 14 | -0.8 |
| Rhodobacterales, Rhodobacteraceae, *Rubellimicrobium* | 1 | 0.9 |
| Sphingomonadales, Sphingomonadaceae, *Sphingomonas* | 0 | 0.8 |
| Sphingomonadales, Sphingomonadaceae, *Sphingomonas* | 3 | 0.8 |
| uncultured, uncultured, uncultured | 3 | -0.8 |
| **Pseudomonadata, Gammaproteobacteria** |  |  |
| Burkholderiales, Comamonadaceae, *Ramlibacter* | 7 | 1.3 |
| Burkholderiales, Nitrosomonadaceae, *Ellin6067* | 28 | 0.9 |
| Burkholderiales, Nitrosomonadaceae, *MND1* | 3 | -1.0 |
| Burkholderiales, Oxalobacteraceae, *Massilia* | 7 | 1.5 |
| Burkholderiales, Oxalobacteraceae, *Noviherbaspirillum* | 14 | -0.8 |
| Burkholderiales, SC-I-84, *SC-I-84* | 7 | 0.8 |
| Burkholderiales, SC-I-84, *SC-I-84* | 28 | 1.2 |
| Burkholderiales, TRA3-20, *TRA3-20* | 0 | -0.8 |
| Burkholderiales, TRA3-20, *TRA3-20* | 7 | 1.0 |
| CCD24, CCD24, *CCD24* | 3 | -1.0 |
| CCD24, CCD24, *CCD24* | 14 | -0.9 |
| CCD24, CCD24, *CCD24* | 28 | 0.8 |
| Enterobacterales, Enterobacteriaceae | 0 | -0.8 |
| Oceanospirillales, Halomonadaceae, *Halomonas* | 3 | -1.7 |
| PLTA13, PLTA13, PLTA13 | 14 | -0.8 |
| Pseudomonadales, Pseudomonadaceae, *Pseudomonas* | 1 | 0.8 |
| Pseudomonadales, Pseudomonadaceae, *Pseudomonas* | 28 | -1.3 |
| Steroidobacterales, Steroidobacteraceae | 0 | -1.0 |
| Steroidobacterales, Steroidobacteraceae | 1 | 0.9 |
| Steroidobacterales, Steroidobacteraceae, *Steroidobacter* | 28 | 1.2 |
| ⎯⎯⎯⎯⎯⎯⎯⎯⎯⎯⎯⎯⎯⎯⎯⎯⎯⎯⎯⎯⎯⎯⎯⎯⎯⎯⎯⎯⎯⎯⎯⎯⎯⎯⎯⎯⎯⎯⎯⎯⎯⎯⎯ | | |

Table S4. Continued.

| ⎯⎯⎯⎯⎯⎯⎯⎯⎯⎯⎯⎯⎯⎯⎯⎯⎯⎯⎯⎯⎯⎯⎯⎯⎯⎯⎯⎯⎯⎯⎯⎯⎯⎯⎯⎯⎯⎯⎯⎯⎯⎯⎯ | | |
| --- | --- | --- |
| Xanthomonadales, Xanthomonadaceae, *Luteimonas* | 3 | -1.0 |
| Xanthomonadales, Xanthomonadaceae, *Lysobacter* | 3 | 1.8 |
| Xanthomonadales, Xanthomonadaceae, *Lysobacter* | 7 | 1.1 |
| Xanthomonadales, Xanthomonadaceae, *Lysobacter* | 28 | 2.7 |
| Xanthomonadales, Xanthomonadaceae, *Pseudoxanthomonas* | 1 | -0.9 |
| Xanthomonadales, Xanthomonadaceae, *Pseudoxanthomonas* | 3 | -0.8 |
| Xanthomonadales, Xanthomonadaceae, *Stenotrophomonas* | 28 | 0.9 |
| **Verrucomicrobiota** |  |  |
| Opitutales, Opitutaceae, *Opitutus* | 1 | -0.8 |
| Opitutales, Opitutaceae, *Opitutus* | 28 | 0.8 |
| ⎯⎯⎯⎯⎯⎯⎯⎯⎯⎯⎯⎯⎯⎯⎯⎯⎯⎯⎯⎯⎯⎯⎯⎯⎯⎯⎯⎯⎯⎯⎯⎯⎯⎯⎯⎯⎯⎯⎯⎯⎯⎯⎯ | | |
| **Crop rotation effect (MITCC vs MITCW)** |  |  |
| ⎯⎯⎯⎯⎯⎯⎯⎯⎯⎯⎯⎯⎯⎯⎯⎯⎯⎯⎯⎯⎯⎯⎯⎯⎯⎯⎯⎯⎯⎯⎯⎯⎯⎯⎯⎯⎯⎯⎯⎯⎯⎯⎯ | | |
| **Acidobacteriota** |  |  |
| Acidobacteriae, Bryobacterales, Bryobacteraceae, *Bryobacter* | d7 | 0.9 |
| Blastocatellia, 11-24, 11-24, 11-24 | d3 | 1 |
| Blastocatellia, Blastocatellales, Blastocatellaceae | d14 | 0.9 |
| Blastocatellia, Blastocatellales, Blastocatellaceae, *Aridibacter* | d0 | 0.9 |
| Blastocatellia, Blastocatellales, Blastocatellaceae, *Aridibacter* | d1 | -2.2 |
| Blastocatellia, Blastocatellales, Blastocatellaceae, *Aridibacter* | d3 | -0.8 |
| Blastocatellia, Blastocatellales, Blastocatellaceae, *Stenotrophobacter* | d7 | -0.9 |
| Blastocatellia, Blastocatellales, Blastocatellaceae, uncultured | d14 | 1 |
| Blastocatellia, Pyrinomonadales, Pyrinomonadaceae, *RB41* | d0 | 2 |
| Blastocatellia, Pyrinomonadales, Pyrinomonadaceae, *RB41* | d1 | 1.1 |
| Blastocatellia, Pyrinomonadales, Pyrinomonadaceae, *RB41* | d14 | 3 |
| Blastocatellia, Pyrinomonadales, Pyrinomonadaceae, *RB41* | d3 | 1.1 |
| Holophagae, Subgroup_7, Subgroup_7, *Subgroup_7* | d14 | 1 |
| Thermoanaerobaculia, Thermoanaerobaculales, Thermoanaerobaculaceae, *Subgroup_10* | d1 | 1 |
| Vicinamibacteria, Subgroup_17, Subgroup_17, *Subgroup_17* | d28 | 1.7 |
| Vicinamibacteria, Subgroup_17, Subgroup_17, *Subgroup_17* | d3 | 0.8 |
| Vicinamibacteria, Vicinamibacterales | d0 | 1.2 |
| Vicinamibacteria, Vicinamibacterales | d3 | 1.7 |
| Vicinamibacteria, Vicinamibacterales | d7 | 1.8 |
| Vicinamibacteria, Vicinamibacterales, Vicinamibacteraceae, uncultured | d1 | 1 |
| Vicinamibacteria, Vicinamibacterales, Vicinamibacteraceae, Vicinamibacter | d28 | -0.8 |
| Vicinamibacteria, Vicinamibacterales, Vicinamibacteraceae, Vicinamibacteraceae | d0 | 1.2 |
| Vicinamibacteria, Vicinamibacterales, Vicinamibacteraceae, Vicinamibacteraceae | d1 | 2 |
| Vicinamibacteria, Vicinamibacterales, Vicinamibacteraceae, Vicinamibacteraceae | d3 | 1.8 |
| Vicinamibacteria, Vicinamibacterales, Vicinamibacteraceae, Vicinamibacteraceae | d7 | 1 |
| **Actinomycetota** |  |  |
| Acidimicrobiia | d7 | 0.8 |
| Acidimicrobiia, IMCC26256, IMCC26256, *IMCC26256* | d28 | 0.8 |
| Actinobacteria, Frankiales, Geodermatophilaceae, *Blastococcus* | d7 | -0.9 |
| Actinobacteria, Micrococcales, Intrasporangiaceae, *Tetrasphaera* | d28 | -1 |
| Actinobacteria, Micrococcales, Micrococcaceae | d14 | 0.9 |
| Actinobacteria, Micrococcales, Micrococcaceae | d28 | -1 |
| Actinobacteria, Micrococcales, Micrococcaceae | d7 | 0.8 |
| Actinobacteria, Micrococcales, Micrococcaceae, *Pseudarthrobacter* | d28 | 0.9 |
| Actinobacteria, Propionibacteriales, Nocardioidaceae | d28 | -1.4 |
| Actinobacteria, Propionibacteriales, Nocardioidaceae, *Nocardioides* | d0 | -1.8 |
| Actinobacteria, Propionibacteriales, Propionibacteriaceae, *Microlunatus* | d0 | -2 |
| Actinobacteria, Propionibacteriales, Propionibacteriaceae, *Microlunatus* | d1 | 0.8 |
| Actinobacteria, Pseudonocardiales, Pseudonocardiaceae, *Pseudonocardia* | d7 | 0.8 |
| Actinobacteria, Streptomycetales, Streptomycetaceae, *Streptomyces* | d0 | -1.8 |
| MB-A2-108, MB-A2-108, MB-A2-108, MB-A2-108 | d0 | 1.2 |
| Rubrobacteria, Rubrobacterales, Rubrobacteriaceae, *Rubrobacter* | d0 | 1 |
| ⎯⎯⎯⎯⎯⎯⎯⎯⎯⎯⎯⎯⎯⎯⎯⎯⎯⎯⎯⎯⎯⎯⎯⎯⎯⎯⎯⎯⎯⎯⎯⎯⎯⎯⎯⎯⎯⎯⎯⎯⎯⎯⎯ | | |

Table S4. Continued.

| ⎯⎯⎯⎯⎯⎯⎯⎯⎯⎯⎯⎯⎯⎯⎯⎯⎯⎯⎯⎯⎯⎯⎯⎯⎯⎯⎯⎯⎯⎯⎯⎯⎯⎯⎯⎯⎯⎯⎯⎯⎯⎯⎯ | | |
| --- | --- | --- |
| Thermoleophilia, Gaiellales, Gaiellaceae, *Gaiella* | d3 | 1 |
| Thermoleophilia, Gaiellales, Gaiellaceae, *Gaiella* | d7 | 0.8 |
| Thermoleophilia, Gaiellales, uncultured, uncultured | d14 | 1.1 |
| Thermoleophilia, Gaiellales, uncultured, uncultured | d3 | 1.1 |
| Thermoleophilia, Solirubrobacterales, Solirubrobacteraceae, *Conexibacter* | d7 | 0.8 |
| **Armatimonadota** |  |  |
| uncultured, uncultured, uncultured, uncultured | d0 | 1.5 |
| **Bacillota** |  |  |
| Bacilli, Bacillales, Bacillaceae, *Bacillus* | d0 | 1.1 |
| Bacilli, Bacillales, Bacillaceae, *Bacillus* | d28 | 0.8 |
| Bacilli, Bacillales, Bacillaceae, *Bacillus* | d3 | 2 |
| Bacilli, Bacillales, Bacillaceae, *Bacillus* | d7 | -1.1 |
| **Bacteroidota** |  |  |
| Bacteroidia, Chitinophagales, Chitinophagaceae, *Flavisolibacter* | d0 | -0.8 |
| Bacteroidia, Chitinophagales, Chitinophagaceae, *Flavisolibacter* | d7 | -1.6 |
| Bacteroidia, Chitinophagales, Chitinophagaceae, uncultured | d0 | -1 |
| Bacteroidia, Cytophagales, Hymenobacteraceae, *Pontibacter* | d0 | 0.8 |
| Bacteroidia, Flavobacteriales, Flavobacteriaceae, *Flavobacterium* | d0 | -0.8 |
| **Chloroflexota** |  |  |
| Anaerolineae, Anaerolineales, Anaerolineaceae, uncultured | d7 | 1.1 |
| Anaerolineae, Anaerolineales, Anaerolineaceae, *UTCFX1* | d7 | -1.1 |
| Anaerolineae, Ardenticatenales, uncultured, uncultured | d14 | 0.9 |
| Anaerolineae, SBR1031, A4b, *A4b* | d0 | 1 |
| Anaerolineae, SBR1031, A4b, *OLB13* | d0 | -0.9 |
| Gitt-GS-136, Gitt-GS-136, Gitt-GS-136, *Gitt-GS-136* | d7 | -0.9 |
| Ktedonobacteria, C0119, C0119, *C0119* | d28 | -0.8 |
| Ktedonobacteria, C0119, C0119, *C0119* | d7 | -1 |
| TK10, TK10, TK10, *TK10* | d28 | 0.8 |
| **Desulfobacterota** |  |  |
| uncultured, uncultured, uncultured, uncultured | d1 | 0.9 |
| **Gemmatimonadota** |  |  |
| Gemmatimonadetes, Gemmatimonadales, Gemmatimonadaceae | d14 | -0.9 |
| Gemmatimonadetes, Gemmatimonadales, Gemmatimonadaceae | d3 | 1.1 |
| Gemmatimonadetes, Gemmatimonadales, Gemmatimonadaceae, *Gemmatimonas* | d14 | -0.8 |
| Gemmatimonadetes, Gemmatimonadales, Gemmatimonadaceae, uncultured | d14 | -0.8 |
| Gemmatimonadetes, Gemmatimonadales, Gemmatimonadaceae, uncultured | d28 | -0.8 |
| Gemmatimonadetes, Gemmatimonadales, Gemmatimonadaceae, uncultured | d3 | -1.5 |
| Longimicrobia, Longimicrobiales, Longimicrobiaceae, YC-ZSS-LKJ147 | d14 | 0.9 |
| S0134_terrestrial_group, S0134_terrestrial_group, S0134_terrestrial_group, *S0134_terrestrial_group* | d0 | 0.9 |
| S0134_terrestrial_group, S0134_terrestrial_group, S0134_terrestrial_group, *S0134_terrestrial_group* | d1 | 0.8 |
| S0134_terrestrial_group, S0134_terrestrial_group, S0134_terrestrial_group, *S0134_terrestrial_group* | d3 | 1.5 |
| **Methylomirabilota** |  |  |
| Methylomirabilia, Rokubacteriales, Rokubacteriales, Rokubacteriales | d1 | 1 |
| **Myxococcota** |  |  |
| bacteriap25, bacteriap25, bacteriap25, *bacteriap25* | d0 | 0.9 |
| bacteriap25, bacteriap25, bacteriap25, *bacteriap25* | d14 | 0.8 |
| Polyangia, Haliangiales, Haliangiaceae, *Haliangium* | d1 | -0.8 |
| **Nitrospirota** |  |  |
| Nitrospiria, Nitrospirales, Nitrospiraceae, *Nitrospira* | d0 | 0.8 |
| Nitrospiria, Nitrospirales, Nitrospiraceae, *Nitrospir*a | d3 | 0.9 |
| **Planctomycetota** |  |  |
| Phycisphaerae, Phycisphaerales, Phycisphaeraceae, *SM1A02* | d14 | 0.9 |
| Phycisphaerae, Tepidisphaerales, WD2101_soil_group, *WD2101_soil_group* | d0 | 1.6 |
| ⎯⎯⎯⎯⎯⎯⎯⎯⎯⎯⎯⎯⎯⎯⎯⎯⎯⎯⎯⎯⎯⎯⎯⎯⎯⎯⎯⎯⎯⎯⎯⎯⎯⎯⎯⎯⎯⎯⎯⎯⎯⎯⎯ | | |

Table S4. Continued.

| ⎯⎯⎯⎯⎯⎯⎯⎯⎯⎯⎯⎯⎯⎯⎯⎯⎯⎯⎯⎯⎯⎯⎯⎯⎯⎯⎯⎯⎯⎯⎯⎯⎯⎯⎯⎯⎯⎯⎯⎯⎯⎯⎯ | | |
| --- | --- | --- |
| **Pseudomonadata, Alphaproteobacteria** |  |  |
| Acetobacterales, Acetobacteraceae, *Craurococcus-Caldovatus* | d0 | -0.9 |
| Azospirillales, Azospirillaceae, *Skermanella* | d1 | 1 |
| Azospirillales, uncultured, uncultured | d0 | -1.9 |
| Caulobacterales, Caulobacteraceae, *Brevundimonas* | d14 | -0.8 |
| Reyranellales, Reyranellaceae, *Reyranella* | d3 | 0.8 |
| Rhizobiales | d0 | -0.9 |
| Rhizobiales, Beijerinckiaceae, *Microvirga* | d1 | 0.8 |
| Rhizobiales, Beijerinckiaceae, *Neo-b11* | d28 | -1.1 |
| Rhizobiales, Devosiaceae, *Devosia* | d7 | -0.9 |
| Rhizobiales, KF-JG30-B3, *KF-JG30-B3* | d0 | 0.8 |
| Rhizobiales, KF-JG30-B3, *KF-JG30-B3* | d28 | 0.8 |
| Rhizobiales, Rhizobiaceae, *Ochrobactrum* | d0 | -0.9 |
| Rhizobiales, Xanthobacteraceae | d0 | -0.8 |
| Rhizobiales, Xanthobacteraceae | d3 | -2.6 |
| Rhizobiales, Xanthobacteraceae, *Bradyrhizobium* | d7 | -0.8 |
| Sphingomonadales, Sphingomonadaceae | d28 | -1.6 |
| Sphingomonadales, Sphingomonadaceae, *Altererythrobacter* | d3 | -0.9 |
| **Pseudomonadata, Gammaproteobacteria** |  |  |
| Acidiferrobacterales, Acidiferrobacteraceae, *Sulfurifustis* | d7 | 0.9 |
| Burkholderiales, Comamonadaceae, *Methylibium* | d14 | -0.8 |
| Burkholderiales, Comamonadaceae, *Ramlibacter* | d0 | -0.9 |
| Burkholderiales, Comamonadaceae, *Ramlibacter* | d7 | -1.3 |
| Burkholderiales, Nitrosomonadaceae, *Ellin6067* | d14 | 1.7 |
| Burkholderiales, Nitrosomonadaceae, *Ellin6067* | d7 | -0.8 |
| Burkholderiales, Nitrosomonadaceae, *MND1* | d1 | 0.9 |
| Burkholderiales, Nitrosomonadaceae, *MND1* | d28 | 1.3 |
| Burkholderiales, Nitrosomonadaceae, *MND1* | d3 | 1 |
| Burkholderiales, Nitrosomonadaceae, *MND1* | d7 | 0.8 |
| Burkholderiales, Oxalobacteraceae | d3 | 0.9 |
| Burkholderiales, Oxalobacteraceae, *Massilia* | d7 | -1.7 |
| Burkholderiales, Oxalobacteraceae, *Noviherbaspirillum* | d3 | -0.8 |
| Burkholderiales, TRA3-20, *TRA3-20* | d0 | 0.8 |
| CCD24, CCD24, *CCD24* | d0 | 0.8 |
| CCD24, CCD24, *CCD24* | d28 | -0.8 |
| Oceanospirillales, Halomonadaceae, *Halomonas* | d28 | 1 |
| Oceanospirillales, Halomonadaceae, *Halomonas* | d7 | 0.9 |
| Pseudomonadales, Moraxellaceae, *Acinetobacter* | d7 | 1.7 |
| Pseudomonadales, Pseudomonadaceae, *Pseudomonas* | d28 | 1.1 |
| Pseudomonadales, Pseudomonadaceae, *Pseudomonas* | d3 | 1.6 |
| Steroidobacterales, Steroidobacteraceae | d0 | 1.9 |
| Steroidobacterales, Steroidobacteraceae | d1 | -1 |
| Steroidobacterales, Steroidobacteraceae | d28 | 1 |
| Steroidobacterales, Steroidobacteraceae, *Steroidobacter* | d28 | -2.4 |
| Xanthomonadales, Xanthomonadaceae, *Lysobacter* | d28 | -1.4 |
| ⎯⎯⎯⎯⎯⎯⎯⎯⎯⎯⎯⎯⎯⎯⎯⎯⎯⎯⎯⎯⎯⎯⎯⎯⎯⎯⎯⎯⎯⎯⎯⎯⎯⎯⎯⎯⎯⎯⎯⎯⎯⎯⎯ | | |
| **Tillage and crop rotation effect (CTCC versus MITCW)** |  |  |
| ⎯⎯⎯⎯⎯⎯⎯⎯⎯⎯⎯⎯⎯⎯⎯⎯⎯⎯⎯⎯⎯⎯⎯⎯⎯⎯⎯⎯⎯⎯⎯⎯⎯⎯⎯⎯⎯⎯⎯⎯⎯⎯⎯ | | |
| **Acidobacteriota** |  |  |
| other | 28 | -0.9 |
| Acidobacteriae, Bryobacterales, Bryobacteraceae, *Bryobacter* | 1 | 0.9 |
| Blastocatellia, 11-24, 11-24, *11-24* | 0 | 1.5 |
| Blastocatellia, 11-24, 11-24, *11-24* | 1 | 1.2 |
| Blastocatellia, 11-24, 11-24, *11-24* | 14 | 1.4 |
| Blastocatellia, 11-24, 11-24, *11-24* | 28 | 0.9 |
| ⎯⎯⎯⎯⎯⎯⎯⎯⎯⎯⎯⎯⎯⎯⎯⎯⎯⎯⎯⎯⎯⎯⎯⎯⎯⎯⎯⎯⎯⎯⎯⎯⎯⎯⎯⎯⎯⎯⎯⎯⎯⎯⎯ | | |

Table S4. Continued.

| ⎯⎯⎯⎯⎯⎯⎯⎯⎯⎯⎯⎯⎯⎯⎯⎯⎯⎯⎯⎯⎯⎯⎯⎯⎯⎯⎯⎯⎯⎯⎯⎯⎯⎯⎯⎯⎯⎯⎯⎯⎯⎯⎯ | | |
| --- | --- | --- |
| Blastocatellia, Blastocatellales, Blastocatellaceae | 14 | 0.9 |
| Blastocatellia, Blastocatellales, Blastocatellaceae | 28 | 2.3 |
| Blastocatellia, Blastocatellales, Blastocatellaceae, *Aridibacter* | 1 | -1.7 |
| Blastocatellia, Blastocatellales, Blastocatellaceae, *Blastocatella* | 7 | -1 |
| Blastocatellia, Blastocatellales, Blastocatellaceae, uncultured | 0 | 1.1 |
| Blastocatellia, Elev-16S-573, Elev-16S-573, *Elev-16S-573* | 1 | -1 |
| Blastocatellia, Pyrinomonadales, Pyrinomonadaceae, *RB41* | 3 | 0.9 |
| Blastocatellia, Pyrinomonadales, Pyrinomonadaceae, *RB41* | 14 | 2.5 |
| Blastocatellia, Pyrinomonadales, Pyrinomonadaceae, *RB41* | 28 | 1.4 |
| Holophagae, Subgroup_7, Subgroup_7, *Subgroup_7* | 7 | -0.8 |
| Holophagae, Subgroup_7, Subgroup_7, *Subgroup_7* | 14 | 1.3 |
| Holophagae, Subgroup_7, Subgroup_7, *Subgroup_7* | 28 | 1.6 |
| Thermoanaerobaculia, Thermoanaerobaculales, Thermoanaerobaculaceae, *Subgroup_10* | 14 | 0.8 |
| Vicinamibacteria, Subgroup_17, Subgroup_17, *Subgroup_17* | 7 | 0.8 |
| Vicinamibacteria, Vicinamibacterales | 14 | 1.2 |
| Vicinamibacteria, Vicinamibacterales | 28 | -1.1 |
| Vicinamibacteria, Vicinamibacterales, uncultured, uncultured | 28 | 1 |
| Vicinamibacteria, Vicinamibacterales, Vicinamibacteraceae | 1 | -0.8 |
| Vicinamibacteria, Vicinamibacterales, Vicinamibacteraceae | 3 | 1.1 |
| Vicinamibacteria, Vicinamibacterales, Vicinamibacteraceae, uncultured | 1 | 0.8 |
| Vicinamibacteria, Vicinamibacterales, Vicinamibacteraceae, Vicinamibacteraceae | 0 | 1 |
| Vicinamibacteria, Vicinamibacterales, Vicinamibacteraceae, Vicinamibacteraceae | 1 | 0.8 |
| Vicinamibacteria, Vicinamibacterales, Vicinamibacteraceae, Vicinamibacteraceae | 3 | 1 |
| **Actinomycetota** |  |  |
| Acidimicrobiia, uncultured, uncultured, uncultured | 14 | -0.8 |
| Actinobacteria, Micrococcales, Micrococcaceae | 7 | 0.8 |
| Actinobacteria, Micrococcales, Micrococcaceae | 28 | -0.8 |
| Actinobacteria, Micrococcales, Micrococcaceae, *Pseudarthrobacter* | 28 | 0.9 |
| Actinobacteria, Micrococcales, Promicromonosporaceae, *Promicromonospora* | 1 | -0.8 |
| Actinobacteria, Micromonosporales, Micromonosporaceae | 1 | 1 |
| Actinobacteria, Micromonosporales, Micromonosporaceae | 7 | 0.8 |
| Actinobacteria, Micromonosporales, Micromonosporaceae, *Actinoplanes* | 1 | -0.8 |
| Actinobacteria, Micromonosporales, Micromonosporaceae, *Dactylosporangium* | 7 | -0.8 |
| Actinobacteria, Propionibacteriales, Nocardioidaceae | 14 | -1.5 |
| Actinobacteria, Propionibacteriales, Nocardioidaceae, *Kribbella* | 7 | -0.9 |
| Actinobacteria, Propionibacteriales, Nocardioidaceae, *Nocardioides* | 0 | -0.9 |
| Actinobacteria, Propionibacteriales, Nocardioidaceae, *Nocardioides* | 14 | -1.4 |
| Actinobacteria, Propionibacteriales, Propionibacteriaceae, *Microlunatus* | 28 | 0.9 |
| Actinobacteria, Streptomycetales, Streptomycetaceae | 28 | -0.9 |
| Actinobacteria, Streptomycetales, Streptomycetaceae, *Streptomyces* | 1 | -0.9 |
| Actinobacteria, Streptomycetales, Streptomycetaceae, *Streptomyces* | 14 | -1 |
| Rubrobacteria, Rubrobacterales, Rubrobacteriaceae, *Rubrobacter* | 0 | 1.3 |
| Rubrobacteria, Rubrobacterales, Rubrobacteriaceae, *Rubrobacter* | 7 | 2.1 |
| Rubrobacteria, Rubrobacterales, Rubrobacteriaceae, *Rubrobacter* | 14 | 1 |
| Rubrobacteria, Rubrobacterales, Rubrobacteriaceae, *Rubrobacter* | 28 | 0.8 |
| Thermoleophilia, Gaiellales, Gaiellaceae, *Gaiella* | 1 | -1.3 |
| Thermoleophilia, Gaiellales, uncultured, uncultured | 1 | -0.8 |
| Thermoleophilia, Gaiellales, uncultured, uncultured | 7 | -1.3 |
| **Armatimonadota** |  |  |
| Chthonomonadetes, Chthonomonadales, Chthonomonadales, Chthonomonadales | 28 | -1.4 |
| Fimbriimonadia, Fimbriimonadales, Fimbriimonadaceae, Fimbriimonadaceae | 7 | -0.8 |
| **Bacillota** |  |  |
| Bacilli, Bacillales, Bacillaceae, Bacillus | 0 | 1.1 |
| **Bacteroidota** |  |  |
| Bacteroidia, Chitinophagales, Chitinophagaceae, *Flavisolibacter* | 28 | 0.9 |
| ⎯⎯⎯⎯⎯⎯⎯⎯⎯⎯⎯⎯⎯⎯⎯⎯⎯⎯⎯⎯⎯⎯⎯⎯⎯⎯⎯⎯⎯⎯⎯⎯⎯⎯⎯⎯⎯⎯⎯⎯⎯⎯⎯ | | |

Table S4. Continued.

| ⎯⎯⎯⎯⎯⎯⎯⎯⎯⎯⎯⎯⎯⎯⎯⎯⎯⎯⎯⎯⎯⎯⎯⎯⎯⎯⎯⎯⎯⎯⎯⎯⎯⎯⎯⎯⎯⎯⎯⎯⎯⎯⎯ | | |
| --- | --- | --- |
| **Chloroflexota** |  |  |
| Anaerolineae, Anaerolineales, Anaerolineaceae | 3 | -0.9 |
| Anaerolineae, Anaerolineales, Anaerolineaceae | 14 | 1 |
| Anaerolineae, Anaerolineales, Anaerolineaceae, uncultured | 0 | 1.6 |
| Anaerolineae, Anaerolineales, Anaerolineaceae, *UTCFX1* | 0 | 1 |
| Anaerolineae, Anaerolineales, Anaerolineaceae, *UTCFX1* | 7 | 0.8 |
| Anaerolineae, Anaerolineales, Anaerolineaceae, *UTCFX1* | 28 | 1 |
| Anaerolineae, SBR1031, A4b, *A4b* | 3 | 1.7 |
| Anaerolineae, SBR1031, A4b, *A4b* | 14 | 1.4 |
| Chloroflexia, Thermomicrobiales, JG30-KF-CM45, *JG30-KF-CM45* | 3 | 0.8 |
| Gitt-GS-136, Gitt-GS-136, Gitt-GS-136, *Gitt-GS-136* | 1 | -1.6 |
| Gitt-GS-136, Gitt-GS-136, Gitt-GS-136, *Gitt-GS-136* | 7 | 1.1 |
| Gitt-GS-136, Gitt-GS-136, Gitt-GS-136, *Gitt-GS-136* | 28 | -0.9 |
| Ktedonobacteria, C0119, C0119, *C0119* | 0 | 1.1 |
| Ktedonobacteria, C0119, C0119, *C0119* | 14 | -0.8 |
| **Desulfobacterota** |  |  |
| uncultured, uncultured, uncultured, uncultured | 1 | 0.9 |
| **Entotheonellaeota** |  |  |
| Entotheonellia, Entotheonellales, Entotheonellaceae, Entotheonellaceae | 14 | 1 |
| **Gemmatimonadota** |  |  |
| Gemmatimonadetes, Gemmatimonadales, Gemmatimonadaceae | 0 | 0.8 |
| Gemmatimonadetes, Gemmatimonadales, Gemmatimonadaceae | 3 | 1.3 |
| Gemmatimonadetes, Gemmatimonadales, Gemmatimonadaceae | 7 | 1.9 |
| Gemmatimonadetes, Gemmatimonadales, Gemmatimonadaceae | 28 | 1.1 |
| Gemmatimonadetes, Gemmatimonadales, Gemmatimonadaceae, *Roseisolibacter* | 14 | 1.3 |
| Gemmatimonadetes, Gemmatimonadales, Gemmatimonadaceae, uncultured | 3 | -1.4 |
| Gemmatimonadetes, Gemmatimonadales, Gemmatimonadaceae, uncultured | 14 | -1.1 |
| Longimicrobia, Longimicrobiales, Longimicrobiaceae, YC-ZSS-LKJ147 | 14 | 0.9 |
| S0134_terrestrial_group, S0134_terrestrial_group, S0134_terrestrial_group, *S0134_terrestrial_group* | 3 | 1.3 |
| S0134_terrestrial_group, S0134_terrestrial_group, S0134_terrestrial_group, *S0134_terrestrial_group* | 14 | -1.2 |
| S0134_terrestrial_group, S0134_terrestrial_group, S0134_terrestrial_group, *S0134_terrestrial_group* | 28 | 1.7 |
| **Methylomirabilota** |  |  |
| Methylomirabilia, Rokubacteriales, Rokubacteriales, Rokubacteriales | 14 | -1.3 |
| **Myxococcota** |  |  |
| bacteriap25, bacteriap25, bacteriap25, *bacteriap25* | 1 | -0.8 |
| bacteriap25, bacteriap25, bacteriap25, *bacteriap25* | 7 | -1.2 |
| Polyangia, Haliangiales, Haliangiaceae, *Haliangium* | 1 | 1.5 |
| Polyangia, Polyangiales, Polyangiaceae, *Pajaroellobacter* | 14 | -0.8 |
| **Nitrospirota** |  |  |
| Nitrospiria, Nitrospirales, Nitrospiraceae, *Nitrospira* | 14 | -0.9 |
| **Planctomycetota** |  |  |
| Phycisphaerae, Phycisphaerales, Phycisphaeraceae, *SM1A02* | 14 | 0.9 |
| Phycisphaerae, Tepidisphaerales, WD2101_soil_group, *WD2101_soil_group* | 0 | 0.8 |
| **Pseudomonadata, Alphaproteobacteria** |  |  |
| Dongiales, Dongiaceae, *Dongia* | 0 | -0.8 |
| Dongiales, Dongiaceae, *Dongia* | 7 | 0.9 |
| Elsterales, uncultured, uncultured | 3 | 0.9 |
| Elsterales, uncultured, uncultured | 14 | -1.1 |
| Reyranellales, Reyranellaceae, *Reyranella* | 28 | -0.9 |
| Rhizobiales, Beijerinckiaceae | 7 | 1.5 |
| Rhizobiales, KF-JG30-B3, *KF-JG30-B3* | 28 | 0.8 |
| Rhizobiales, Rhizobiaceae | 14 | -0.8 |
| Rhizobiales, Rhizobiaceae, *Ochrobactrum* | 0 | -1.1 |
| Rhizobiales, Xanthobacteraceae | 3 | -1.1 |
| ⎯⎯⎯⎯⎯⎯⎯⎯⎯⎯⎯⎯⎯⎯⎯⎯⎯⎯⎯⎯⎯⎯⎯⎯⎯⎯⎯⎯⎯⎯⎯⎯⎯⎯⎯⎯⎯⎯⎯⎯⎯⎯⎯ | | |

Table S4. Continued.

| ⎯⎯⎯⎯⎯⎯⎯⎯⎯⎯⎯⎯⎯⎯⎯⎯⎯⎯⎯⎯⎯⎯⎯⎯⎯⎯⎯⎯⎯⎯⎯⎯⎯⎯⎯⎯⎯⎯⎯⎯⎯⎯⎯ | | |
| --- | --- | --- |
| Sphingomonadales, Sphingomonadaceae | 28 | -0.8 |
| uncultured, uncultured, uncultured | 3 | -0.8 |
| **Pseudomonadata, Gammaproteobacteria** |  |  |
| Acidiferrobacterales, Acidiferrobacteraceae, *Sulfurifustis* | 7 | 0.8 |
| Burkholderiales | 3 | -0.8 |
| Burkholderiales, Comamonadaceae | 0 | 0.9 |
| Burkholderiales, Comamonadaceae | 28 | 1.1 |
| Burkholderiales, Comamonadaceae, *Rhizobacter* | 14 | -0.8 |
| Burkholderiales, Nitrosomonadaceae, *Ellin6067* | 14 | 1.6 |
| Burkholderiales, Nitrosomonadaceae, *Ellin6067* | 28 | 2.5 |
| Burkholderiales, Nitrosomonadaceae, *MND1* | 7 | 0.8 |
| Burkholderiales, Oxalobacteraceae | 1 | 0.8 |
| Burkholderiales, Oxalobacteraceae, *Noviherbaspirillum* | 1 | -0.8 |
| Burkholderiales, Oxalobacteraceae, *Noviherbaspirillum* | 28 | -0.9 |
| Burkholderiales, SC-I-84, *SC-I-84* | 28 | 0.9 |
| CCD24, CCD24, CCD24 | 0 | 1.0 |
| Oceanospirillales, Halomonadaceae, *Halomonas* | 3 | -0.9 |
| Pseudomonadales, Pseudomonadaceae, *Pseudomonas* | 3 | 1.7 |
| Pseudomonadales, Pseudomonadaceae, *Pseudomonas* | 14 | -1.0 |
| Steroidobacterales, Steroidobacteraceae, *Steroidobacter* | 7 | -1.8 |
| Xanthomonadales, Xanthomonadaceae, *Luteimonas* | 3 | -1.8 |
| Xanthomonadales, Xanthomonadaceae, *Lysobacter* | 3 | 1 |
| Xanthomonadales, Xanthomonadaceae, *Lysobacter* | 7 | 0.9 |
| Xanthomonadales, Xanthomonadaceae, *Lysobacter* | 28 | 2.0 |
| Xanthomonadales, Xanthomonadaceae, *Pseudoxanthomonas* | 1 | -0.8 |
| Xanthomonadales, Xanthomonadaceae, *Pseudoxanthomonas* | 3 | -0.9 |
| **Verrucomicrobiota** |  |  |
| Verrucomicrobiae, Opitutales, Opitutaceae, *Opitutus* | 7 | -1.1 |
| ⎯⎯⎯⎯⎯⎯⎯⎯⎯⎯⎯⎯⎯⎯⎯⎯⎯⎯⎯⎯⎯⎯⎯⎯⎯⎯⎯⎯⎯⎯⎯⎯⎯⎯⎯⎯⎯⎯⎯⎯⎯⎯⎯ | | |

^a^ Time of aerobic incubation, ^b^ Effect size: a negative value indicates that the relative abundance of the bacterial group in soil of the first mentioned agricultural practice is larger than in the second and a positive value the opposite.

| ⎯⎯⎯⎯⎯⎯⎯⎯⎯⎯⎯⎯⎯⎯⎯⎯⎯⎯⎯⎯⎯⎯⎯⎯⎯⎯⎯⎯⎯⎯⎯⎯⎯⎯⎯⎯⎯⎯⎯⎯⎯⎯⎯ |
| --- |
