# Supplementary material for: Young maize plants impact the bacterial community in Australian cotton‐sown vertisol more than agricultural practices
Source: Environ Microbiol Rep. 2025 Apr 30;17(3):e13322. doi: 10.1111/1758-2229.13322 (PMC12041893; doi:10.1111/1758-2229.13322)
Supplement: Supplementary file 15 — Table S5. Putative metabolic functions with an effect size (≤ −1.4 and ≥1.4) when comparing the relative abundance of putative metabolic functions in soil cultivated with cotton (Gossypium hirsutum L.) monoculture (summer cotton‐winter, fallow‐summer cotton) conventional tillage (CTCC), minimum tillage of continuous cotton (MITCC), and minimum tillage cotton‐wheat (Triticum aestivum L.) rotation (summer cotton‐winter wheat‐summer and winter fallow‐summer cotton) (MITCW) after 1, 3, 7, 14, or 28 days aerobic incubation. [file EMI4-17-e13322-s007.docx]

**Table S5** Putative metabolic functions with an effect size (≤ -1.4 and ≥ 1.4) when comparing the relative abundance of putative metabolic functions in soil cultivated with cotton (*Gossypium hirsutum* L.) monoculture (summer cotton-winter, fallow-summer cotton) conventional tillage (CTCC), minimum tillage of continuous cotton (MITCC), and minimum tillage cotton-wheat (*Triticum aestivum* L.) rotation (summer cotton-winter wheat-summer and winter fallow-summer cotton) (MITCW) after 1, 3, 7, 14 or 28 days aerobic incubation.

| ⎯⎯⎯⎯⎯⎯⎯⎯⎯⎯⎯⎯⎯⎯⎯⎯⎯⎯⎯⎯⎯⎯⎯⎯⎯⎯⎯⎯⎯⎯⎯⎯⎯⎯⎯⎯⎯⎯⎯⎯⎯⎯⎯⎯⎯⎯⎯⎯⎯⎯⎯ | | | | | | |
| --- | --- | --- | --- | --- | --- | --- |
| **CTCC vs MITCC** | | | | | | |
| ⎯⎯⎯⎯⎯⎯⎯⎯⎯⎯⎯⎯⎯⎯⎯⎯⎯⎯⎯⎯⎯⎯⎯⎯⎯⎯⎯⎯⎯⎯⎯⎯⎯⎯⎯⎯⎯⎯⎯⎯⎯⎯⎯⎯⎯⎯⎯⎯⎯⎯⎯ | | | | | | |
| Putative metabolic function | A ^a^ | | B ^b,c^ | Putative metabolic function | A | B |
| ⎯⎯⎯⎯⎯⎯⎯⎯⎯⎯⎯⎯⎯⎯⎯⎯⎯⎯⎯⎯⎯⎯⎯⎯⎯⎯⎯⎯⎯⎯⎯⎯⎯⎯⎯⎯⎯⎯⎯⎯⎯⎯⎯⎯⎯⎯⎯⎯⎯⎯⎯ | | | | | | |
| Betalain biosynthesis | | 7 | -1.7 | Flagellar assembly | 3 | -1.5 |
| D-Arginine and D-ornithine metabolism | | 0 | -1.5 | Lipopolysaccharide biosynthesis | 0 | -1.7 |
| D-Arginine and D-ornithine metabolism | | 1 | -1.6 | Lipopolysaccharide biosynthesis | 1 | -2.2 |
| Flagellar assembly | | 1 | -1.4 | Pentose phosphate pathway | 3 | -1.4 |
| ⎯⎯⎯⎯⎯⎯⎯⎯⎯⎯⎯⎯⎯⎯⎯⎯⎯⎯⎯⎯⎯⎯⎯⎯⎯⎯⎯⎯⎯⎯⎯⎯⎯⎯⎯⎯⎯⎯⎯⎯⎯⎯⎯⎯⎯⎯⎯⎯⎯⎯⎯ | | | | | | |
| **CTCC vs MITWC** | | | | | | |
| ⎯⎯⎯⎯⎯⎯⎯⎯⎯⎯⎯⎯⎯⎯⎯⎯⎯⎯⎯⎯⎯⎯⎯⎯⎯⎯⎯⎯⎯⎯⎯⎯⎯⎯⎯⎯⎯⎯⎯⎯⎯⎯⎯⎯⎯⎯⎯⎯⎯⎯⎯ | | | | | | |
| Alanine, aspartate and glutamate metabolism | | 14 | 2.2 | Lipoic acid metabolism | 14 | 2.5 |
| Amino sugar and nucleotide sugar metabolism | | 14 | 2.2 | Lipopolysaccharide biosynthesis | 14 | 1.8 |
| Aminoacyl-tRNA biosynthesis | | 14 | 1.9 | Lysine biosynthesis | 14 | 2.1 |
| Arginine and proline metabolism | | 14 | 2.1 | Lysine degradation | 14 | 1.4 |
| Atrazine degradation | | 1 | -1.6 | Methane metabolism | 14 | 1.5 |
| Bacterial secretion system | | 14 | 1.6 | Mismatch repair | 14 | 1.9 |
| Base excision repair | | 14 | 1.5 | N-Glycan biosynthesis | 14 | 1.4 |
| beta-Alanine metabolism | | 14 | 1.5 | Nicotinate and nicotinamide metabolism | 14 | 2.1 |
| Betalain biosynthesis | | 3 | 1.7 | Nitrogen metabolism | 14 | 1.9 |
| Betalain biosynthesis | | 14 | 1.5 | Nucleotide excision repair | 14 | 1.9 |
| Biosynthesis of ansamycins | | 14 | 1.7 | One carbon pool by folate | 14 | 1.8 |
| Biosynthesis of unsaturated fatty acids | | 14 | 1.7 | Other glycan degradation | 7 | -2.6 |
| Biosynthesis of vancomycin group antibiotics | | 14 | 2.3 | Oxidative phosphorylation | 14 | 1.8 |
| Butanoate metabolism | | 14 | 1.9 | Pantothenate and CoA biosynthesis | 14 | 2.0 |
| C5-Branched dibasic acid metabolism | | 14 | 2.0 | Penicillin and cephalosporin biosynthesis | 3 | 1.5 |
| Carbon fixation in photosynthetic organisms | | 14 | 2.0 | Penicillin and cephalosporin biosynthesis | 14 | 2.1 |
| Carbon fixation pathways in prokaryotes | | 14 | 2.3 | Pentose and glucuronate interconversions | 14 | 1.7 |
| Cell cycle - Caulobacter | | 14 | 2.2 | Pentose phosphate pathway | 14 | 2.1 |
| Citrate cycle (TCA cycle) | | 14 | 2.0 | Peptidoglycan biosynthesis | 14 | 2.1 |
| Cysteine and methionine metabolism | | 14 | 2.3 | Peroxisome | 14 | 1.6 |
| D-Alanine metabolism | | 14 | 2.2 | Phenylalanine, tyrosine and tryptophan biosynthesis | 14 | 2.0 |
| D-Glutamine and D-glutamate metabolism | | 14 | 2.0 | Phenylalanine metabolism | 14 | 1.7 |
| DNA replication | | 14 | 2.1 | Phosphotransferase system (PTS) | 14 | 1.8 |
| Drug metabolism - other enzymes | | 14 | 2.7 | Photosynthesis | 14 | 2.0 |
| Fatty acid biosynthesis | | 14 | 2.1 | Porphyrin and chlorophyll metabolism | 14 | 1.7 |
| Fatty acid metabolism | | 14 | 1.8 | Propanoate metabolism | 14 | 1.8 |
| Folate biosynthesis | | 14 | 1.8 | Protein export | 14 | 2.2 |
| Fructose and mannose metabolism | | 14 | 2.4 | Purine metabolism | 14 | 2.0 |
| Galactose metabolism | | 14 | 2.1 | Pyrimidine metabolism | 14 | 2.1 |
| Glutathione metabolism | | 14 | 1.7 | Pyruvate metabolism | 14 | 1.9 |
| Glycerolipid metabolism | | 14 | 1.8 | Riboflavin metabolism | 14 | 1.9 |
| Glycerophospholipid metabolism | | 14 | 1.8 | Ribosome | 14 | 2.1 |
| Glycine, serine and threonine metabolism | | 14 | 2.3 | RNA degradation | 14 | 1.8 |
| Glycolysis / Gluconeogenesis | | 14 | 1.8 | RNA polymerase | 14 | 2.2 |
| Glycosaminoglycan degradation | | 1 | -2.8 | Selenocompound metabolism | 14 | 2.5 |
| Glyoxylate and dicarboxylate metabolism | | 14 | 1.9 | Sphingolipid metabolism | 7 | -1.4 |
| Histidine metabolism | | 14 | 2.2 | Starch and sucrose metabolism | 14 | 1.7 |
| Homologous recombination | | 14 | 2.0 | Streptomycin biosynthesis | 14 | 2.2 |
| Inositol phosphate metabolism | | 14 | 1.5 | Styrene degradation | 14 | 1.8 |
| Linoleic acid metabolism | | 3 | -1.5 | Sulfur metabolism | 14 | 1.5 |
| ⎯⎯⎯⎯⎯⎯⎯⎯⎯⎯⎯⎯⎯⎯⎯⎯⎯⎯⎯⎯⎯⎯⎯⎯⎯⎯⎯⎯⎯⎯⎯⎯⎯⎯⎯⎯⎯⎯⎯⎯⎯⎯⎯⎯⎯⎯⎯⎯⎯⎯⎯ | | | | | | |

**Table S5** Continued.

| ⎯⎯⎯⎯⎯⎯⎯⎯⎯⎯⎯⎯⎯⎯⎯⎯⎯⎯⎯⎯⎯⎯⎯⎯⎯⎯⎯⎯⎯⎯⎯⎯⎯⎯⎯⎯⎯⎯⎯⎯⎯⎯⎯⎯⎯⎯⎯⎯⎯⎯⎯ | | | | | |
| --- | --- | --- | --- | --- | --- |
| Sulfur relay system | 14 | 2.2 | Tyrosine metabolism | 14 | 1.5 |
| Synthesis and degradation of ketone bodies | 14 | 1.4 | Ubiquinone and other terpenoid-quinone biosynthesis | 14 | 1.9 |
| Taurine and hypotaurine metabolism | 14 | 2.5 | Valine, leucine and isoleucine biosynthesis | 14 | 2.2 |
| Terpenoid backbone biosynthesis | 14 | 2.0 | Valine, leucine and isoleucine degradation | 14 | 1.9 |
| Thiamine metabolism | 14 | 2.5 | Vitamin B6 metabolism | 14 | 2.0 |
| Toluene degradation | 14 | 1.9 | Xylene degradation | 1 | -1.4 |
| Tropane, piperidine and pyridine alkaloid biosynthesis | 14 | 1.4 | Zeatin biosynthesis | 14 | 1.8 |
| Tryptophan metabolism | 14 | 1.7 |  |  |  |
| ⎯⎯⎯⎯⎯⎯⎯⎯⎯⎯⎯⎯⎯⎯⎯⎯⎯⎯⎯⎯⎯⎯⎯⎯⎯⎯⎯⎯⎯⎯⎯⎯⎯⎯⎯⎯⎯⎯⎯⎯⎯⎯⎯⎯⎯⎯⎯⎯⎯⎯⎯ | | | | | |
| **MITCC vs MITWC** | | | | | |
| ⎯⎯⎯⎯⎯⎯⎯⎯⎯⎯⎯⎯⎯⎯⎯⎯⎯⎯⎯⎯⎯⎯⎯⎯⎯⎯⎯⎯⎯⎯⎯⎯⎯⎯⎯⎯⎯⎯⎯⎯⎯⎯⎯⎯⎯⎯⎯⎯⎯⎯⎯ | | | | | |
| Alanine, aspartate and glutamate metabolism | 0 | 2.8 | D-Glutamine and D-glutamate metabolism | 1 | 1.4 |
| Alanine, aspartate and glutamate metabolism | 14 | 2.0 | D-Glutamine and D-glutamate metabolism | 14 | 2.5 |
| Amino sugar and nucleotide sugar metabolism | 0 | 2.2 | DNA replication | 0 | 2.4 |
| Amino sugar and nucleotide sugar metabolism | 1 | 1.9 | DNA replication | 14 | 1.9 |
| Amino sugar and nucleotide sugar metabolism | 14 | 2.3 | Drug metabolism - other enzymes | 0 | 3.1 |
| Aminoacyl-tRNA biosynthesis | 0 | 2.5 | Drug metabolism - other enzymes | 1 | 1.7 |
| Aminoacyl-tRNA biosynthesis | 1 | 1.6 | Drug metabolism - other enzymes | 14 | 2.3 |
| Aminoacyl-tRNA biosynthesis | 14 | 2.3 | Fatty acid biosynthesis | 0 | 2.8 |
| Arginine and proline metabolism | 0 | 2.2 | Fatty acid biosynthesis | 14 | 2.2 |
| Arginine and proline metabolism | 14 | 1.5 | Fatty acid metabolism | 0 | 1.6 |
| Bacterial secretion system | 0 | 2.1 | Fatty acid metabolism | 14 | 1.6 |
| Base excision repair | 0 | 1.8 | Flavonoid biosynthesis | 0 | 1.6 |
| Base excision repair | 14 | 1.6 | Flavonoid biosynthesis | 14 | 1.4 |
| beta-Alanine metabolism | 14 | 1.4 | Folate biosynthesis | 0 | 2.7 |
| Betalain biosynthesis | 0 | 3.6 | Folate biosynthesis | 14 | 1.7 |
| Betalain biosynthesis | 3 | 2.2 | Fructose and mannose metabolism | 0 | 1.7 |
| Betalain biosynthesis | 14 | 2.4 | Fructose and mannose metabolism | 14 | 2.3 |
| Biosynthesis of ansamycins | 0 | 2.8 | Galactose metabolism | 0 | 2.6 |
| Biosynthesis of ansamycins | 14 | 2.0 | Galactose metabolism | 1 | 1.8 |
| Biosynthesis of unsaturated fatty acids | 0 | 2.1 | Galactose metabolism | 14 | 2.0 |
| Biosynthesis of unsaturated fatty acids | 14 | 1.8 | Glycerolipid metabolism | 0 | 1.9 |
| Biosynthesis of vancomycin group antibiotics | 0 | 3.4 | Glycerolipid metabolism | 14 | 1.4 |
| Biosynthesis of vancomycin group antibiotics | 14 | 2.5 | Glycerophospholipid metabolism | 0 | 1.9 |
| Biotin metabolism | 0 | 1.5 | Glycerophospholipid metabolism | 14 | 1.7 |
| Butanoate metabolism | 0 | 2.0 | Glycine, serine and threonine metabolism | 0 | 2.7 |
| Butanoate metabolism | 14 | 1.4 | Glycine, serine and threonine metabolism | 14 | 1.8 |
| C5-Branched dibasic acid metabolism | 0 | 3.1 | Glycolysis / Gluconeogenesis | 0 | 1.8 |
| C5-Branched dibasic acid metabolism | 1 | 1.5 | Glycolysis / Gluconeogenesis | 14 | 1.6 |
| C5-Branched dibasic acid metabolism | 14 | 2.1 | Glyoxylate and dicarboxylate metabolism | 0 | 2.0 |
| Carbon fixation in photosynthetic organisms | 0 | 3.3 | Histidine metabolism | 0 | 2.9 |
| Carbon fixation in photosynthetic organisms | 14 | 2.0 | Histidine metabolism | 14 | 2.0 |
| Carbon fixation pathways in prokaryotes | 0 | 3.2 | Homologous recombination | 0 | 2.5 |
| Carbon fixation pathways in prokaryotes | 1 | 2.0 | Homologous recombination | 14 | 1.8 |
| Carbon fixation pathways in prokaryotes | 14 | 2.2 | Inositol phosphate metabolism | 0 | 1.5 |
| Cell cycle - Caulobacter | 0 | 3.0 | Lipoic acid metabolism | 0 | 3.2 |
| Cell cycle - Caulobacter | 1 | 1.6 | Lipoic acid metabolism | 14 | 2.5 |
| Cell cycle - Caulobacter | 14 | 2.1 | Lipopolysaccharide biosynthesis | 0 | 3.5 |
| Citrate cycle (TCA cycle) | 0 | 2.9 | Lipopolysaccharide biosynthesis | 14 | 2.0 |
| Citrate cycle (TCA cycle) | 14 | 2.1 | Lysine biosynthesis | 0 | 3.0 |
| Cysteine and methionine metabolism | 0 | 3.1 | Lysine biosynthesis | 14 | 2.1 |
| Cysteine and methionine metabolism | 14 | 2.1 | Lysine degradation | 0 | 1.5 |
| D-Alanine metabolism | 0 | 2.7 | Methane metabolism | 0 | 1.5 |
| D-Alanine metabolism | 14 | 2.0 | Mismatch repair | 0 | 2.3 |
| D-Glutamine and D-glutamate metabolism | 0 | 2.6 | Mismatch repair | 14 | 2.2 |
| ⎯⎯⎯⎯⎯⎯⎯⎯⎯⎯⎯⎯⎯⎯⎯⎯⎯⎯⎯⎯⎯⎯⎯⎯⎯⎯⎯⎯⎯⎯⎯⎯⎯⎯⎯⎯⎯⎯⎯⎯⎯⎯⎯⎯⎯⎯⎯⎯⎯⎯⎯ | | | | | |

**Table S5** Continued.

| ⎯⎯⎯⎯⎯⎯⎯⎯⎯⎯⎯⎯⎯⎯⎯⎯⎯⎯⎯⎯⎯⎯⎯⎯⎯⎯⎯⎯⎯⎯⎯⎯⎯⎯⎯⎯⎯⎯⎯⎯⎯⎯⎯⎯⎯⎯⎯⎯⎯⎯⎯ | | | | | |
| --- | --- | --- | --- | --- | --- |
| N-Glycan biosynthesis | 0 | 1.8 | Ribosome | 0 | 2.6 |
| Nicotinate and nicotinamide metabolism | 0 | 3.5 | Ribosome | 1 | 1.6 |
| Nicotinate and nicotinamide metabolism | 14 | 2.3 | Ribosome | 14 | 2.3 |
| Nitrogen metabolism | 0 | 1.6 | RNA degradation | 0 | 2.3 |
| Non-homologous end-joining | 7 | -1.4 | RNA degradation | 14 | 1.5 |
| Nucleotide excision repair | 0 | 2.3 | RNA polymerase | 0 | 3.2 |
| Nucleotide excision repair | 14 | 2.0 | RNA polymerase | 1 | 1.5 |
| One carbon pool by folate | 0 | 2.7 | RNA polymerase | 14 | 2.0 |
| One carbon pool by folate | 14 | 1.8 | Selenocompound metabolism | 0 | 3.4 |
| Other glycan degradation | 7 | -2.8 | Selenocompound metabolism | 1 | 1.5 |
| Oxidative phosphorylation | 0 | 2.9 | Sphingolipid metabolism | 7 | -1.5 |
| Oxidative phosphorylation | 14 | 1.8 | Starch and sucrose metabolism | 0 | 2.5 |
| Pantothenate and CoA biosynthesis | 0 | 3.0 | Starch and sucrose metabolism | 14 | 1.6 |
| Pantothenate and CoA biosynthesis | 14 | 2.2 | Streptomycin biosynthesis | 0 | 3.5 |
| Penicillin and cephalosporin biosynthesis | 14 | 1.4 | Streptomycin biosynthesis | 14 | 2.1 |
| Pentose and glucuronate interconversions | 0 | 2.0 | Sulfur metabolism | 0 | 2.0 |
| Pentose and glucuronate interconversions | 14 | 1.9 | Sulfur relay system | 0 | 3.8 |
| Pentose phosphate pathway | 0 | 2.7 | Sulfur relay system | 14 | 1.9 |
| Pentose phosphate pathway | 14 | 2.2 | Taurine and hypotaurine metabolism | 0 | 2.0 |
| Peptidoglycan biosynthesis | 0 | 3.0 | Taurine and hypotaurine metabolism | 14 | 2.1 |
| Peptidoglycan biosynthesis | 1 | 1.4 | Terpenoid backbone biosynthesis | 0 | 2.7 |
| Peptidoglycan biosynthesis | 14 | 2.2 | Terpenoid backbone biosynthesis | 14 | 2.2 |
| Peroxisome | 0 | 1.4 | Tetracycline biosynthesis | 7 | 4.5 |
| Phenylalanine, tyrosine and tryptophan biosynthesis | 0 | 3.2 | Tetracycline biosynthesis | 14 | -3.9 |
| Phenylalanine, tyrosine and tryptophan biosynthesis | 14 | 2.2 | Thiamine metabolism | 0 | 2.9 |
| Phenylalanine metabolism | 14 | 1.4 | Thiamine metabolism | 1 | 1.9 |
| Photosynthesis | 0 | 2.5 | Thiamine metabolism | 14 | 2.3 |
| Photosynthesis | 1 | 1.6 | Toluene degradation | 0 | 2.7 |
| Photosynthesis | 14 | 2.0 | Toluene degradation | 14 | 2.1 |
| Porphyrin and chlorophyll metabolism | 0 | 2.0 | Tropane, piperidine and pyridine alkaloid biosynthesis | 0 | 1.8 |
| Propanoate metabolism | 0 | 2.5 | Tropane, piperidine and pyridine alkaloid biosynthesis | 14 | 1.6 |
| Propanoate metabolism | 14 | 2.1 | Tyrosine metabolism | 14 | 1.4 |
| Protein digestion and absorption | 0 | 1.5 | Ubiquinone and other terpenoid-quinone biosynthesis | 0 | 2.4 |
| Protein export | 0 | 3.1 | Ubiquinone and other terpenoid-quinone biosynthesis | 14 | 2.0 |
| Protein export | 1 | 1.6 | Valine, leucine and isoleucine biosynthesis | 0 | 3.2 |
| Protein export | 14 | 2.2 | Valine, leucine and isoleucine biosynthesis | 14 | 2.2 |
| Purine metabolism | 0 | 2.3 | Valine, leucine and isoleucine degradation | 0 | 2.0 |
| Purine metabolism | 14 | 1.8 | Valine, leucine and isoleucine degradation | 14 | 1.9 |
| Pyrimidine metabolism | 0 | 2.5 | Vibrio cholerae pathogenic cycle | 0 | 1.5 |
| Pyrimidine metabolism | 14 | 2.1 | Vitamin B6 metabolism | 0 | 3.2 |
| Pyruvate metabolism | 0 | 2.3 | Vitamin B6 metabolism | 14 | 2.0 |
| Pyruvate metabolism | 14 | 1.9 | Zeatin biosynthesis | 0 | 2.4 |
| Riboflavin metabolism | 0 | 2.5 | Zeatin biosynthesis | 1 | 1.4 |
| Riboflavin metabolism | 14 | 1.8 | Zeatin biosynthesis | 14 | 1.9 |
| ⎯⎯⎯⎯⎯⎯⎯⎯⎯⎯⎯⎯⎯⎯⎯⎯⎯⎯⎯⎯⎯⎯⎯⎯⎯⎯⎯⎯⎯⎯⎯⎯⎯⎯⎯⎯⎯⎯⎯⎯⎯⎯⎯⎯⎯⎯⎯⎯⎯⎯⎯ | | | | | |

^a^ A: Incubation time (days), ^b^ B: The effect size, which is defined as the difference between groups divided by the maximum dispersion within group X or Y, was calculated with the aldex.ttest argument (ALDEx2 (version, 1.18), Gloor et al. (2020)), ^c^ a positive value means that the relative abundance of the putative metabolic functions was larger in the second treatment than in the first, a negative value the opposite.

| ⎯⎯⎯⎯⎯⎯⎯⎯⎯⎯⎯⎯⎯⎯⎯⎯⎯⎯⎯⎯⎯⎯⎯⎯⎯⎯⎯⎯⎯⎯⎯⎯⎯⎯⎯⎯⎯⎯⎯⎯⎯⎯⎯⎯⎯⎯⎯⎯⎯⎯⎯ |
| --- |
