# Supplementary material for: Young maize plants impact the bacterial community in Australian cotton‐sown vertisol more than agricultural practices
Source: Environ Microbiol Rep. 2025 Apr 30;17(3):e13322. doi: 10.1111/1758-2229.13322 (PMC12041893; doi:10.1111/1758-2229.13322)
Supplement: Supplementary file 16 — Table S6. Effect of application of young maize plants, the neutral detergent fibre (NDF) fraction or urea on the relative abundance of bacterial groups assigned up to the taxonomic level of genus compared their relative abundance in the unamended soils. Only bacterial groups that were significantly affected by treatment are given (p < 0.05). The effect size, which is defined as the difference between groups divided by the maximum dispersion within group X or Y, was calculated with the aldex.ttest argument (ALDEx2 (version, 1.18), Gloor et al. (2020)). [file EMI4-17-e13322-s016.docx]

**Table S6** Effect of application of young maize plants, the neutral detergent fibre (NDF) fraction or urea on the relative abundance of bacterial groups assigned up to the taxonomic level of genus compared their relative abundance in the unamended soils. Only bacterial groups that were significantly affected by treatment are given (*p* < 0.05). The effect size, which is defined as the difference between groups divided by the maximum dispersion within group X or Y, was calculated with the aldex.ttest argument (ALDEx2 (version, 1.18), Gloor et al. (2020)).

| ⎯⎯⎯⎯⎯⎯⎯⎯⎯⎯⎯⎯⎯⎯⎯⎯⎯⎯⎯⎯⎯⎯⎯⎯⎯⎯⎯⎯⎯⎯⎯⎯⎯⎯⎯⎯⎯⎯⎯⎯⎯⎯⎯⎯⎯⎯⎯⎯ | | | |
| --- | --- | --- | --- |
|  | Time ^a^ |  | Effect |
| **Young maize plants amended soils vs unamended soil** | (days) | Soil ^b^ | size ^c^ |
| ⎯⎯⎯⎯⎯⎯⎯⎯⎯⎯⎯⎯⎯⎯⎯⎯⎯⎯⎯⎯⎯⎯⎯⎯⎯⎯⎯⎯⎯⎯⎯⎯⎯⎯⎯⎯⎯⎯⎯⎯⎯⎯⎯⎯⎯⎯⎯⎯ | | | |
| **Actinobacteriota** |  |  |  |
| Actinobacteria, Micrococcales, Micrococcaceae, Pseudarthrobacter | 7 | MITCW | -2.4 |
| Actinobacteria, Micrococcales, Promicromonosporaceae, Promicromonospora | 3 | CTCC | -2.2 |
| Actinobacteria, Micrococcales, Promicromonosporaceae, Promicromonospora | 7 | MITCW | -2.4 |
| Actinobacteria, Micrococcales, Promicromonosporaceae, Promicromonospora | 14 | MITCW | -2.5 |
| Actinobacteria, Propionibacteriales, Nocardioidaceae, Kribbella | 14 | MITCC | -2.5 |
| Actinobacteria, Propionibacteriales, Nocardioidaceae, Kribbella | 28 | MITCW | -2.5 |
| Actinobacteria, Propionibacteriales, Nocardioidaceae, Nocardioides | 3 | MITCC | -1.7 |
| Actinobacteria, Propionibacteriales, Nocardioidaceae, Nocardioides | 7 | MITCW | -2.2 |
| Actinobacteria, Propionibacteriales, Nocardioidaceae, Nocardioides | 14 | MITCW | -2.0 |
| Actinobacteria, Pseudonocardiales, Pseudonocardiaceae, Kibdelosporangium | 1 | MITCW | -2.0 |
| Actinobacteria, Streptomycetales, Streptomycetaceae, Streptomyces | 1 | MITCW | -1.7 |
| Actinobacteria, Streptomycetales, Streptomycetaceae, Streptomyces | 3 | MITCC | -2.4 |
| Actinobacteria, Streptomycetales, Streptomycetaceae, Streptomyces | 3 | MITCW | -3.4 |
| Actinobacteria, Streptomycetales, Streptomycetaceae, Streptomyces | 7 | MITCW | -3.2 |
| Actinobacteria, Streptomycetales, Streptomycetaceae, Streptomyces | 14 | MITCC | -2.4 |
| Actinobacteria, Streptomycetales, Streptomycetaceae, Streptomyces | 14 | MITCW | -2.1 |
| Actinobacteria, Streptomycetales, Streptomycetaceae, Streptomyces | 28 | MITCW | -1.8 |
| **Bacillota** |  |  |  |
| Bacilli, Bacillales, Salisediminibacteriaceae, Salipaludibacillus | 1 | CTCC | -3.2 |
| **Pseudomonadata, Alphaproteobacteria** |  |  |  |
| Caulobacterales, Caulobacteraceae, Phenylobacterium | 3 | CTCC | 2.5 |
| **Pseudomonadata, Gammaproteobacteria** |  |  |  |
| Enterobacterales, Enterobacteriaceae | 3 | CTCC | -2.1 |
| Enterobacterales, Enterobacteriaceae | 3 | MITCW | -2.9 |
| Enterobacterales, Enterobacteriaceae | 7 | MITCW | -1.9 |
| Gammaproteobacteria_Incertae_Sedis, Unknown_Family, Acidibacter | 3 | MITCC | 1.9 |
| Oceanospirillales, Halomonadaceae, Halomonas | 1 | CTCC | -5.1 |
| Pseudomonadales, Moraxellaceae, Acinetobacter | 1 | CTCC | -3.8 |
| Pseudomonadales, Moraxellaceae, Acinetobacter | 1 | MITCW | -2.5 |
| Pseudomonadales, Moraxellaceae, Acinetobacter | 3 | CTCC | -2.7 |
| Pseudomonadales, Moraxellaceae, Acinetobacter | 3 | MITCC | -3.1 |
| Pseudomonadales, Moraxellaceae, Acinetobacter | 3 | MITCW | -2.8 |
| Pseudomonadales, Moraxellaceae, Acinetobacter | 14 | MITCC | -3.0 |
| Pseudomonadales, Moraxellaceae, Acinetobacter | 14 | MITCW | -2.5 |
| Pseudomonadales, Pseudomonadaceae, Pseudomonas | 3 | CTCC | -2.5 |
| Pseudomonadales, Pseudomonadaceae, Pseudomonas | 3 | MITCC | -2.7 |
| Pseudomonadales, Pseudomonadaceae, Pseudomonas | 7 | MITCW | -2.5 |
| Xanthomonadales, Xanthomonadaceae, Stenotrophomonas | 1 | CTCC | -3.0 |
| Xanthomonadales, Xanthomonadaceae, Stenotrophomonas | 3 | CTCC | -2.4 |
| ⎯⎯⎯⎯⎯⎯⎯⎯⎯⎯⎯⎯⎯⎯⎯⎯⎯⎯⎯⎯⎯⎯⎯⎯⎯⎯⎯⎯⎯⎯⎯⎯⎯⎯⎯⎯⎯⎯⎯⎯⎯⎯⎯⎯⎯⎯⎯⎯ | | | |

**Table S6** Continued.

| ⎯⎯⎯⎯⎯⎯⎯⎯⎯⎯⎯⎯⎯⎯⎯⎯⎯⎯⎯⎯⎯⎯⎯⎯⎯⎯⎯⎯⎯⎯⎯⎯⎯⎯⎯⎯⎯⎯⎯⎯⎯⎯⎯⎯⎯⎯⎯⎯ | | | |
| --- | --- | --- | --- |
| **Neutral detergent fibre fraction amended soil vs unamended soil** |  |  |  |
| ⎯⎯⎯⎯⎯⎯⎯⎯⎯⎯⎯⎯⎯⎯⎯⎯⎯⎯⎯⎯⎯⎯⎯⎯⎯⎯⎯⎯⎯⎯⎯⎯⎯⎯⎯⎯⎯⎯⎯⎯⎯⎯⎯⎯⎯⎯⎯⎯ | | | |
| **Acidobacteriota** |  |  |  |
| Blastocatellia, Pyrinomonadales, Pyrinomonadaceae, RB41 | 14 | MITWC | 3.4 |
| Vicinamibacteria, Vicinamibacterales, Vicinamibacteraceae, Vicinamibacteraceae | 1 | MITWC | 1.7 |
| Actinobacteriota |  |  |  |
| Actinobacteria, Corynebacteriales, Nocardiaceae, Rhodococcus | 3 | MITWC | -2.4 |
| Actinobacteria, Corynebacteriales, Nocardiaceae, Rhodococcus | 7 | MITWC | -2.6 |
| Actinobacteria, Micrococcales, Micrococcaceae | 28 | MITWC | -3.0 |
| Actinobacteria, Micrococcales, Promicromonosporaceae, Promicromonospora | 14 | CTCC | -1.7 |
| Actinobacteria, Propionibacteriales, Nocardioidaceae, Marmoricola | 14 | MITWC | -2.3 |
| Actinobacteria, Propionibacteriales, Nocardioidaceae, Nocardioides | 1 | MITWC | -2.6 |
| Actinobacteria, Propionibacteriales, Nocardioidaceae, Nocardioides | 3 | MITWC | -2.4 |
| Actinobacteria, Propionibacteriales, Nocardioidaceae, Nocardioides | 7 | MITWC | -2.2 |
| Actinobacteria, Propionibacteriales, Nocardioidaceae, Nocardioides | 14 | MITCC | -2.5 |
| Actinobacteria, Propionibacteriales, Nocardioidaceae, Nocardioides | 14 | MITWC | -2.5 |
| Actinobacteria, Streptomycetales, Streptomycetaceae, Streptomyces | 7 | MITWC | -2.4 |
| Actinobacteria, Streptomycetales, Streptomycetaceae, Streptomyces | 14 | MITCC | -2.4 |
| Actinobacteria, Streptomycetales, Streptomycetaceae, Streptomyces | 14 | MITWC | -1.7 |
| **Chloroflexota** |  |  |  |
| Anaerolineae, Anaerolineales, Anaerolineaceae | 1 | MITWC | 2.4 |
| **Pseudomonadata, Gammaproteobacteria** |  |  |  |
| Enterobacterales, Enterobacteriaceae | 3 | MITWC | -2.7 |
| Oceanospirillales, Halomonadaceae, Halomonas | 1 | MITCC | -2.8 |
| Oceanospirillales, Halomonadaceae, Halomonas | 1 | MITWC | -4.9 |
| Pseudomonadales, Moraxellaceae, Acinetobacter | 14 | CTCC | -2.4 |
| Pseudomonadales, Pseudomonadaceae, Pseudomonas | 3 | MITWC | -3.3 |
| Xanthomonadales, Xanthomonadaceae, Stenotrophomonas | 3 | MITWC | -2.7 |
| Xanthomonadales, Xanthomonadaceae, Stenotrophomonas | 14 | CTCC | -2.8 |
| ⎯⎯⎯⎯⎯⎯⎯⎯⎯⎯⎯⎯⎯⎯⎯⎯⎯⎯⎯⎯⎯⎯⎯⎯⎯⎯⎯⎯⎯⎯⎯⎯⎯⎯⎯⎯⎯⎯⎯⎯⎯⎯⎯⎯⎯⎯⎯⎯ | | | |
| **Urea amended soil vs unamended soil** |  |  |  |
| ⎯⎯⎯⎯⎯⎯⎯⎯⎯⎯⎯⎯⎯⎯⎯⎯⎯⎯⎯⎯⎯⎯⎯⎯⎯⎯⎯⎯⎯⎯⎯⎯⎯⎯⎯⎯⎯⎯⎯⎯⎯⎯⎯⎯⎯⎯⎯⎯ | | | |
| **Acidobacteriota** |  |  |  |
| Blastocatellia, Blastocatellales, Blastocatellaceae, uncultured | 28 | MITCC | -2.3 |
| Actinobacteria, Propionibacteriales, Nocardioidaceae, *Nocardioides* | 1 | CTCC | -2.2 |
| **Pseudomonadata, Alphaproteobacteria** |  |  |  |
| Reyranellales, Reyranellaceae, *Reyranella* | 3 | MITCC | 2.0 |
| **Pseudomonadata, Gammaproteobacteria** |  |  |  |
| Burkholderiales, Nitrosomonadaceae, *MND1* | 14 | MITCC | -2.6 |
| Burkholderiales, Oxalobacteraceae, *Noviherbaspirillum* | 3 | MITCC | -2.3 |
| Gammaproteobacteria_Incertae_Sedis, Unknown_Family, *Acidibacter* | 3 | MITCC | -2.3 |
| Pseudomonadales, Moraxellaceae, *Acinetobacter* | 7 | MITWC | -3.0 |
| Xanthomonadales, Xanthomonadaceae, *Lysobacter* | 14 | MITWC | -2.2 |
| ⎯⎯⎯⎯⎯⎯⎯⎯⎯⎯⎯⎯⎯⎯⎯⎯⎯⎯⎯⎯⎯⎯⎯⎯⎯⎯⎯⎯⎯⎯⎯⎯⎯⎯⎯⎯⎯⎯⎯⎯⎯⎯⎯⎯⎯⎯⎯⎯ | | | |

^a^ Aerobic incubation time, ^b^ CTCC: Soil cultivated with cotton (*Gossypium hirsutum* L.) monoculture (summer cotton-winter, fallow-summer cotton) conventional tillage, MITCC: minimum tillage of continuous cotton (MITCC) and MITCW: minimum tillage cotton-wheat (*Triticum aestivum* L.) rotation (summer cotton-winter wheat-summer and winter fallow-summer cotton), ^c^ a positive value means the relative abundance of the bacterial group was larger in the unamended soil than in the soil amended with young maize plants, the neutral detergent fibre (NDF) fraction or urea while a negative value the opposite.

| ⎯⎯⎯⎯⎯⎯⎯⎯⎯⎯⎯⎯⎯⎯⎯⎯⎯⎯⎯⎯⎯⎯⎯⎯⎯⎯⎯⎯⎯⎯⎯⎯⎯⎯⎯⎯⎯⎯⎯⎯⎯⎯⎯⎯⎯⎯⎯⎯ |
| --- |
