# Supplementary material for: Young maize plants impact the bacterial community in Australian cotton‐sown vertisol more than agricultural practices
Source: Environ Microbiol Rep. 2025 Apr 30;17(3):e13322. doi: 10.1111/1758-2229.13322 (PMC12041893; doi:10.1111/1758-2229.13322)
Supplement: Supplementary file 17 — Table S7. The number of putative metabolic functions with an effect size (≤ −0.8 and ≥0.8) when comparing the relative abundance of putative metabolic functions separately in soil cultivated with cotton (Gossypium hirsutum L.) monoculture (summer cotton‐winter, fallow‐summer cotton) conventional tillage (CTCC), minimum tillage of continuous cotton (MITCC), and minimum tillage cotton‐wheat (Triticum aestivum L.) rotation (summer cotton‐winter wheat‐summer and winter fallow‐summer cotton) (MITCW) with the same soils amended with maize or its neutral detergent fibre (NDF) fraction after 1, 3, 7, 14, or 28 days of an aerobic incubation. [file EMI4-17-e13322-s012.docx]

**Table S7**. The number of putative metabolic functions with an effect size (≤ -0.8 and ≥ 0.8) when comparing the relative abundance of putative metabolic functions separately in soil cultivated with cotton (*Gossypium hirsutum* L.) monoculture (summer cotton-winter, fallow-summer cotton) conventional tillage (CTCC), minimum tillage of continuous cotton (MITCC), and minimum tillage cotton-wheat (*Triticum aestivum* L.) rotation (summer cotton-winter wheat-summer and winter fallow-summer cotton) (MITCW) with the same soils amended with maize or its neutral detergent fibre (NDF) fraction after 1, 3, 7, 14 or 28 days of an aerobic incubation.

| ⎯⎯⎯⎯⎯⎯⎯⎯⎯⎯⎯⎯⎯⎯⎯⎯⎯⎯⎯⎯⎯⎯⎯⎯⎯⎯⎯⎯⎯⎯⎯⎯⎯⎯⎯⎯⎯⎯⎯⎯⎯⎯⎯⎯⎯⎯⎯⎯⎯⎯⎯⎯⎯⎯⎯⎯⎯⎯⎯⎯ | | | | | | | | | |
| --- | --- | --- | --- | --- | --- | --- | --- | --- | --- |
|  | Maize | | NDF | |  | Maize | | NDF | |
|  | ⎯⎯⎯ | | ⎯⎯⎯ | |  | ⎯⎯⎯ | | ⎯⎯⎯ | |
| Putative metabolic functions | A | B | A | B | Putative metabolic functions | A | B | A | B |
| ⎯⎯⎯⎯⎯⎯⎯⎯⎯⎯⎯⎯⎯⎯⎯⎯⎯⎯⎯⎯⎯⎯⎯⎯⎯⎯⎯⎯⎯⎯⎯⎯⎯⎯⎯⎯⎯⎯⎯⎯⎯⎯⎯⎯⎯⎯⎯⎯⎯⎯⎯⎯⎯⎯⎯⎯⎯⎯⎯⎯ | | | | | | | | | |
| Alanine, aspartate and glutamate metabolism | 3 | 12 | 3 | 9 | Chlorocyclohexane and chlorobenzene degradation | 3 | 9 | 3 | 3 |
| Amino sugar and nucleotide sugar metabolism | 3 | 11 | 3 | 9 | Citrate cycle (TCA cycle) | 3 | 10 | 3 | 8 |
| Aminoacyl-tRNA biosynthesis | 3 | 12 | 3 | 11 | Cysteine and methionine metabolism | 3 | 12 | 3 | 10 |
| Apoptosis | 3 | 9 | 14 | 4 | D-Alanine metabolism | 3 | 12 | 3 | 9 |
| Arginine and proline metabolism | 3 | 11 | 3 | 7 | D-Arginine and D-ornithine metabolism | 1 | 13 | 1 | 12 |
| Bacterial chemotaxis | 3 | 11 | 3 | 7 | D-Glutamine and D-glutamate metabolism | 3 | 12 | 3 | 11 |
| **Bacterial secretion system** ^a^ | 3 | 11 | 3 | 12 | Dioxin degradation | 1 | 7 | 1 | 4 |
| Base excision repair | 3 | 10 | 3 | 6 | DNA replication | 3 | 12 | 3 | 10 |
| beta-Lactam resistance | 1 | 13 | 7 | 3 | Drug metabolism - other enzymes | 3 | 10 | 3 | 7 |
| Betalain biosynthesis | 1 | 12 | 1 | 9 | Fatty acid biosynthesis | 3 | 12 | 3 | 10 |
| Biosynthesis ansamycins | 1 | 12 | 1 | 12 | Flagellar assembly | 3 | 10 | 3 | 7 |
| Biosynthesis siderophore group nonribosomal peptides | 1 | 13 | 3 | 10 | Flavonoid biosynthesis | 1 | 12 | 1 | 7 |
| Biosynthesis type II polyketide backbone | 1 | 11 | 3 | 8 | Fluorobenzoate degradation | 1 | 8 | 1 | 8 |
| Biosynthesis unsaturated fatty acids | 3 | 9 | 3 | 5 | Folate biosynthesis | 3 | 12 | 3 | 8 |
| Biosynthesis vancomycin group antibiotics | 1 | 14 | 3 | 10 | Fructose and mannose metabolism | 3 | 11 | 3 | 6 |
| Biotin metabolism | 3 | 10 | 3 | 9 | Glutathione metabolism | 3 | 10 | 3 | 7 |
| **Bisphenol degradation** |  |  | 3 | 5 | Glycerophospholipid metabolism | 3 | 11 | 3 | 7 |
| Butanoate metabolism | 3 | 7 | 3 | 5 | Glycine, serine and threonine metabolism | 3 | 11 | 3 | 9 |
| C5-Branched dibasic acid metabolism | 3 | 12 | 3 | 10 | Glycosaminoglycan degradation | 1 | 13 | 3 | 7 |
| **Caprolactam degradation** | 1 | 7 | 1 | 11 | Glyoxylate and dicarboxylate metabolism | 3 | 10 | 3 | 7 |
| Carbon fixation in photosynthetic organisms | 3 | 12 | 3 | 10 | Histidine metabolism | 3 | 12 | 3 | 9 |
| Carbon fixation pathways in prokaryotes | 3 | 12 | 3 | 10 | Homologous recombination | 3 | 12 | 3 | 10 |
| Cell cycle - Caulobacter | 3 | 12 | 3 | 10 | Linoleic acid metabolism | 1 | 5 | 1 | 9 |
| ⎯⎯⎯⎯⎯⎯⎯⎯⎯⎯⎯⎯⎯⎯⎯⎯⎯⎯⎯⎯⎯⎯⎯⎯⎯⎯⎯⎯⎯⎯⎯⎯⎯⎯⎯⎯⎯⎯⎯⎯⎯⎯⎯⎯⎯⎯⎯⎯⎯⎯⎯⎯⎯⎯⎯⎯⎯⎯⎯⎯ | | | | | | | | | |

**Table S7.** Continued.

| ⎯⎯⎯⎯⎯⎯⎯⎯⎯⎯⎯⎯⎯⎯⎯⎯⎯⎯⎯⎯⎯⎯⎯⎯⎯⎯⎯⎯⎯⎯⎯⎯⎯⎯⎯⎯⎯⎯⎯⎯⎯⎯⎯⎯⎯⎯⎯⎯⎯⎯⎯⎯⎯⎯⎯⎯⎯⎯⎯⎯ | | | | | | | | | |  |
| --- | --- | --- | --- | --- | --- | --- | --- | --- | --- | --- |
| Lipoic acid metabolism | 3 | 12 | 3 | 10 | Pyrimidine metabolism | 3 | 12 | 3 | 10 |  |
| Lipopolysaccharide biosynthesis | 3 | 12 | 3 | 12 | Pyruvate metabolism | 3 | 11 | 3 | 7 |  |
| Lysine biosynthesis | 3 | 12 | 3 | 10 | Riboflavin metabolism | 3 | 12 | 3 | 9 |  |
| Methane metabolism | 3 | 10 | 3 | 6 | Ribosome | 3 | 12 | 3 | 11 |  |
| Mismatch repair | 3 | 12 | 3 | 10 | RNA degradation | 3 | 12 | 3 | 10 |  |
| Nicotinate and nicotinamide metabolism | 3 | 12 | 3 | 9 | RNA polymerase | 1 | 14 | 1 | 11 |  |
| Nitrogen metabolism | 3 | 10 | 3 | 7 | RNA transport | 3 | 10 | 3 | 6 |  |
| Non-homologous end-joining | 3 | 5 | 7 | 7 | Secondary bile acid biosynthesis | 1 | 10 | 3 | 3 |  |
| Nucleotide excision repair | 3 | 12 | 3 | 6 | Selenocompound metabolism | 3 | 12 | 3 | 9 |  |
| One carbon pool by folate | 3 | 12 | 3 | 9 | Sesquiterpenoid biosynthesis | 3 | 10 | 7 | 7 |  |
| Other glycan degradation | 3 | 8 | 1 | 7 | Sphingolipid metabolism | 3 | 7 | 7 | 4 |  |
| Oxidative phosphorylation | 3 | 12 | 3 | 11 | Steroid biosynthesis | 3 | 11 | 7 | 7 |  |
| Pantothenate and CoA biosynthesis | 3 | 12 | 3 | 10 | Steroid hormone biosynthesis | 3 | 9 | 7 | 8 |  |
| Pentose phosphate pathway | 3 | 10 | 3 | 7 | Streptomycin biosynthesis | 1 | 13 | 3 | 10 |  |
| Peptidoglycan biosynthesis | 3 | 12 | 3 | 11 | Sulfur relay system | 3 | 12 | 3 | 10 |  |
| Phenylalanine, tyrosine and tryptophan biosynthesis | 3 | 12 | 3 | 10 | Taurine and hypotaurine metabolism | 3 | 12 | 3 | 10 |  |
| Photosynthesis | 1 | 13 | 3 | 11 | Terpenoid backbone biosynthesis | 3 | 10 | 3 | 7 |  |
| Plant-pathogen interaction | 3 | 10 | 3 | 7 | Thiamine metabolism | 3 | 12 | 3 | 10 |  |
| Porphyrin and chlorophyll metabolism | 3 | 11 | 3 | 7 | Toluene degradation | 3 | 11 | 3 | 9 |  |
| Propanoate metabolism | 3 | 12 | 3 | 7 | Tropane, piperidine and pyridine alkaloid biosynthesis | 3 | 10 | 3 | 6 |  |
| Proteasome | 3 | 11 | 3 | 8 | Two-component system | 3 | 10 | 3 | 7 |  |
| Protein digestion and absorption | 1 | 12 | 1 | 9 | Ubiquinone and other terpenoid-quinone biosynthesis | 3 | 11 | 3 | 9 |  |
| Protein export | 3 | 12 | 3 | 11 | Valine, leucine and isoleucine biosynthesis | 3 | 12 | 3 | 10 |  |
| Protein processing in endoplasmic reticulum | 3 | 12 | 3 | 6 | Vitamin B6 metabolism | 3 | 12 | 3 | 8 |  |
| Purine metabolism | 3 | 12 | 3 | 9 | Zeatin biosynthesis | 1 | 13 | 3 | 11 |  |
| ⎯⎯⎯⎯⎯⎯⎯⎯⎯⎯⎯⎯⎯⎯⎯⎯⎯⎯⎯⎯⎯⎯⎯⎯⎯⎯⎯⎯⎯⎯⎯⎯⎯⎯⎯⎯⎯⎯⎯⎯⎯⎯⎯⎯⎯⎯⎯⎯⎯⎯⎯⎯⎯⎯⎯⎯⎯⎯⎯⎯ | | | | | | | | | | |

^a^ Putative metabolic functions given in bold font were more affected in the NDF amended soils than in the young maize plants amended soil, ^b^ The relative abundance of the putative metabolic functions in underlined font increased in the NDF and young maize plants amended soils compared to the unamended soils. The relative abundance of all other putative metabolic functions was lower in the NDF and young maize plants amended soils compared to the unamended soils.
