# Supplementary material for: Young maize plants impact the bacterial community in Australian cotton‐sown vertisol more than agricultural practices
Source: Environ Microbiol Rep. 2025 Apr 30;17(3):e13322. doi: 10.1111/1758-2229.13322 (PMC12041893; doi:10.1111/1758-2229.13322)
Supplement: Supplementary file 18 — Table S8. The number of putative metabolic functions (Function) and bacterial groups assigned up to the taxonomic level of genus (Genera) with an effect size (≤ −0.8 and ≥0.8) when comparing the relative abundance of putative metabolic functions and bacterial genera in soil cultivated with cotton (Gossypium hirsutum L.) monoculture (summer cotton‐winter, fallow‐summer cotton) conventional tillage (CTCC), minimum tillage of continuous cotton (MITCC), and minimum tillage cotton‐wheat (Triticum aestivum L.) rotation (summer cotton‐winter wheat‐summer and winter fallow‐summer cotton) (MITCW) at the onset of the experiment and after 1, 3, 7, 14 or 28 days of aerobic incubation. [file EMI4-17-e13322-s006.docx]

**Table S8** The number of putative metabolic functions (Function) and bacterial groups assigned up to the taxonomic level of genus (Genera) with an effect size (≤ -0.8 and ≥ 0.8) when comparing the relative abundance of putative metabolic functions and bacterial genera in soil cultivated with cotton (*Gossypium hirsutum* L.) monoculture (summer cotton-winter, fallow-summer cotton) conventional tillage (CTCC), minimum tillage of continuous cotton (MITCC), and minimum tillage cotton-wheat (*Triticum aestivum* L.) rotation (summer cotton-winter wheat-summer and winter fallow-summer cotton) (MITCW) at the onset of the experiment and after 1, 3, 7, 14 or 28 days of aerobic incubation.

| ⎯⎯⎯⎯⎯⎯⎯⎯⎯⎯⎯⎯⎯⎯⎯⎯⎯⎯⎯⎯⎯⎯⎯⎯⎯⎯⎯⎯⎯⎯⎯⎯⎯⎯⎯⎯⎯⎯⎯⎯⎯⎯⎯⎯⎯⎯⎯⎯⎯⎯⎯⎯⎯⎯⎯ | | | | | | | |
| --- | --- | --- | --- | --- | --- | --- | --- |
|  | | CTCC vs MITCC | | CTCC vs MITCW | | MITCC vs MITCW | |
|  | | ⎯⎯⎯⎯⎯⎯⎯⎯⎯⎯⎯⎯⎯⎯ | | ⎯⎯⎯⎯⎯⎯⎯⎯⎯⎯⎯⎯⎯⎯ | | ⎯⎯⎯⎯⎯⎯⎯⎯⎯⎯⎯⎯⎯⎯ | |
| Time (days) | | Genera | Function | Genera | Function | Genera | Function |
| ⎯⎯⎯⎯⎯⎯⎯⎯⎯⎯⎯⎯⎯⎯⎯⎯⎯⎯⎯⎯⎯⎯⎯⎯⎯⎯⎯⎯⎯⎯⎯⎯⎯⎯⎯⎯⎯⎯⎯⎯⎯⎯⎯⎯⎯⎯⎯⎯⎯⎯⎯⎯⎯⎯⎯ | | | | | | | |
| 0 | | 20 | 35 | 15 | 18 | 31 | 106 |
| 1 | | 25 | 48 | 20 | 51 | 14 | 69 |
| 3 | | 22 | 81 | 18 | 17 | 20 | 9 |
| 7 | | 22 | 19 | 21 | 57 | 25 | 13 |
| 14 | | 35 | 9 | 28 | 123 | 17 | 114 |
| 28 | | 28 | 3 | 26 | 6 | 21 | 2 |
| ⎯⎯⎯⎯⎯⎯⎯⎯⎯⎯⎯⎯⎯⎯⎯⎯⎯⎯⎯⎯⎯⎯⎯⎯⎯⎯⎯⎯⎯⎯⎯⎯⎯⎯⎯⎯⎯⎯⎯⎯⎯⎯⎯⎯⎯⎯⎯⎯⎯⎯⎯⎯⎯⎯ | | | | | | | |
|  | | | | | | | |
